# Biodiversity, complete inventory, taxonomy and keys to the species of Mysidae (Crustacea, Mysida) from marine caves of the Caribbean with revision of three genera and description of three new species

**DOI:** 10.3897/zookeys.1283.169111

**Published:** 2026-06-24

**Authors:** Karl J. Wittmann, Pierre Chevaldonné

**Affiliations:** 1 Department of Environmental Health, Medical University of Vienna, Kinderspitalgasse 15, 1090 Vienna, Austria Department of Environmental Health, Medical University of Vienna Vienna Austria; 2 IMBE, CNRS, IRD, Aix Marseille Université, Avignon Université, Station Marine d’Endoume, Rue de la Batterie des Lions, 13007 Marseille, France IMBE, CNRS, IRD, Aix Marseille Université, Avignon Université, Station Marine d’Endoume Marseille France

**Keywords:** 18S rDNA, distribution, endemism, marine speleology, mtCOI DNA, taxonomy, troglophilia

## Abstract

Collections of Mysidae taken in marine caves during the PACOTILLES-2 cruise to the Lesser Antilles, together with additional sampling in the Caribbean and inspection of museum collections and literature data yielded five stygobiont, 13 troglophile (stygophile) and nine trogloxene species. Sequences for COI mtDNA were obtained for populations of six species and nuclear 18S ribosomal DNA for populations of two species. A COI-based distance tree is given for populations of three species of *Mysidium*.

The tribe Mysidetini is here revised with inclusion of the genera *Bermudamysis* and *Platyops* based on segmental numbers and the non-prehensile structure of thoracic endopod 3. The description of *Bermudamysis caribbaea***sp. nov**. from Guadeloupe is mainly based on segmental numbers of thoracic endopods and on telson structure. The male of *Platyops sterreri* is now first described from Guadeloupe, which represents the first record of this species outside Bermuda. *Platyops dennisi* is removed from *Platyops* and established as the type species of *Chelitrapezura***gen. nov**., the latter affiliated with the tribe Heteromysini (subfamily Heteromysinae). *Heteromysis troglophila***sp. nov**. from marine caves in the Lesser Antilles differs from the nearest congeners by a broadly rounded rostrum in combination with different numbers of spines and laminae of the telson. The genus *Palaumysis* is revised including supplementary descriptions of *P. simonae* and *P. bahamensis*. The establishment of *P. antillensis***sp. nov**. is mainly based on features of the antennula, antenna, and carapace.

Diagnoses together with data on distribution and troglophilia are given for all here acknowledged 27 species from marine and anchialine caves of the Caribbean; keys to the genera and species are included.

## Introduction

The waters of the Caribbean are recognized as a major hotspot of marine biodiversity. This also concerns the Mysidae (Crustacea: Mysida) in the Caribbean *sensu lato*, here including the Bahamas and both Antillean archipelagos but excluding the Gulf of Mexico coast of Cuba and entire Florida, and also not counting limnic, planktonic, and pelagic species. Only two marine (anchialine) cave-dwelling species (Creaser 1936; W.M. Tattersall 1951) and seven non-cavernicolous coastal species were recognized until the 1960s (Brattegard 1969). For the non-cavernicolous species this scenario changed considerably with the admirable work of Torleiv Brattegard, who pushed the number to 53 species within only 12 years (Brattegard 1969, 1970a, 1970b, 1973, 1974a, 1974b, 1975, 1980). Only three additional cave-dwelling species were described from the study area during this period (Băcescu and Orghidan 1971, 1977; Bowman 1977). In recent years, increasing research efforts have been invested in improving our knowledge about marine troglobiont and stygobiont species, yielding a total of 24 cave-dwelling species by 2023 (Bowman 1985; Băcescu 1991; Pesce et al. 1994; Ortiz et al. 1997; Pesce and Iliffe 2002; Hanamura and Kase 2004; Price and Heard 2004; Wittmann and Wirtz 2019; Wittmann 2023). Herein, with the addition of three new species, we present distribution data and combined textual and pictorial diagnoses together with keys for the resulting total of 27 species of cave-dwelling Mysidae of the Caribbean. In addition, world-wide keys are given for three revised genera (*Bermudamysis* Băcescu & Iliffe, 1986, *Platyops* Băcescu & Iliffe, 1986, and *Palaumysis* Băcescu & Iliffe, 1986). Revision of these genera requires the study of non-Caribbean species, namely *Bermudamysis speluncola* Băcescu & Iliffe, 1986, from Bermuda and *Palaumysis simonae* Băcescu & Iliffe, 1986, from the Palau Archipelago.

The main part of the present study was triggered by the “PACOTILLES 2" research cruise organized in 2015 to survey, by SCUBA diving, the marine biodiversity of marine caves and littoral sponges at several islands of the Lesser Antilles from the Grenadines to Anguilla (Fauvelot 2015; Pérez 2015; Chevaldonné et al. 2018). Among the caves richest in taxonomic and bionomic novelties, the Full Moon Cave in Bequia, the Cathedral Cave in Guadeloupe, and the Zeb cave in Martinique harbored ‘semi-dark’ to ‘dark cave’ sponge community gradients and exhibited a rich compartment of mobile crustaceans. The Amedien cave in Guadeloupe and the Bat Cave at St. Vincent also yielded important contributions to biodiversity. Additional valuable data on Caribbean biodiversity were obtained by an independent sampling campaign in marine caves and other habitats at the island of Curaçao in 2014.

Mitochondrial DNA coding for Cytochrome Oxidase I (COI) and nuclear 18S ribosomal DNA (18S) were sequenced as part of the “PACOTILLES 2" material as a contribution to the molecular systematics and phylogeography of cave mysids. A distance tree was built for COI data of three species of *Mysidium*, thus visualizing the differentiation between these taxa previously established based solely on morphological methods.

## Materials and methods

### Field materials

**Figure 1.**
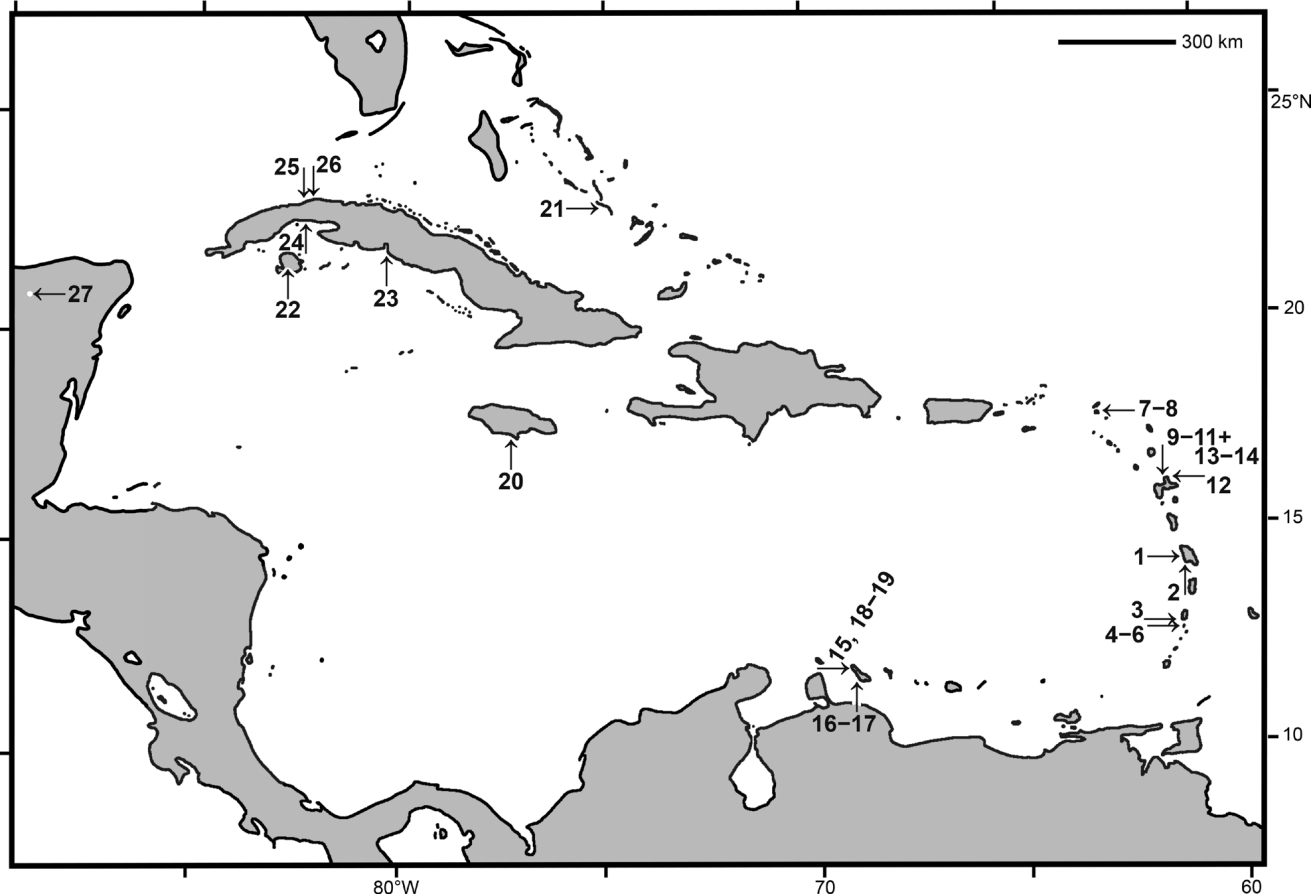
Sampling localities of Mysidae from marine and anchialine caves of the Caribbean (Stations 1–19) and adjacent areas; including material studied from museum collections and other sources (Stations 20–27); station details in Table 1.

The distribution of caves in the Caribbean and adjacent areas investigated for Mysidae species is shown in Fig. 1 (station details in Table 1). As the greatest contribution, samples from Stations 1–14 were taken by P.C. with modified Sket bottles (Chevaldonné et al. 2008) upon SCUBA diving in marine caves of the Caribbean. This encompasses the islands of the Lesser Antilles (Bequia, St. Vincent, Martinique, Guadeloupe, Saint-Martin) in May 2015, during the ‘PACOTILLES 2' (https://doi.org/10.17600/15005300) research cruise on board R/V Antea (Pérez 2015). Peter Wirtz (Madeira) and K.J.W. collected additional materials from marine caves and other habitats at the island of Curaçao with diver-operated hand nets (Stations 15–19).

**Table 1.** Detailed sampling data from marine caves of the Caribbean with supplements from other sea areas.

Station #	Locality	Coordinates	Depth (m)	Date (local time)	Sample description	Species recorded
**1**	Caribbean, Lesser Antilles, Martinique, Anses d’Arlet, Anse Noire Cave	14°31.70'N, 61°05.30'W	4	14 May 2015, morning	PACOTILLES-2 station #545, semi-dark part of marine cave, mysids forming a small group, leg. P.C.	*Heteromysis troglophila* sp. nov.
**2**	Caribbean, Lesser Antilles, Martinique, Zeb Cave	14°27.83'N, 61°01.07'W	20	15 May 2015, morning	PACOTILLES-2 station #547, mysid swarm in entrance of marine cave, leg. P.C.	*Mysidium gracile*, *M. integrum*, *M. columbiae*
**3**	Caribbean, Lesser Antilles, St Vincent, Bat Cave	13°11.28'N, 61°16.17'W	10	18 May 2015, morning	PACOTILLES-2 station #553, entrance of marine cave, leg. P.C.	*Mysidium gracile*
**4**	Caribbean, Lesser Antilles, The Grenadines, SW-Bequia, W of Admiralty Bay, Full Moon Cave	12°59.52'N, 61°16.73'W	13	19 May 2015, morning	PACOTILLES-2 station #555, sampled with Sket bottle in darkest area of marine cave, huge swarms, leg. P.C.	*Palaumysis antillensis* sp. nov.
**5**	Caribbean, Lesser Antilles, The Grenadines, SW-Bequia, W of Admiralty Bay, Full Moon Cave	12°59.52'N, 61°16.73'W	13	19 May 2015, afternoon	PACOTILLES-2 station #556a, semi-dark part of marine cave, mysids in small groups, leg. P.C.	*Parvimysis laminata*
**6**	Caribbean, Lesser Antilles, The Grenadines, SW-Bequia, W of Admiralty Bay, Full Moon Cave	12°59.52'N, 61°16.73'W	13	19 May 2015, afternoon	PACOTILLES-2 station #556b, marine, mid-cave swarm, leg. P.C.	*Mysidium antillarum*, *M. columbiae*
**7**	Caribbean, Lesser Antilles, Saint-Martin, Circus	18°06.51'N, 62°59.00'W	12	26 May 2015, morning	PACOTILLES-2 station #565, marine, semi-dark areas, from small holes, leg. P.C.	*Mysidium gracile*
**8**	Caribbean, Lesser Antilles, Saint-Martin, Basse Espagnole	18°07.82'N, 63°00.27'W	15	27 May 2015, morning	PACOTILLES-2 station #567, marine, semi-dark areas, from small holes, leg. P.C.	*Mysidium gracile*, *M. integrum*, *M. triangulare*
**9**	Caribbean, Lesser Antilles, Guadeloupe, Pointe Fontaine, Grande Terre, north of Port-Louis, Cathedral Cave	16°27.74'N, 61°31.84'W	18	29 May 2015, afternoon	PACOTILLES-2 station #571a, semi-dark part of marine cave, mysids in small groups, leg. P.C.	*Parvimysis laminata*
**10**	Caribbean, Lesser Antilles, Guadeloupe, Pointe Fontaine, Grande Terre, north of Port-Louis, Cathedral Cave	16°27.74'N, 61°31.84'W	18	29 May 2015, afternoon	PACOTILLES-2 station #571b, marine, cave entrance swarm, leg. P.C.	*Mysidium columbiae*, *M. integrum*
**11**	Caribbean, Lesser Antilles, Guadeloupe, Pointe Fontaine, Grande Terre, north of Port-Louis, Barracuda Cave	16°27.30'N, 61°32.25'W	19	30 May 2015, afternoon	PACOTILLES-2 station #573, semi-dark part of marine cave, mysids in small groups, leg. P.C.	*Bermudamysis caribbaea* sp. nov., *Mysidium columbiae*, *Parvimysis laminata*
**12**	Caribbean, Lesser Antilles, Guadeloupe, Pointe de la Grande Vigie, Grande Terre, Amedien Cave	16°30.72'N, 61°27.97'W	6	31 May 2015, morning	PACOTILLES-2 station #574, dark part of marine cave, mysids solitary, leg. P.C.	*Heteromysis troglophila* sp. nov.
**13**	Caribbean, Lesser Antilles, Guadeloupe, Pointe Fontaine, Grande Terre, north of Port-Louis, Cathedral Cave	16°27.74'N, 61°31.84'W	18	31 May 2015, afternoon	PACOTILLES-2 station #575-1, sampled in darkest area of marine cave, huge swarms, leg. P.C.	*Palaumysis antillensis* sp. nov.
**14**	Caribbean, Lesser Antilles, Guadeloupe, Pointe Fontaine, Grande Terre, north of Port-Louis, Cathedral Cave	16°27.74'N, 61°31.84'W	18	31 May 2015, afternoon	PACOTILLES-2 station #575-2, dark part of marine cave, together with thousands of *Palaumysis antillensis*, leg. P.C.	*Platyops sterreri*
**15**	Caribbean, Leeward Antilles, west coast of Curaçao, Blue Chamber (The Cove)	12°17.93'N, 69°09.26'W	3	25 Feb. 2014, about 14:40, day	Mysid swarms in rock recesses and in an intertidal marine cave, leg. K.J.W.	*Mysidium integrum*, *M. triangulare*, *Parvimysis laminata*
**16**	Caribbean, Leeward Antilles, southwest coast of Curaçao, Boca Sint Michiel, Sun Reef	12°08.35'N, 68°59.89'W	7–21	19 Feb. 2014, 19:30–20:30, night	Reef flat and reef slope, above sand inside rock recesses, leg. K.J.W.	*Mysidium cubanense*
**17**	Caribbean, Leeward Antilles, southwest coast of Curaçao, Boca Sint Michiel, Sun Reef	12°08.35'N, 68°59.89'W	7–28	20 Feb. 2014, 08:15–09:00, day	Reef flat and reef slope, small swarms in entrance of small marine caves, also in empty shell of *Strombus* sp., leg. K.J.W.	*Anchialina typica typica*, *Mysidium antillarum*, *M. columbiae*, *M. integrum*, *M. triangulare*, *Parvimysis laminata*, *P. brattegardi*
**18**	Caribbean, Leeward Antilles, Curaçao, Playa Lagun	12°19.09'N, 69°09.07'W	5	28 Aug. 1997, day	Above sand inside small marine cave, leg. Peter Wirtz	*Mysidium cubanense*
**19**	Caribbean, Leeward Antilles, Curaçao, Playa Lagun	12°19.10'N, 69°09.12'W	5	28 Aug. 1997, day	Inside marine cave, leg. Peter Wirtz	*Parvimysis laminata Heteromysis bermudensis*
**20**	Caribbean, Greater Antilles, S-Jamaica, Portland Ridge / Parish Claredon, Jackson Bay Cave	17°44.11'N, 77°13.40'W	–	Mar. 1995, day	200 m from cave entrance, 4 psu, leg. C.D. Schubart, don. Wulf C. Kobusch	*Antromysis peckorum*
**21**	Bahamas, Long Island, ≈260 km to the southeast of the type locality, Stevens	this island at, 23°04.5'N, 75°01.2'W	45	9 Jan. 2005	Submarine cave, don. Kenneth Meland	*Palaumysis bahamensis*
**22**	Caribbean coast of Cuba, Isla de Pinos = Isla de la Juventud, Cueva de los Murciélagos (Bat Cave), sea distance 1500 m	this island at, 21°45.00'N, 82°49.20'W	–	23 Mar. 1973	Brackish water, leg. Christian Juberthie and Nicasio Viña (Cubano-Romanian Speleological Expedition)	*Antromysis juberthiei*
**23**	Caribbean coast of Cuba, Península de Zapata, El Brinco Cave	22°04.54'N, 81°03.37'W	–	29 Nov. 2014	Anchialine cave with entrance ≈390 m landwards Playa Girón, leg. Alejandro Martinez Garcia	*Palaumysis antillensis* sp. nov.
**24**	Caribbean coast of Cuba, Cueva Juanelo Piedra, brackish water lake in anchialine cave 5 km north of the Caribbean coast	22°43.80'N, 82°24.42'W	–	22 Nov. 1970	Leg. Traian Orghidan, Christian Juberthie, Nicasio Viña and Carlos Fundora (Cubano-Romanian Speleological Expedition)	*Antromysis cubanica*
**25**	Gulf of Mexico coast of Cuba, Playa La Habana; in front of Cuban Institute of Oceanology	estimated, 23°07.24'N, 82°26.61'W	26	22 Jan. 1968	From sponges in stands of corals, marine, leg. Mihai Băcescu	*Heteromysis spongicola*
**26**	Gulf of Mexico coast of Cuba, in front of Marine Research Center - Ciudad Habana	23°08.00'N, 82°20.00'W	13	13 May 1983	At the entrance of a small marine cave, leg. Manolo Ortiz, det. Mihai Băcescu	*Mysidium cubanense*
**27**	Mexico, Yucatán, 1.3 km S of Tecoh, Grutas de Tzab-Nah	20°43.83'N, 89°28.47'W	–	22 Apr. 1973	Anchialine cave; leg. J. Reddell & D. McKenzie	*Antromysis cenotensis*
**28**	Bermuda, Castle Grotto	32°21.22'N, 64°42.84'W	0.5	20 July 1982	Submarine cave, leg. T.M. Iliffe	*Platyops sterreri*
**29**	Bermuda, Walsingham Cave	32°20.91'N, 64°42.63'W	0.5	17 Feb. 1982	Leg. T.M. Iliffe	*Platyops sterreri*
**30**	Bermuda, Green Bay Cave	32°19.62'N, 64°44.39'W	15	31 Jan. 1984	Taken with hand net from bottom silt in the ‘Desert’, leg. T.M. Iliffe	*Bermudamysis speluncola*
**31**	Bermuda, Green Bay Cave	32°19.62'N, 64°44.39'W	15–18	28 Nov. 2000	Collected with plankton net from surface of silty sediments of Desert Room to the left of the Rat Trap, leg. T.M. Iliffe	*Bermudamysis speluncola*
**32**	Bermuda, Palm Cave	32°20.77'N, 64°42.76'W	16	13–16 Mar. 1982	Sampled with hand net from bottom silt in the Palm Cave Room, leg. T.M. Iliffe	*Bermudamysis speluncola*
**33**	Bermuda, Cherry Pit Cave	32°20.73'N, 64°42.64'W	8–12	12 Jan. 1984	Leg. T.M. Iliffe	*Bermudamysis speluncola*
**34**	Bermuda, Hamilton Parish, Deep Blue Cave	32°20.90'N, 64°42.69'W	6–15	1 Dec. 2000	Collected from surface of silty sediments and scraping of rock walls of main cavern, leg. T.M. Iliffe	*Bermudamysis speluncola*
**35**	Bermuda, Hamilton Parish, Grenadier Pool	32°21.16'N, 64°43.15'W	3–6	2 Dec. 2000	28 psu; mysids collected from water column of open pool and cavern, swimming in swarms in the water column, leg. T.M. Iliffe	*Bermudamysis speluncola*
**36**	Micronesia, Palau Archipelago, Koror Island, submarine Chandelier Cave	07°20.45'N, 134°27.04'E	10	8 Feb. 1985	Leg. T.M. Iliffe and D. Williams	*Palaumysis simonae*
**37**	Micronesia, Palau Archipelago, near Ngeruktabel Island, unnamed cave near Soft Coral Arch	07°16.04'N, 134°22.96'E	12	31 May 2002	Diver-operated hand net, leg. Sammy de Grave, don. Kenneth Meland	*Palaumysis simonae*
**38**	Micronesia, Palau Archipelago, unnamed island near Ngeruktabel Island, Ishura Cave	07°14.61'N, 134°22.07'E	7.5–2	24 Dec. 2001	Diver-operated hand net, leg. Tomoki Kase, don. Yukio Hanamura	*Palaumysis simonae*

### Collection materials

Collection materials originating from the Caribbean, Bermuda and Palau Archipelago (Stations 20–38) are included here for revisions of the genera *Palaumysis*, *Platyops*, and *Bermudamysis*. Type specimens of *Antromysis cubanica* Băcescu & Orghidan, 1971, *A. juberthiei* Băcescu & Orghidan, 1977, *Bermudamysis speluncola* Băcescu & Iliffe, 1986, *Heteromysoides spongicola* Băcescu, 1968, *Mysidium cubanense* Băcescu & Ortiz, 1984, *Palaumysis simonae* Băcescu & Iliffe, 1986, and *Platyops sterreri* Băcescu & Iliffe, 1986, were inspected without dissection upon visits at the Grigore Antipa National Museum of Natural History in Bucharest (**MINGA**) in 1998 and 2025. Six samples collected in 1982–1985 by Thomas M. Iliffe (Galveston) from anchialine caves in Bermuda were also studied in the collection of the MINGA. Three additional samples collected in 2000 were kindly provided by T.M. Iliffe. Important material was also obtained from Alejandro Martínez García (La Laguna), Yukio Hanamura (Yokohama), Wulf C. Kobusch (Bochum), and Kenneth Meland (Bergen).

### Repositories

**MINGA** Grigore Antipa National Museum of Natural History in Bucharest;

**MNHN** Muséum National d’Histoire Naturelle, Paris;

**NHMW** Natural History Museum of Vienna;

**ZMBN** Zoological Museum Bergen, Norway;

**ZMH** Museum of Nature Hamburg.

Holotypes of *Palaumysis antillensis* sp. nov. and *Bermudamysis caribbaea* sp. nov. are deposited at NHMW and the respective allotypes at MNHN. Paratypes of both species and non-types of several species are deposited at all above listed repositories, with some material retained for future studies. Holotype and allotype of *Heteromysis troglophila* sp. nov. were deposited at the NHMW and one paratype at the ZMH.

### Terminology

The species are categorized with some restrictions as troglobiont (stygobiont) and troglophile (stygophile), according to Wittmann (2023), mostly in agreement with Pesce et al. (1994): troglobionts have the cornea strongly reduced and dwell mostly in caves or groundwater. Troglophile species have mostly well-developed eyes (or nearly so) and are regularly but not exclusively found in these habitats. Trogloxenes are rarely found there and are here considered only if there is at least one record from such habitats in the Caribbean, irrespective of records elsewhere. Wilson (1989) defined ‘whip setae’ by the basal part bearing a thin flagellum separated by an articulation, suture, or at least by an optically dense section. Terminology of the alimentary tract according to Kobusch (1998). Larval stages distinguished essentially according to Wittmann (1981) as outlined in detail by Wittmann and Chevaldonné (2021). Other terminology in Wittmann and Chevaldonné (2021) and Wittmann (2024b). Nomenclature of the Mysidae at subfamily and tribe level after Wittmann et al. (2014).

### Additional abbreviations

**BL** body length in mm

**OB** Organ of Bellonci

**psu** practical salinity units

**TL** total length in mm

### Measurements, preparation, and microscopy

Body length was measured according to Tattersall and Tattersall (1951) from anterior margin of carapace (tip of rostrum) to terminus of telson without spines. Total length was measured according to Băcescu (from 1954 onwards) from tip of antennal scale to end of exopod of uropods excluding setae. TL is here indicated for comparison with the measurements of Băcescu. Eye length (mm) was measured along dorsal midline from insertion at the frons to the distalmost point, including the cornea. The size of the Organ of Bellonci (OB in µm) is the length of the mostly ellipsoidal, ovoid, or pyriform organ, or the diameter if spherical. Diameter of statoliths (µm) calculated as geometric mean of apparent length and width in dorsal view. Sexing was primarily based on checking for penes and traces of oostegites, while other secondary sexual characteristics were not always reliable (see below descriptions of *Palaumysis simonae* Băcescu & Iliffe, 1986, and *P. antillensis* sp. nov.). Preparation and microscopy were performed as detailed in Wittmann and Chevaldonné (2021).

### Molecular study

For some of the ‘PACOTILLES 2' material, DNA extraction was performed, followed by PCR amplification of gene regions commonly used in molecular systematics and phylogeography of mysids, mitochondrial cytochrome oxidase I (COI) and nuclear 18S ribosomal DNA (18S), all as in Chevaldonné et al. (2015). Amplicons were sequenced by Eurofins, Germany. Sequences were aligned and preliminarily analyzed in terms of % divergence. For *Mysidium*, a Neighbor Joining tree (NJ) analysis was performed on COI data using CLUSTALX 2.1 (Larkin et al. 2007). Bootstrapping support was assessed over 1000 replicates. Coding COI sequences were quality-checked by translation in amino acids to verify functioning genes.

## Biodiversity

Among the complete inventory of 27 cave-dwelling Mysidae species here acknowledged for the Caribbean (Table 2), five are estimated as stygobiont, 13 as troglophile (stygophile), and nine species as trogloxene.

**Table 2.** Inventory and estimates of troglophilia for Mysidae in marine and anchialine caves of the Caribbean.

No.	Subfamily	Tribe	Species	Troglophilia
1	Gastrosaccinae	Anchialinini	*Anchialina typica typica* (Krøyer, 1861)	trogloxene
2	Erythropinae	Erythropini	*Amathimysis sarbui* Băcescu, 1991	troglophile
3	Mysinae	Diamysini	*Antromysis cenotensis* Creaser, 1936	stygobiont
4			*Antromysis cubanica* Băcescu & Orghidan, 1971	stygobiont
5			*Antromysis juberthiei* Băcescu & Orghidan, 1977	stygobiont
6			*Antromysis peckorum* Bowman, 1977	stygobiont
7			*Parvimysis brattegardi* Wittmann, 2020	trogloxene
8			*Parvimysis laminata* Wittmann, 2020	troglophile
9	Mysinae	Anisomysini	Mysidium (Mysidium) cubanense Băcescu & Ortiz, 1984	trogloxene
10			Mysidium (Mysidium) gracile (Dana, 1852)	trogloxene
11			Mysidium (Mysidium) integrum W.M. Tattersall, 1951	trogloxene
12			Mysidium (Mysidium) triangulare Wittmann in Wittmann & Wirtz, 2019	troglophile
13			Mysidium (Orientomysidium) antillarum Wittmann in Wittmann & Wirtz, 2019	trogloxene
14			Mysidium (Orientomysidium) columbiae (Zimmer, 1915)	trogloxene
15			*Mysidium iliffei* Băcescu, 1991	troglophile
16	Palaumysinae	–	*Gironomysis lalanai* Ortiz, García-Debrás & Pérez, 1997	stygophile
17			*Palaumysis bahamensis* Pesce & Iliffe, 2002	troglophile
18			*Palaumysis antillensis* sp. nov.	troglophile + stygophile
19	Heteromysinae	Mysidetini	*Platyops sterreri* Băcescu & Iliffe, 1986	troglophile + stygophile
20			*Platyops stenoura* (Hanamura & Kase, 2004)	troglophile
21			*Bermudamysis caribbaea* sp. nov.	troglophile
22	Heteromysinae	Heteromysini	*Chelitrapezura dennisi* (Bowman, 1985), gen. nov. et comb. nov.	stygobiont
23			*Heteromysis spongicola* (Băcescu, 1968)	troglophile
24			Heteromysis (Heteromysis) cyanogoleus Bamber, 2000	troglophile
25			Heteromysis (Olivemysis) bermudensis G.O. Sars, 1885	trogloxene
26			Heteromysis (Olivemysis) floridensis Brattegard, 1969	trogloxene
27			*Heteromysis troglophila* sp. nov.	troglophile

*Heteromysis guitarti* Băcescu, 1968, reported by Băcescu and Iliffe (1986a) with some reservation as *H.* (*Olivaemysis*) aff. guitarti from sponges inside a marine cave of Bermuda is not included. All confirmed Caribbean records of *H. guitarti* are from sponges outside caves (Fukuoka 2005). Also not included is H. (Heteromysis) spottei Price & Heard, 2000, as there are no available Caribbean records from caves. This species not listed as cave-dwelling by Fukuoka (2005), nonetheless listed without details by Gerovasileiou et al. (2025) in the World Register of marine Cave Species (WoRCS).

### Remarks

Pesce et al. (1994) listed eight stygobiont and 15 stygophile (“submarine cave dwelling”) species of Mysidae at world-wide scale. One among the latter species (*Leptomysis buergii* Băcescu, 1966) is considered trogloxene by Wittmann (2023). Without differentiation for categories of troglophilia, Gerovasileiou et al. (2025) listed 38 species plus one subspecies at world-wide scale (including *H. spottei*, see preceding paragraph). However, this list appears quite incomplete by not taking account of five species already listed by Pesce et al. (1994). Merging the lists of Pesce et al. (1994) and Gerovasileiou et al. (2025) results in 43 species (not counting *H. spottei*). The present list of 27 species from the Caribbean adds 17 species (Table 2: nos 1, 2, 7–16, 18, 20–22, 27) to the merged list, yielding a total of 60 cave-dwelling Mysidae species. This total appears moderately underestimated, but nonetheless corroborates the eminent contribution of the Caribbean to the world-wide biodiversity in aquatic cave environments. A revised world list is currently in preparation.

## Systematics

**Order Mysida Boas, 1883**

**Family Mysidae Haworth, 1825**

**Subfamily Gastrosaccinae Norman, 1892**

**Tribe Anchialinini Wittmann, Ariani & Lagardère, 2014**

**Genus *Anchialina* Norman & Scott, 1906**

### Anchialina typica typica

Taxon classificationAnimaliaMysidaMysidae

(Krøyer, 1861)

7199468F-0D49-5F5E-BCFF-64CBE322B329

Fig. 2A–D

Anchialus typicus Krøyer, 1861: 53–59, fig. 7a–l in pl. 2 (original description, tropical Atlantic).
Anchialina typica : Norman and Scott 1906: 24 (replacement name Anchialina for preoccupied genus name Anchialus); Ii 1964: 188–195, figs 48, 49 (records, description); Brattegard 1970a: 24–28, fig. 6 (description, Bahamas); Meland et al. 2015: 23 (biogeography).
Anchialina typica typica : Nouvel 1971: 327, fig. 1 (diagnosis, Atlantic distribution); Fukuoka and Murano 2002: 100 (in key); Wittmann et al. 2009: 144 (records from Canary and Selvagens islands).
Anchialina tipica : Băcescu 1991: 6 (misspelling of species name, record, Bahamas).

#### Material examined.

Leeward Antilles — **Curaçao** • 1 imm. (BL = 1.5 mm) during daytime extracted from a large empty shell of *Strombus* sp. at Sun Reef (Station 17).

#### Short diagnosis at species level.

*Anchialina* Norman & Scott, 1906, with trapezoid rostrum with concave terminal margin, rostrum covering base of eyestalks; antennal scale short, not extending beyond median segment of antennular trunk; eyes normal-sized, cornea calotte-shaped, in dorsal view weakly bean-shaped; pleomere 1 of female and pleomeres 1–5 of male with well-developed pleural plates; pleopods with unilobed pseudobranchiae; exopod of male pleopod 3 with 11–13 segments, distal segment with three setae; exopod of uropod with 16–20 spines along distal 65–75%; telson trapezoid with short apical cleft, distal half of each lateral margin with dense series of 25–35 spines; distolateral edges of telson each with large spine mesially accompanied by a half as long spine; cleft 1/6 telson length, its margin completely armed with laminae.

#### Differential diagnosis at subspecies level.

**Figure 2.**
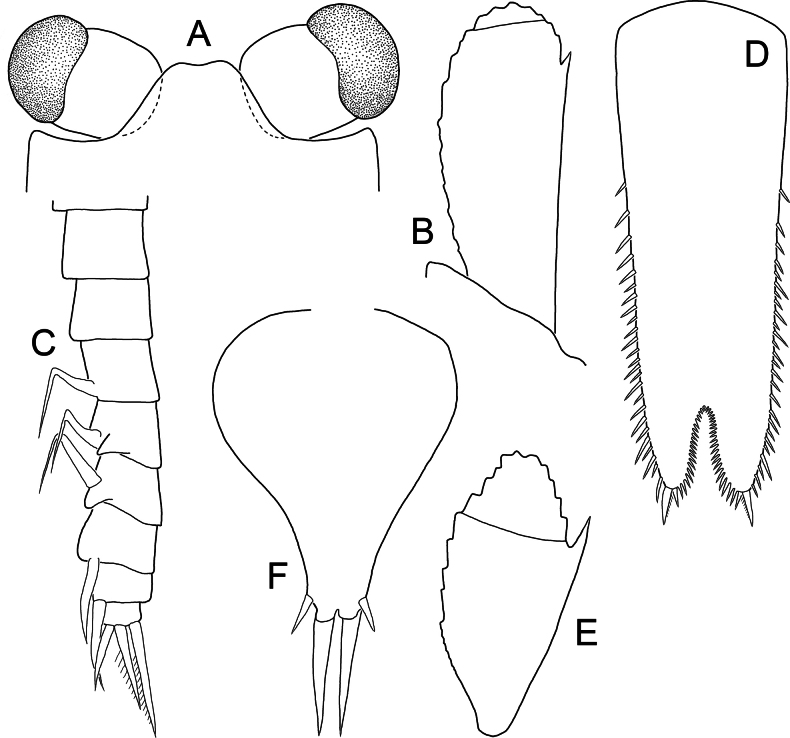
Selected diagnostic characters of **A–D**. *Anchialina typica typica* (Krøyer); **E, F**. *Amathimysis sarbui* Băcescu. **A**. Eyes and anterior margin of carapace; **B, E**. Antennal scale, setae omitted; **C**. Distal half of exopod of male pleopod 3, only modified setae shown; **D, F**. Telson. Modified after Ii 1964 (**A, B, D**), Nouvel 1971 (**C**) and Băcescu 1991 (**E, F**).

The nominotypical Atlantic-Mediterranean subspecies *A. typica typica* (Krøyer, 1861) differs from the Indian Ocean and Pacific *A. typica orientalis* H. Nouvel, 1971, by exopod of male pleopod 3 (Fig. 2C) with modified bent setae at fourth to sixth segments from tip and by disto-lateral angle of third segment from tip not clearly projecting laterally vs modified bent setae at fifth to seventh or eighth segments, and disto-lateral angle of third segment strongly projecting laterally.

#### Distribution

**Figure 3.**
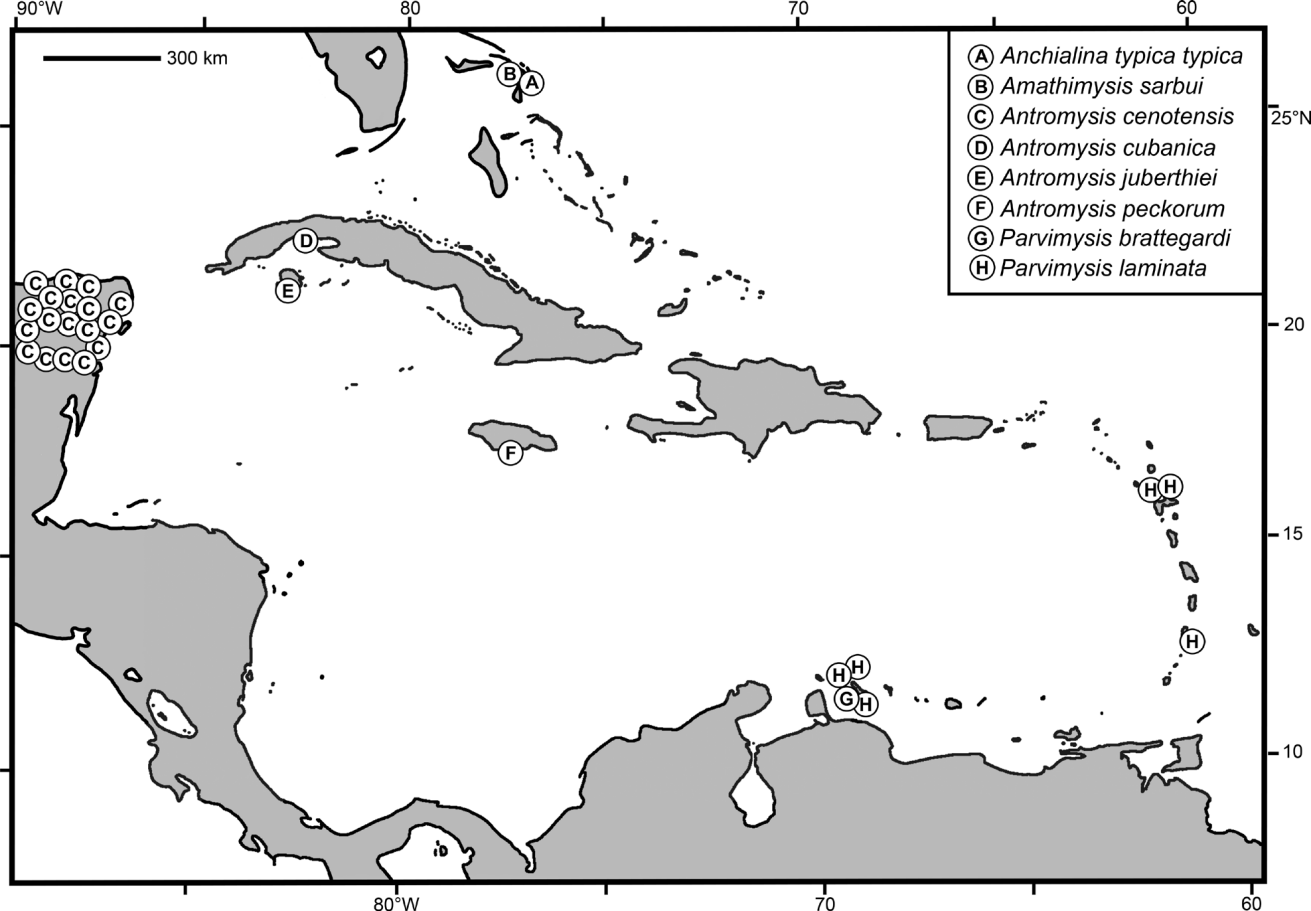
Distribution of the species of **A**. *Anchialina* Norman & Scott; **B**. *Amathimysis* Brattegard; **C–F**. *Antromysis* Creaser; **G, H**. *Parvimysis* Brattegard, in marine and anchialine caves of the Caribbean and adjacent areas. Coinciding points slightly displaced.

(Fig. 3A). This species shows an essentially epipelagic, nearly circumtropical distribution (Meland et al. 2015). The subspecies *A. typica typica* occurs in the western and eastern Atlantic and in the Mediterranean Sea (Wittmann et al. 2009). Only one ♀ was reported by Băcescu (1991) from a submarine cave at the Abaco Islands in the Bahamas (ca 26°21'N, 77°09'W). The present record from the interior of a large gastropod shell at Curaçao (Station 17) confirms a somewhat sciaphilic habitat preference. Nonetheless, this species is considered clearly trogloxene due to the great dominance of pelagic records.

##### Subfamily Erythropinae Hansen, 1910

**Tribe Erythropini Hansen, 1910**

**Genus *Amathimysis* Brattegard, 1969**

### Amathimysis sarbui

Taxon classificationAnimaliaMysidaMysidae

Băcescu, 1991

6CBEA459-0CE4-529E-8819-5D917325A862

Fig. 2E, F

Amathimysis sarbui Băcescu, 1991: 6–8, fig. 2 except for the erroneous legend (original description); Wittmann 2024a: 145 (in key).
Amathimysis illifei Băcescu, 1991: legend of fig. 2 (nomen nudum, species name confused with that of Mysidium iliffei Băcescu, 1991, described in the same publication).

#### Note.

Only the ♀ holotype known. Băcescu (1991) claimed deposition of slides with registry number 247 at the MINGA. However, there is neither a respective entry in the inventory booklet nor such material in the type collection (Iorgu Petrescu, pers. comm. in 2025).

#### Short diagnosis.

Based on adult female holotype: *Amathimysis* Brattegard, 1969, with very large eyes, larger than the small antennal scale; antennal scale stout, only twice as long as maximum width, terminal segment 1/4–1/3 of total scale length, lateral margin with one tooth marking end of bare portion; endopod of uropods without spine; telson subtriangular, roughly heart-shaped, terminal margin narrowly truncate with two densely set large spines, each lateral margin with only one small subterminal spine.

#### Distribution

(Fig. 3B). Băcescu (1991) described the holotype from a submarine cave at the Abaco Islands in the Bahamas (ca 26°21'N, 77°09'W). Unless additional records become available, the very large eyes point to a troglophile rather than troglobiont status.

##### Subfamily Mysinae Haworth, 1825

**Tribe Diamysini Wittmann, Ariani & Lagardère, 2014**

### Genus Antromysis

Taxon classificationAnimaliaMysidaMysidae

Creaser, 1936

23FB3B33-94A5-5C4B-AE2E-A4D28F2B2E32

Figs 4, 5

Antromysis Creaser, 1936: 121 (original description from cave in Yucatán); W.M. Tattersall 1951: 229, 230 (revised diagnosis); Mauchline 1980: 33, fig. 8.26 (in key to genera); Henderson and Bamber 1983: 143 (removal of invalid subgenus Parvimysis); Bamber and Henderson 1990: 398 (removal of invalid subgenus Surinamysis); Pesce et al. 1994: 115 (in list of hypogean species); Fukuoka 2006: fig. 1 (distribution); Price 2020: 974, 975 (in key).
Antromysis (Antromysis) : Bowman 1977: 36, 37 (correct but meanwhile obsolete subgeneric assignment); Bowman 1982: 201.
nec Antromysis (Anophelina): Bowman 1977: 36, 37 (synonymous subgenus); Bowman 1982: 201.
nec Antromysis (Surinamysis)Bowman 1977: 37 (outdated combination); Bowman 1980: 208; Bowman 1981a: 27; Bowman 1982: 201.
nec Antromysis (Parvimysis): Bowman 1977: 37 (invalid combination); Bowman 1981a: 27; Bowman 1982: 201.
nec Antromysis: Mauchline 1980: 36, fig. 10.5 (invalid assignment of Parvimysis species in key to genera).

#### Short diagnosis.

Diamysini with separate subovate eyestalks, no cornea, no pigment (except for *Antromysis anophelinae* W.M. Tattersall, 1951, showing fused eyestalks, distally with pigmented band of rudimentary ommatidia); antennular trunk without accessory flagellum; antennal scale setose except for a short bare basal portion of the lateral margin; thoracic endopods 3–7 with 2- or 3-segmented carpopropodus; female pleopods forming a setose, uniramous, unsegmented bilobate plate; male pleopods biramous, endopod also forming an unsegmented bilobate plate, exopod 2- or 3-segmented, apically with a single strong seta; uropods without spine; telson small, stout, length <1.3× maximum width, lateral margins each with 0–4 spines.

**Figure 4.**
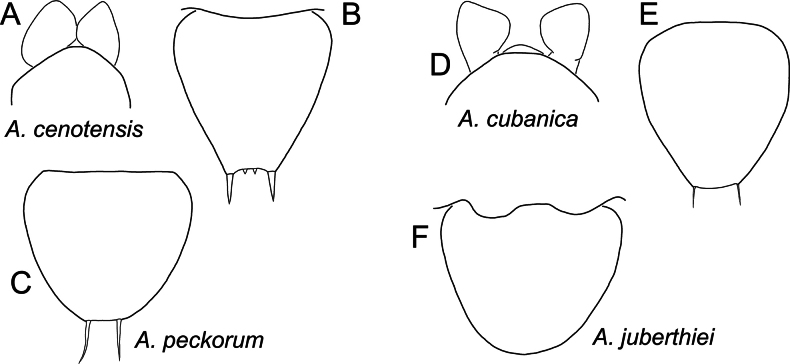
Differentiation between the species of *Antromysis* Creaser, so far recorded in Caribbean caves. **A, B**. Eyes with frontal margin of carapace (**A**) and telson (**B**) in *Antromysis cenotensis* Creaser; **C**. Telson of *A. peckorum* Bowman; **D, E**. Eyes with frontal margin of carapace (**D**) and telson (**E**) in *A. cubanica* Băcescu & Orghidan; **F**. Telson in *A. juberthiei* Băcescu & Orghidan. Modified after Bowman 1977 (**A–C**), Băcescu and Orghidan 1971 (**D, E**) and Băcescu and Orghidan 1977 (**F**).

**Figure 5.**
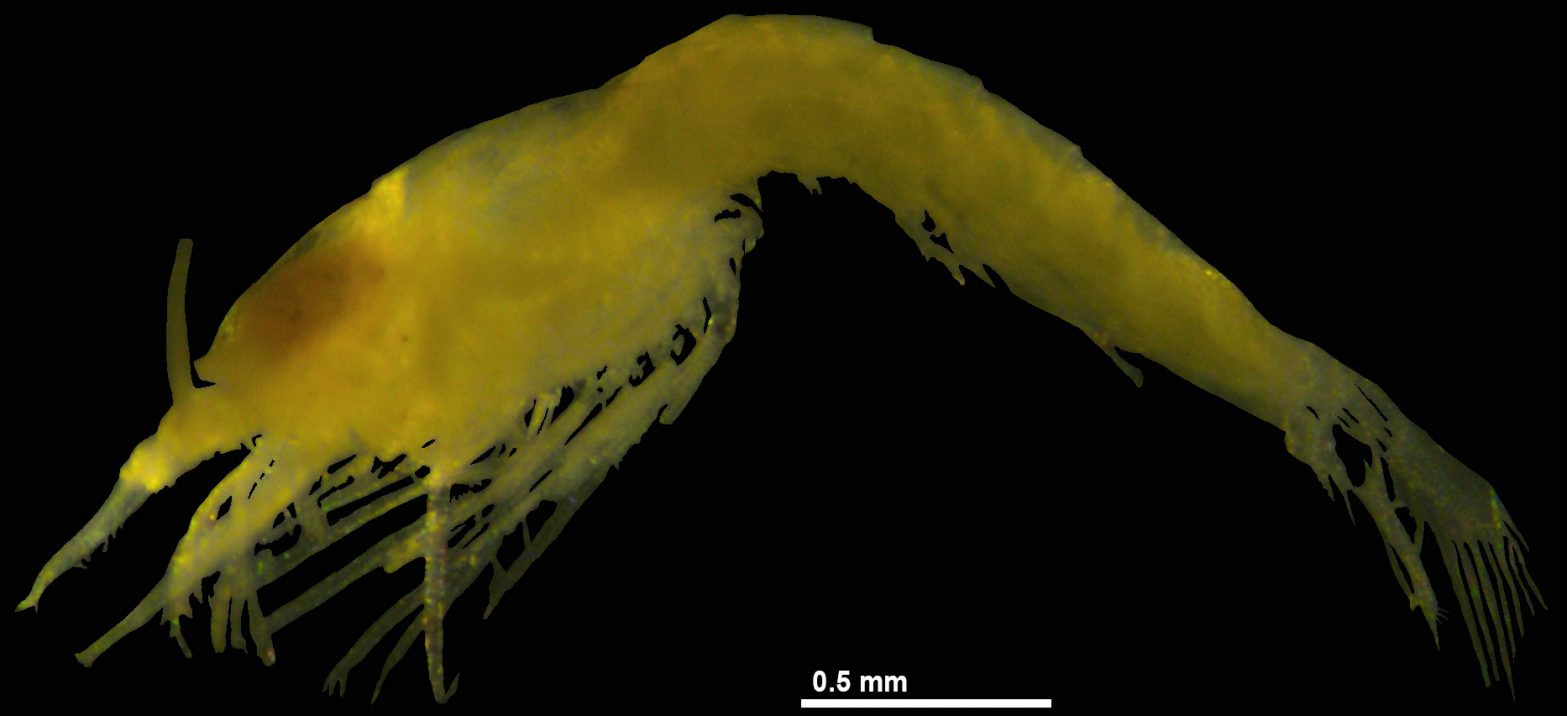
*Antromysis cubanica* Băcescu & Orghidan, holotype ♂ from the anchialine Cueva Juanelo Piedra at the Caribbean coast of Cuba.

#### Species inventory.

Six species are acknowledged at a world-wide scale; bold italics highlight species recorded from Caribbean caves: ***Antromysis cenotensis*** Creaser, 1936 (type species), *A. anophelinae* W.M. Tattersall, 1951, ***A. cubanica*** Băcescu & Orghidan, 1971, ***A. juberthiei*** Băcescu & Orghidan, 1977, ***A. peckorum*** Bowman, 1977, and *A. reddelli* Bowman, 1977. See also key to Caribbean species below.

### Antromysis cenotensis

Taxon classificationAnimaliaMysidaMysidae

Creaser, 1936

B1C7E09B-8BA2-5C67-8255-232EFB4ADD2F

Fig. 4A, B

**Figure 6.**
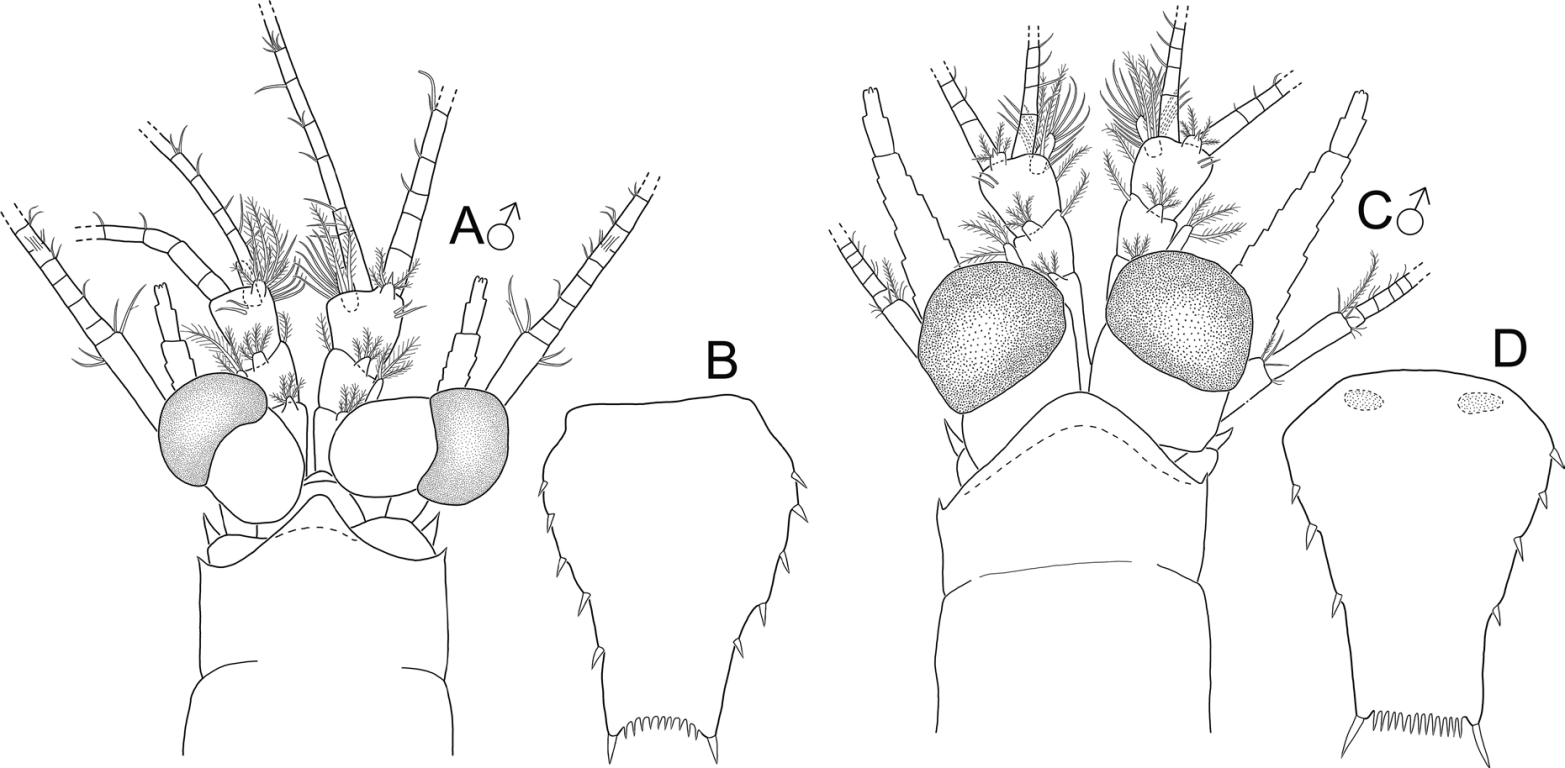
Differentiation between the species of *Parvimysis* Brattegard, from Caribbean caves. **A, B**. Cephalic region (**A**) and telson (**B**) in *P. brattegardi* Wittmann; **C, D**. Cephalic region (**C**) and telson (**D**) in *P. laminata* Wittmann. Slightly modified after Wittmann 2020.

Antromysis cenotensis Creaser, 1936: 121–123, figs 13–24 (original description, Balamcanché Cave in Yucatán); Bowman 1977: figs 1–4 (records, supplemental description); Reddell 1981: 105, Table 2, fig. 13 (records in Yucatán); Pesce et al. 1994: 115 (stygobiont status, endemic to Yucatán); Wittmann and Ariani 2019: fig. 2, suppl. (vaterite statoliths, biogeography); Price 2020: 978, figs 23.181C, 23.183F (in key, neotropical fauna); Daneliya and Wittmann 2021: table 10.1 (endangerment status); Liévano-Beltrán and Simões 2021: 1–13, Table 1, figs 1–6 (biogeography, records, bioindicator); Mejía-Ortíz et al. 2022: tables 1, 3 (freshwater subterranean species, vulnerability of subterranean waters); Wittmann 2023: table SS1 (sensory organs).
Antromysis (Antromysis) cenotensis : Bowman 1977: 27–31; Reddell 1981: 103; Iliffe 1992b: 201, 202 (in checklist, distribution); Pesce and Iliffe 2002: 275, 276, figs 14–21 (record, morphological variability); Chávez-Solís et al. 2018: 2, 4 (micro-detritivorous, prey of decapods).
Antromysis coenotensis : Băcescu and Orghidan 1971: 228 (misspelling in key).

#### Material examined.

Yucatán • 5 ♀♀ ad. (BL = 2.6–3.0 mm), 4 ♂♂ ad. (BL also 2.6–3.0 mm) from anchialine cave in the Grutas de Tzab-Nah (Station 27).

#### Short diagnosis.

*Antromysis* with eyestalks closely set together; exopod of male pleopod 3 two-segmented, basal segment without seta; telson slightly longer than maximum width near basis, with bare, convex lateral margins; telson terminally truncate, terminal margin slightly concave, terminal width 0.3–0.5× maximum width; each disto-lateral edge with a comparatively strong spine; the pair of disto-lateral spines flanking 1–3 (mostly 2) small spines in median or paramedian position.

#### Distribution

(Fig. 3C). In freshwater and anchialine cenotes of Quintana Roo and the northern Yucatán Peninsula, including Caribbean and Gulf of Mexico coasts, 18–21°N, 87–90°W, 1–37 m depth (Bowman 1977; Reddell 1981; Pesce and Iliffe 2002; Liévano-Beltrán and Simões 2021).

### Antromysis cubanica

Taxon classificationAnimaliaMysidaMysidae

Băcescu & Orghidan, 1971

02078E7A-7A62-5EE6-99FC-8320465AC618

Figs 4D, 4E, 5

Antromysis cubanica Băcescu & Orghidan, 1971: 226–228, fig. 1 (original description, Cuba, anchialine cave); Pesce et al. 1994: 115 (stygobiont status, endemic to Cuba); Petrescu and Wittmann 2009: 62 (types in museum collection); Ortiz and Lalana 2018: 74, fig. 16B (Cuban archipelago, in key); Price 2020: 979, figs 23.181F, 23.183G (in key, neotropical fauna); Daneliya and Wittmann 2021: table 10.1 (endangerment status); Wittmann 2023: table SS1 (sensory organs).

#### Types examined.

Cuba • ***holotype***, ♂ ad. (BL = 3.0 mm; MINGA 49070) from the anchialine Cueva Juanelo Piedra (Station 24) • ***paratype***, ♀ with 4 eggs (BL = 3.3 mm; MINGA MYS 455), sampling data as for holotype.

#### Short diagnosis.

Eyestalks set widely apart; exopod of male pleopod 3 two-segmented, basal segment without seta; telson slightly longer than maximum width near basis, with bare, convex lateral margins; telson with transverse, weakly convex terminal margin flanked by a pair of thin spines at distolateral edge, no additional spines.

#### Notes on male holotype

(Fig. 5). Distal segment of antennular trunk ventrally with small brush of setae, probably homologous with the appendix masculina otherwise not detected there. Eyes dorsoventrally flattened by a factor of 2.0, no cornea, no pigment in the ethanol-preserved specimen. Eyes with single-plane dorsal face and weakly convex ventral face. Anterior 1/5 of eye obliquely truncate in the transversal plane (i.e. in lateral view). Penes stout, length 6% BL, telson length 8% BL.

#### Distribution

(Fig. 3D). At species level so far reported only from a brackish-water lake in the anchialine Cueva Juanelo Piedra at the Caribbean coast of Cuba (Station 24: type locality). It cannot be excluded that “*Antromysis*” recorded by Bolívar y Pieltain (1943) from the Cueva de Quíntanal in the municipality of Alquízar in Cuba belongs to this species (locality details not found).

### Antromysis juberthiei

Taxon classificationAnimaliaMysidaMysidae

Băcescu & Orghidan, 1977

08350BBE-0092-5F58-939E-34EB723A3D6E

Fig. 4F

Antromysis juberthiei Băcescu & Orghidan, 1977: 263–265, fig. 1 (original description); Pesce et al. 1994: 115 (stygobiont status, endemic to Cuba); Petrescu and Wittmann 2009: 62 (types in museum collection); Ortiz and Lalana 2018: 74, fig. 16A (Cuban archipelago, in key); Price 2020: 979, fig. 23.181E (in key, neotropical fauna); Daneliya and Wittmann 2021: table 10.1 (endangerment status).

#### Type examined.

Cuba • ***holotype***, ♀ ad. (BL = 3.8 mm; MINGA 49161) from the Cueva de los Murciélagos (= Bat Cave; Station 22).

#### Short diagnosis.

*Antromysis* with eyestalks set well apart; exopod of male pleopod 3 two-segmented, basal segment distally with comparatively large barbed seta; telson slightly shorter than maximum width near basis; telson terminally broadly rounded, all around with bare margin.

#### Distribution

(Fig. 3E). So far known only from brackish waters in the Cueva de los Murciélagos (Station 22: type locality) 1500 m landwards from the coast of Isla de Pinos, also called Isla de Juvendud; this island off the Caribbean coast of Cuba.

### Antromysis peckorum

Taxon classificationAnimaliaMysidaMysidae

Bowman, 1977

52DE87ED-7BB2-57BA-BEB5-5C2EF8A8C9AB

Fig. 4C

Antromysis peckorum Bowman, 1977: 31–33, figs 5, 6 (original description); Pesce et al. 1994: 116 (stygobiont status, endemic to Jamaica); Wittmann and Ariani 2019: fig. 2, suppl. (vaterite statoliths, biogeography); Price 2020: 979, fig. 23.181D (in key, neotropical fauna); Wittmann 2023: table SS1 (sensory organs).

#### Material examined.

Jamaica • 2 ♀♀ subad. (BL = 2.4–2.8 mm, ZMH-K-066768) from Jackson Bay Cave (Station 20) • 2 ♀♀ ad. (BL = 2.7–2.9 mm, NHMW-ZOO-CR-31423), sampling data as for preceding.

#### Short diagnosis.

*Antromysis* with eyestalks closely set together; exopod of male pleopod 3 two-segmented, basal segment without seta; telson slightly shorter than maximum width near basis; telson with bare, convex lateral margins; telson terminally truncate, terminal width 0.2× maximum width; distolateral corners with pair of long slender spines flanking 0–2 (mostly 0) (para)median spines.

#### Distribution

(Fig. 3F). So far known only from the system of the anchialine Jackson Bay Cave (type locality) at the southern coast of Jamaica (Bowman 1977). This also includes the present new records from Station 20.

### Genus Parvimysis

Taxon classificationAnimaliaMysidaMysidae

Brattegard, 1969

41220AED-E5CF-5B3A-9CB8-8A893D01DC76

Fig. 6

Parvimysis Brattegard, 1969: 74, table 5 (diagnosis); Bowman 1977: 37 (downgrading to subgenus level, in key); Henderson and Bamber 1983: 143 (retaining genus level); Nouvel et al. 1999: 79 (in list of genera); Wittmann 2018: 542, 543 (diagnosis, in key, vaterite statoliths); Price 2020: 974, 979, 980 (in key to genera, key to species); Wittmann 2020: 3, 4 (revision, species inventory, Caribbean, West Atlantic, key to species).
Antromysis (Parvimysis) : Bowman 1977: 37; Bowman 1981a: Table 1 (in list); Modlin 1987a: 110 (species record); Camp et al. 1998: 126 (in checklist).
Antromysis : Müller 1993: 209, 210 (listed Parvimysis in synonymy); Vázquez-Bader 2000: 48 (attributed the type species Parvimysis bahamensis Brattegard, 1969, to Antromysis).

#### Short diagnosis.

Diamysini with eyes well-developed, cornea well pigmented; antennular trunk terminally with the usual two flagella in both sexes, no accessory flagellum; antennal scale setose all around, with small terminal segment; thoracic endopods 1–8 with unsegmented carpus and propodus; female pleopods 1–5 and male pleopods 1–3, 5 representing simple setose unsegmented rods; male pleopod 4 biramous, endopod forming an unsegmented bilobate plate, exopod 2- to 3-segmented, apical segment with a single strong seta, in certain species accompanied by a small seta, remaining segments without seta; uropods without spine; telson small, subtriangular to trapezoid, terminally truncate to distinctly emarginated; lateral margins with spines, latero-terminal edges each with one larger spine; terminal margin bare or armed with diverse cuticle structures.

#### Species inventory.

Fourteen species are acknowledged; bold italics highlight two species recorded from Caribbean caves: *Parvimysis bahamensis* Brattegard, 1969 (type species), *P. almyra* Brattegard, 1977, *P. pisciscibus* Henderson & Bamber, 1983, *P. amazonica* Wittmann, 2018, *P. fittkaui* Wittmann, 2018, *P. fluviatilis* Wittmann, 2018, *P. lacustris* Wittmann, 2018, *P. macrops* Wittmann, 2018, *P. tridens* Wittmann, 2018, ***P. brattegardi*** Wittmann, 2020, ***P. laminata*** Wittmann, 2020, *P. nuda* Wittmann, 2020, *P. ornata* Wittmann, 2020, and *P. pricei* Wittmann, 2020. See also key below to species from Caribbean caves.

### Parvimysis brattegardi

Taxon classificationAnimaliaMysidaMysidae

Wittmann, 2020

C065A6E0-DDEE-5B1B-9B6A-FA4F13B2904E

Fig. 6A, B

**Figure 7.**
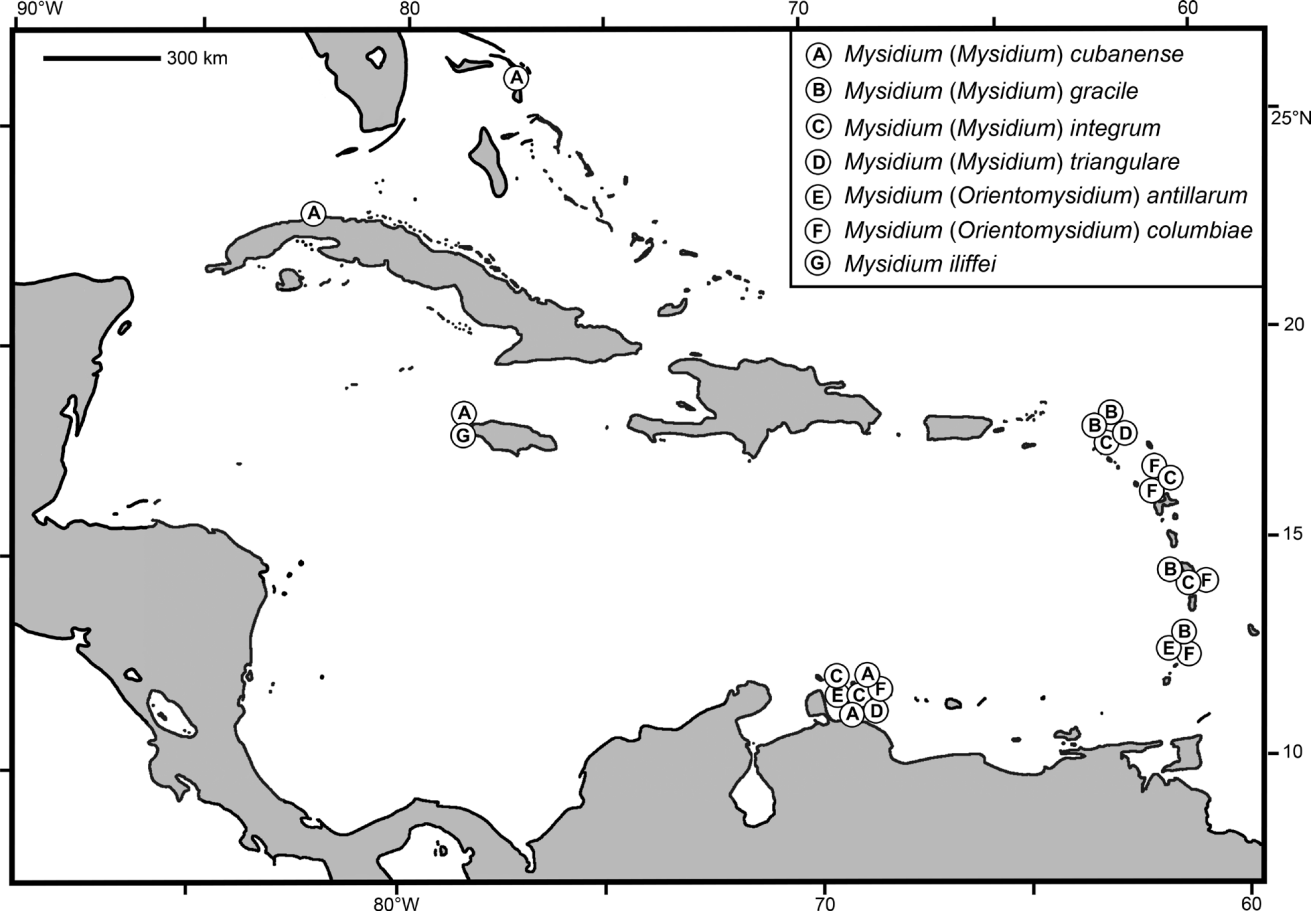
Distribution of the species of *Mysidium* Dana, in marine caves of the Caribbean and adjacent areas. Coinciding points slightly displaced.

Parvimysis brattegardi Wittmann, 2020: 14–18, tables 1, 2, figs 6, 7 (original description, Curaçao); Wittmann 2023: table SS1 (sensory organs, trogloxene status).

#### Material examined.

Leeward Antilles — **Curaçao** • 22 ♀♀ ad. (BL = 2.5–3.5 mm), 44 ♂♂ ad. (BL = 1.8–2.5 mm), 21 subad., 10 imm., 6 juv. from swarms in entrance of two small caves (Station 17).

#### Short diagnosis.

*Parvimysis* with subtriangular, apically rounded rostrum; antennal scale with basal segment not reaching to end of antennular trunk; eyes moderately sized, maximum cornea diameter 1.5–1.7× length of terminal segment of antennular trunk in dorsal view; exopod of male pleopod 4 three-segmented, ending in large modified seta plus a minute lobe bearing a minute seta; telson trapezoid to subtriangular with weakly concave terminal margin lined by 4–12 laminae that are 0.2–0.5× length of latero-apical spines.

#### Distribution

(Fig. 3G). In sublittoral marine waters of Curaçao, 12°N, 69°W, depth 1–32 m, swarms on reef flat and slope, among algae, in rock recesses and small caves (Wittmann 2020, 2023).

### Parvimysis laminata

Taxon classificationAnimaliaMysidaMysidae

Wittmann, 2020

0B7A6255-0BF7-5FD9-9762-F18D41145071

Fig. 6C, D

Parvimysis sp. B: Wittmann and Ariani 2019: fig. 1E, F, suppl. (vaterite statoliths, biogeography).
Parvimysis laminata Wittmann, 2020: 10–14, tables 1, 2, figs 3–5 (original description, Curaçao); Wittmann 2023: table SS1 (sensory organs, trogloxene status).

#### Material examined.

Lesser Antilles — **SW-Bequia** • 4 ♀♀ ad. (ZMH-K-066766) from Full Moon Cave (Station 5) • 3 ♀♀ ad., 1 ♂ ad. (NHMW-ZOO-CR-31414), sampling data as for preceding • 2 ♀♀ ad., 2 ♂♂ ad. (MNHN-IU-2025-2839), sampling data as for preceding — **Guadeloupe** • 9 ♀♀ ad. (BL = 2.6–2.9 mm), 13 ♂♂ ad. (BL = 1.7–2.4 mm) (MINGA MYS 458) from Cathedral Cave (Station 9) • 3 ♀♀ ad. (BL = 2.4–2.8 mm), 3 ♂♂ ad. (BL = 1.9–2.3 mm) (MNHN-IU-2025-2840) from Barracuda Cave (Station 11). Leeward Antilles — **Curaçao** • 10 ♀♀ ad. (BL = 2.7–3.0 mm), 10 ♂♂ ad. (BL = 2.1–2.5 mm) (ZMBN 135401) inside marine cave at Playa Lagun (Station 19 — type locality) • 20 ♀♀ ad., 14 ♂♂ ad. (ZMH-K-066774) from entrance of small caves at Sun Reef (Station 17) • 15 ♂♂ ad., 17 ♀♀ ad., 2 imm. (MNHN-IU-2025-2838), sampling data as for preceding.

#### Short diagnosis.

*Parvimysis* with short, broadly rounded rostrum; antennal scale with basal segment extending beyond antennular trunk; eyes very large, maximum cornea diameter 1.8–2.4× length of terminal segment of antennular trunk in dorsal view; exopod of male pleopod 4 three-segmented, ending in large modified seta plus a minute lobe bearing a minute seta; telson trapezoid, terminally truncate or slightly concave, its terminal margin lined by 9–16 laminae that are 0.4–0.8× length of latero-apical spines.

#### Distribution and habitat

(Fig. 3H). In euhaline coastal waters of the Leeward Antilles (Curaçao and Bonaire: Stations 15, 17, 19) and the Lesser Antilles (Bequia and Guadeloupe: Stations 5, 9, 11), total range 12–16°N, 61–69°W, 1–32 m depth. Recorded from the open sea floor where it hovers in swarms and loose aggregations a few cm above the substrate, also found over and inside rock recesses (Wittmann 2020). Present records from the semi-dark zone of diveable caves (Stations 5, 9, 11). The mysids appeared colorless *in situ*. In the Barracuda Cave (Station 11) they were found close to the reddish *Bermudamysis caribbaea* sp. nov. Classified as trogloxene by Wittmann (2023), but inclusion of the new records points rather to a troglophile status.

### Tribe Anisomysini Wittmann, Ariani & Lagardère, 2014

#### Genus Mysidium

Taxon classificationAnimaliaMysidaMysidae

Dana, 1852

A888E979-599C-55CE-833B-3E8D1224C2B1

Figs 7, 8, 9

Mysidia Dana, 1850: 130 (new genus, diagnosis; junior homonym of the hemipteran genus Mysidia Westwood, 1840); Zimmer 1915b: 211, 215 (invalid restoration of the homonym Mysidia Dana, 1850).
Macromysis : Dana 1852: 638, 653 (diagnosis; incorrect assignment to Macromysis White, 1847, in turn a replacement name of the homonym Themisto Goodsir, 1842, and simultaneously a junior synonym of Praunus Leach, 1814); Meland et al. 2015: Table 1 (classification history).
Mysidium Dana, 1852: 638, 653 (replacement name Mysidium proposed in footnotes for occupied Mysidia); W.M. Tattersall 1951: 222 (Mysidium acknowledged); Wittmann and Wirtz 2019: 3–6 (revision, diagnosis).

##### Diagnosis.

Shortened after Wittmann and Wirtz (2019): Anisomysini with well-developed, normal eyes; antennular trunk normal, without accessory flagellum; antennal scale setose all around, with small terminal segment; thoracopods 3–8 normal, endopods with 2- or 3-segmented carpopropodus; pleopods 1–5 of females and pleopods 1–3, 5 of males reduced to vestigial, setose, unsegmented rods; male pleopod 4 with distinct sympod, unsegmented endopod and 3- or 4-segmented, slender exopod; terminal two segments of exopod each bearing a large modified seta; uropods normal, setose all around, without spines; telson longer than broad, with entire or incised terminal margin, with spines and laminae on distal half; terminal margin with continuous series of densely-set spines and laminae.

##### Species inventory.

Eleven species are acknowledged at world-wide scale; bold italics highlight seven species recorded from Caribbean caves: ***Mysidium*** (***Mysidium***) **gracile** (Dana, 1852) (type species), ***M.*** (***M.***) **integrum** W. M. Tattersall, 1951, ***M.*** (***M.***) **cubanense** Băcescu & Ortiz, 1984, M. (M.) rubroculatum Băcescu & Ortiz, 1984, M. (M.) rickettsi Harrison & Bowman, 1987, ***M.*** (***M.***) **triangulare** Wittmann in Wittmann & Wirtz, 2019, M. (M.) winfieldi Hendrickx, Hernández-Payán & Gómez-Gutierrez, 2023, M. (Occimysidium) pumae Ortiz, Hendrickx & Winfield, 2017, ***M.*** (***Orientomysidium***) ***columbiae*** (Zimmer, 1915), ***M.*** (***O.***) ***antillarum*** Wittmann in Wittmann & Wirtz, 2019, and ***M. iliffei*** Băcescu, 1991. See also below key to species from Caribbean caves.

##### DNA data

**Figure 8.**
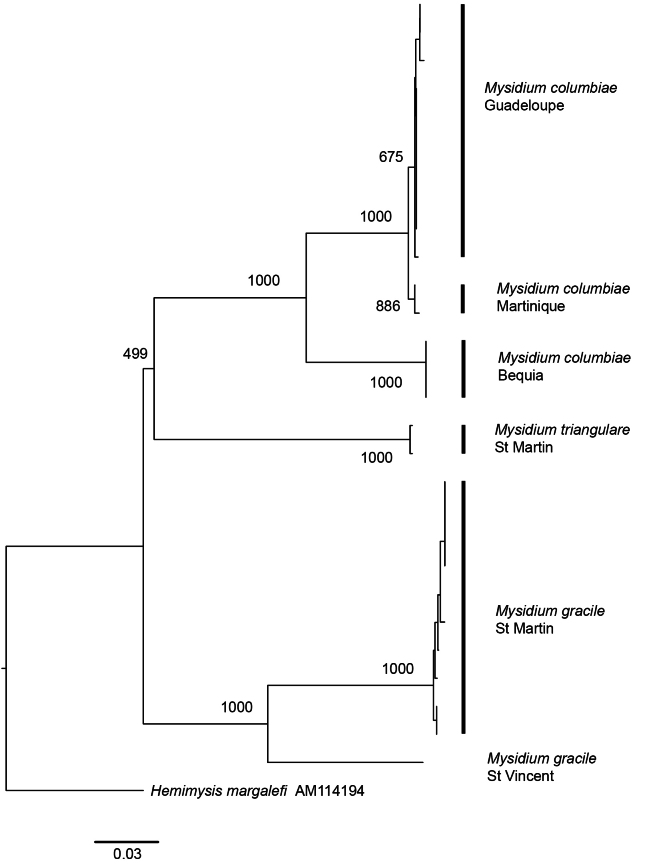
Distance tree (neighbor-joining) of mitochondrial COI sequences of *Mysidium* specimens collected in the Lesser Antilles, rooted with *Hemimysis margalefi* Alcaraz, Riera & Gili. Bootstrap values (1000 replicates) are shown at nodes.

(Fig. 8). Sequences of mitochondrial COI were obtained for three species of *Mysidium*. No other COI sequences are available in GenBank of any *Mysidium* or closely related genera.

**Figure 9.**
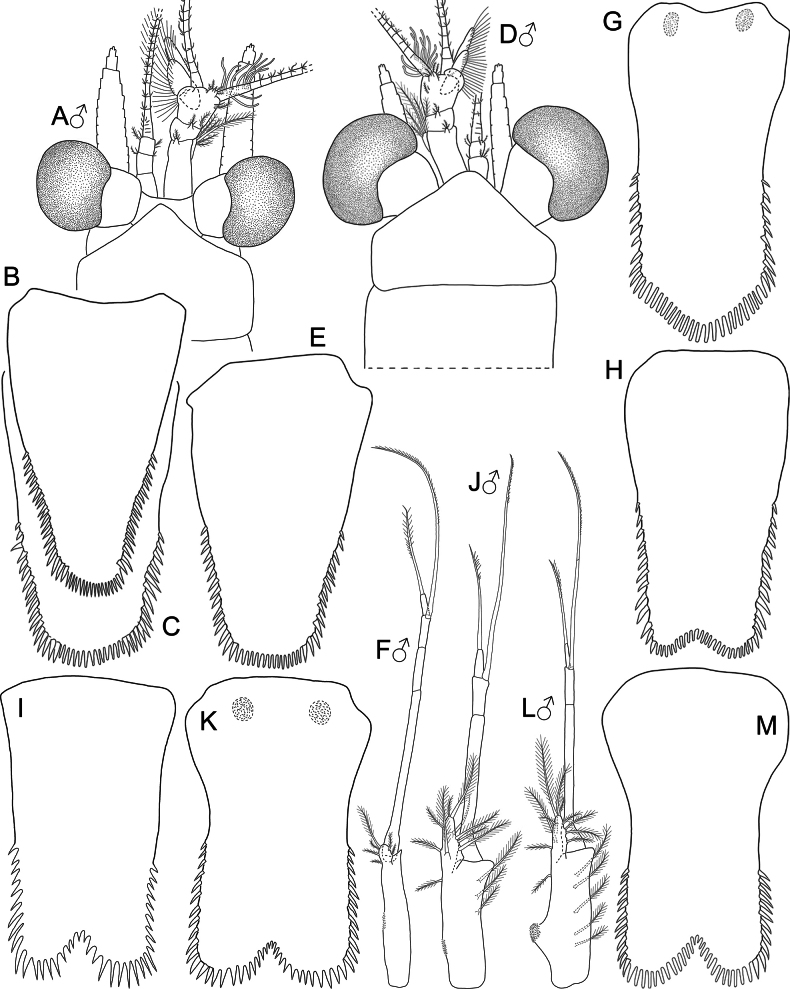
Differentiation between the species of *Mysidium* Dana, so far recorded in Caribbean caves. **A–C**. Cephalic region of male (**A**) and telson variants (**B, C**) in *Mysidium integrum* W.M. Tattersall; **D, E**. Cephalic region of male (**D**) and telson (**E**) in *M. cubanense* Băcescu & Ortiz; **F, G**. Fourth male pleopod (**F**) and telson (**G**) in *M. triangulare* Wittmann; **H**. Telson in *M. gracile* (Dana); **I**. Telson in *M. iliffei* Băcescu; **J, K**. Fourth male pleopod (**J**) and telson (**K**) in *M. antillarum* Wittmann; **L, M**. Fourth male pleopod (**L**) and telson (**M**) in *M. columbiae* (Zimmer). Modified after Wittmann and Wirtz 2019 (**A–H, J–M**) and Băcescu 1991 (**I**).

#### Mysidium (Mysidium) cubanense

Taxon classificationAnimaliaMysidaMysidae

Băcescu & Ortiz, 1984

DF6678D5-3A82-5F81-BB5F-4CBFE35F1602

Fig. 9D, E

Mysidium cubanense Băcescu & Ortiz, 1984: 18–20, fig. 2A–K (original description, record from small cave in Cuba); Harrison and Bowman 1987: 678 (in comparison); Băcescu 1991: 3, 4 (records, Jamaica, Bahamas); Price and Heard 2009: 938 (distribution); Ortiz et al. 2017b: 72–74, fig. 4 (distribution, diagnosis); Wittmann 2023: table SS1 (trogloxene status, sensory organs).
Mysidium (Mysidium) cubanense : Wittmann and Wirtz 2019: 12–14, tables 2, 3, fig. 3 (revised diagnosis, in key, cave record).
Mysidium cubanensis : Harrison and Bowman 1987: 674 (gender inconsistency).

##### Types examined.

Cuba • ***holotype***, ♂ ad. (BL = 4.5 mm; previously partly dissected; MINGA 49346) from entrance of small cave (Station 26 — type locality) • ***allotype***, ♀ ad. broken in two partly dissected pieces (MINGA 49347) from same sample as for holotype.

##### Non-types examined.

Leeward Antilles — **Curaçao** • 1 ♀ ad. (BL = 3.8 mm), 1 ♂ ad. (BL = 5.0 mm) from rock recesses at Sun Reef (Station 16) • 15 ♀♀ ad., 3 ♂♂ ad., 10 subad. (ZMH K-55259) from small marine cave at Playa Lagun (Station 18).

##### Short diagnosis.

*Mysidium* with large cornea, diameter >2.5× as long as terminal segment of antennular trunk; only males with anterior margin of antennular trunk dorsally with rounded, shield-like, mediodistal extension; thoracic endopod 3 with 3-segmented carpopropodus; male pleopod 3 with medial widening at ≈1/2 length; male pleopod 4 with 4-segmented exopod, endite of sympod reduced to weak medial hump or missing; telson linguiform to subrectangular or trapezoid, terminally not emarginated.

##### Distribution

(Fig. 7A). Widely distributed in coastal waters of the Gulf of Mexico, Caribbean and Bahamas in the range of 12–26°N, 68–82°W, 0–26 m depth (Băcescu 1991; Price and Heard 2009; Ortiz et al. 2017b; Wittmann and Wirtz 2019; Wittmann 2023). Present records (Stations 16, 18) from the coast of Curaçao. The species mainly forming swarms between corals, also found in caves and rock recesses.

#### Mysidium (Mysidium) gracile

Taxon classificationAnimaliaMysidaMysidae

(Dana, 1852)

8F7F45BA-C5D0-50D9-9910-9BC66103A617

Fig. 9H

Macromysis gracilis Dana, 1852: 653–655 (original description, combination with generic junior synonym).
Mysidium gracile : Czerniavsky 1887: 85–87 (revised combination, description); Price and Heard 2004: 155, fig. 3K (distribution, morphological remarks); Price and Heard 2009: 938 (distribution); Miyashita and Calliari 2014: 9, Table 1 (distribution); Ortiz et al. 2017b: 74, 75, fig. 5 (distribution, diagnosis); Ortiz and Lalana 2018: 67, 72, fig. 11 (in species list for Cuba, in key).
Mysidia gracilis : Zimmer 1915b: 215 (combination with generic junior homonym).
Mysidium (Mysidium) gracile : Wittmann and Ariani 2019: suppl. (fluorite statoliths, biogeography); Wittmann and Wirtz 2019: 6–8, Table 1, fig. 1 (revised diagnosis, in key).

##### Material examined.

Lesser Antilles — **St. Vincent** • 1 ♂ ad. (BL = 4.1 mm), 2 subad., 1 juv. from entrance of Bat Cave (Station 3) — **Martinique** • 1 ♂ ad. (BL = 6.6 mm) from entrance of Zeb Cave (Station 2) — **Saint-Martin** • 10 imm. (NHMW-ZOO-CR-31415) from small holes (Station 8) • 5 ♀♀ ad. (BL = 5.2–6.8 mm), 1 ♂ ad. (BL = 5.4 mm) from small holes (Station 7).

##### DNA data.

Eleven individuals of *M. gracile* were successfully amplified (548 and 658 bp fragments), ten from Basse Espagnole (Station 8), Saint-Martin, and one from Bat Cave (Station 3) in St. Vincent (GenBank PV990300–PV990310). 453 bp aligned together with *M. columbiae* and *M. triangulare* (Fig. 8), the Saint-Martin *M. gracile* displayed six closely-related (divergence 0.2–0.4%) different haplotypes. The St. Vincent sequence is quite divergent from the latter (16%), which no doubt reflects the great geographical distance (≈560 km) and natural habitat fragmentation between the two sites. Only one of the concerned substitutions is non-synonymous.

##### Short diagnosis.

*Mysidium* with large cornea, diameter 2–3× as long as terminal segment of antennular trunk; only males with anterior margin of antennular trunk dorsally with rounded, shield-like, mediodistal extension; antennal scale clearly reaching beyond antennular trunk; thoracic endopod 3 with 3-segmented carpopropodus; male pleopod 3 with medial widening at ≈1/2 length; male pleopod 4 with 4-segmented exopod, endite of sympod reduced to weak medial hump or missing. Telson subrectangular, terminally with shallow, rounded indentation.

##### Distribution and habitat

(Fig. 7B). Widely distributed, mainly in sublittoral, also in littoral waters of the western Atlantic, ranges 32°N to 23°S, 42–68°W, 3–35 m depth, including Bermuda, Gulf of Mexico, Caribbean and coast of Brazil (Price and Heard 2004; Ortiz et al. 2017b; Wittmann and Wirtz 2019). Type locality is the harbor of Rio de Janeiro, ca 22°54'S, 43°10'W (Dana 1852). Found in a great variety of marine to metahaline habitats. Swarms hovering during the daytime over the sea floor, also found between corals and associated with sea urchins and fish (Randall et al. 1964; Emery 1968). Present records in the Lesser Antilles are from small swarms at the entrance of caves (Stations 2, 3) or in small holes (Stations 7, 8), also recovered as a single specimen from a large swarm of *M. columbiae* in the entrance of Zeb Cave (Station 2). The great dominance of non-cave records classifies this species as trogloxene.

#### Mysidium (Mysidium) integrum

Taxon classificationAnimaliaMysidaMysidae

W.M. Tattersall, 1951

07F6ECDE-E0A5-5D4F-88CC-B24B28C37A59

Fig. 9A–C

Mysidium integrum W.M. Tattersall, 1951: 223–225, fig. 96 (original description); Brattegard 1970b: 128 (distribution); Brattegard 1973: 50, 51 (taxonomy, Caribbean coast); Modlin 1987a: 115 (records, Caribbean, Belize); Price and Heard 2004: 155, fig. 3J (distribution, morphological remarks); Price and Heard 2009: 938 (distribution); Ortiz et al. 2017b: 76, 77, fig. 7 (diagnosis, in key, distribution); Wittmann 2023: table SS1 (trogloxene status, sensory organs).
Mysidium (Mysidium) integrum : Wittmann and Ariani 2019: suppl. (fluorite statoliths, biogeography); Wittmann and Wirtz 2019: 9–12, tables 2, 3, fig. 2 (revised definition, in key, cave records).

##### Material examined.

Lesser Antilles — **Martinique** • 1 ♀ ad. (BL = 4.9 mm) from Zeb Cave (Station 2) — **Saint-Martin** • 1 ♀ ad. (BL = 3.9 mm), 1 ♂ ad. (BL = 4.4 mm) from small holes at Basse Espagnole (Station 8) — **Guadeloupe** • 1 ♀ ad. (BL = 5.1 mm) from entrance of Cathedral Cave (Station 10). Leeward Antilles — **Curaçao** • 6 ♂♂ ad. (BL = 3.4–4.9 mm), 3 ♀♀ ad. (BL = 3.3–4.3 mm), 4 ♀♀ subad., 3 ♂♂ subad., 4 imm. (NHMW-ZOO-CR-31420) from Blue Chamber (Station 15) • 19 ♀♀ ad., 19 ♂♂ ad., 1 ♀ subad., 1 juv. (ZMH-K-066772), sampling data as for preceding • 9 ♀♀ ad., 15 ♂♂ ad., 5 ♀ subad., 1 imm. (ZMH-K-066771) from entrance of small caves at Sun Reef (Station 17) • 10 ♀♀ ad., 16 ♂♂ ad., 2 ♀♀ subad., 2 ♂♂ subad. (MNHN-IU-2025-2831), sampling data as for preceding.

##### Short diagnosis.

*Mysidium* with cornea diameter <2.5× as long as terminal segment of antennular trunk; only males with anterior margin of antennular trunk dorsally with a small rounded, mediodistal extension; thoracic endopod 3 with 3-segmented carpopropodus; male pleopod 3 with medial widening at ≈1/2 length; male pleopod 4 with 4-segmented exopod, endite of sympod reduced to weak medial hump or missing; telson linguiform to subrectangular, with roughly straight or with convex, continuously rounded terminal margin.

##### Distribution and habitat

(Fig. 7C). Distribution most similar to that of *M. gracile*, namely widely distributed in marine and occasionally also in metahaline waters of the western Atlantic from Bermuda to Bahamas, Caribbean, Gulf of Mexico and coast of Brazil, range 32°N to 23°S, 42–69°W, 0–35 m depth (Brattegard 1970b, 1973; Ortiz et al. 2017b; Wittmann and Wirtz 2019). Type locality is tropical NW-Atlantic, Cruz Bay, St. John, Virgin Islands, ca 18°19.93'N, W 64°47.80'W (W.M. Tattersall 1951; Wittmann and Wirtz 2019). The present records (Stations 2, 8, 10, 15, 17) from cave entrances and small holes are within the already known range. In swarms close to the bottom of diverse coral reef and mangrove habitats, also found associated with sea urchins (Zoppi de Roa and Alonso 1997). The mysids were abundant at the entrance of caves and holes during the sampling in Curaçao (Stations 15, 17), but could be recovered only as rare individuals in Martinique (Station 2), Guadeloupe (Station 10) and Saint-Martin (Station 8), within swarms of other *Mysidium* species (*M. columbiae*, *M. gracile*).

#### Mysidium (Mysidium) triangulare

Taxon classificationAnimaliaMysidaMysidae

Wittmann in Wittmann & Wirtz, 2019

C8C1DB38-1FC2-5225-82F8-1384150E355E

Fig. 9F, G

Mysidium (Mysidium) triangulare Wittmann in Wittmann & Wirtz, 2019: 16–23, table 3, figs 5–8 (original description, in key, cave records); Wittmann and Ariani 2019: suppl. (fluorite statoliths, biogeography).
Mysidium triangulare : Hendrickx et al. 2023: 204 (in comparison); Wittmann 2023: table SS1 (trogloxene status, sensory organs).

##### Material examined.

Lesser Antilles — **Saint-Martin** • 3 ♀♀ ad. (BL = 4.7–6.1 mm), 5 ♂♂ ad. (BL = 4.4–4.9 mm) from small holes (Station 8). Leeward Antilles — **Curaçao** • 3 ♀♀ ad. (BL = 4.0–4.3 mm), 2 ♂♂ ad. (BL = 4.5–4.8 mm) (NHMW-ZOO-CR-31416) from Blue Chamber (Station 15) • 1 ♂ ad. (BL = 4.1 mm; ZMH-K-066773), sampling data as for preceding • 2 ♀♀ ad. (BL = 3.7–3.8 mm), 1 ♂ ad. (BL = 3.1 mm) (MNHN-IU-2025-2832) from entrance of small caves (Station 17).

##### DNA data.

Only two individuals were successfully amplified at the COI locus (615 and 658 bp fragments), both from Basse Espagnole (Station 8), Saint-Martin (GenBank PV990311 and PV990312). Over the aligned portion, they represented two different haplotypes that differed only by one (synonymous) substitution (0.16% divergence). In relation to the other *Mysidium* species (Fig. 8), they appeared as a well-separated species, equally distinct from *M. columbiae* and *M. gracile* (≈25% divergence).

##### Short diagnosis.

*Mysidium* with cornea diameter ≤2.5× length of terminal segment of antennular trunk; only males with anterior margin of antennular trunk dorsally with rounded, shield-like, mediodistal extension; thoracic endopod 3 with 3-segmented carpopropodus; male pleopod 3 with medial widening at ≈1/2 length; male pleopod 4 with 4-segmented exopod; telson spatulate, its terminal portion triangular with rounded tip.

##### Distribution and habitat

(Fig. 7D). Type locality is Caribbean, Curaçao, Playa Lagun, 12°19.09'N, 69°09.07'W, diverse habitats in 4–6 m depth (Wittmann and Wirtz 2019). This species previously known only from three sublittoral localities including an intertidal cave in euhaline waters off the island of Curaçao. The present findings from Curaçao (Stations 15, 17) and Saint-Martin (Station 8) extend the known geographic range to 12–18°N, 63–69°W, 3–28 m depth. By including these records, *M. triangulare* appears to be a troglophile rather than trogloxene as previously suggested by Wittmann (2023). Previously known from mostly large swarms hovering during daytime around and between corals. The present records always yielded small numbers, mostly at the entrance of caves or holes, only one specimen inside an intertidal cave. At Saint-Martin the mysids were captured from small swarms of *M. gracile*.

#### Mysidium (Orientomysidium) antillarum

Taxon classificationAnimaliaMysidaMysidae

Wittmann, 2019

7BFD586A-B7CB-5152-A5C1-6937F51E0A02

Fig. 9J, K

**Figure 10.**
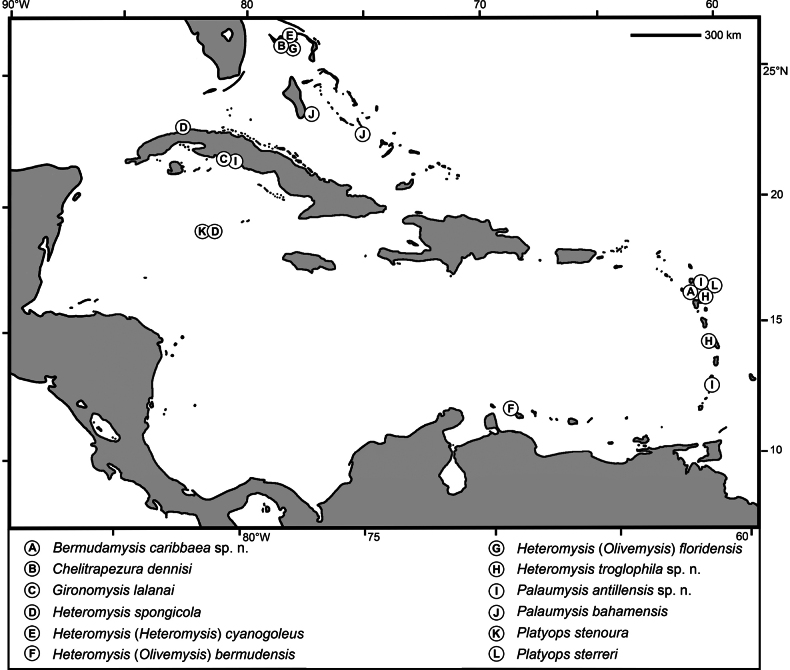
Distribution of the species of **A, K, L**. Mysidetini; **B, D–H**. Heteromysini; **C, I, J**. Palaumysinae in marine caves of the Caribbean and adjacent areas. Coinciding points slightly displaced.

**Figure 11.**
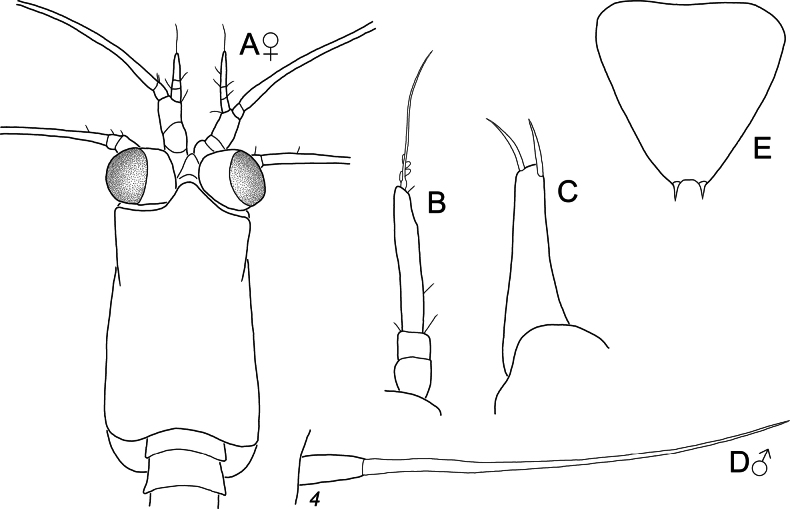
Selected diagnostic characters of *Gironomysis lalanai* Ortiz, García-Debrás & Pérez. **A**. Cephalothorax of female; **B**. Mesial flagellum of antennula; **C**. Antennal scale; **D**. Fourth male pleopod; **E**. Telson. Modified after Ortiz et al. 1997.

**Figure 12.**
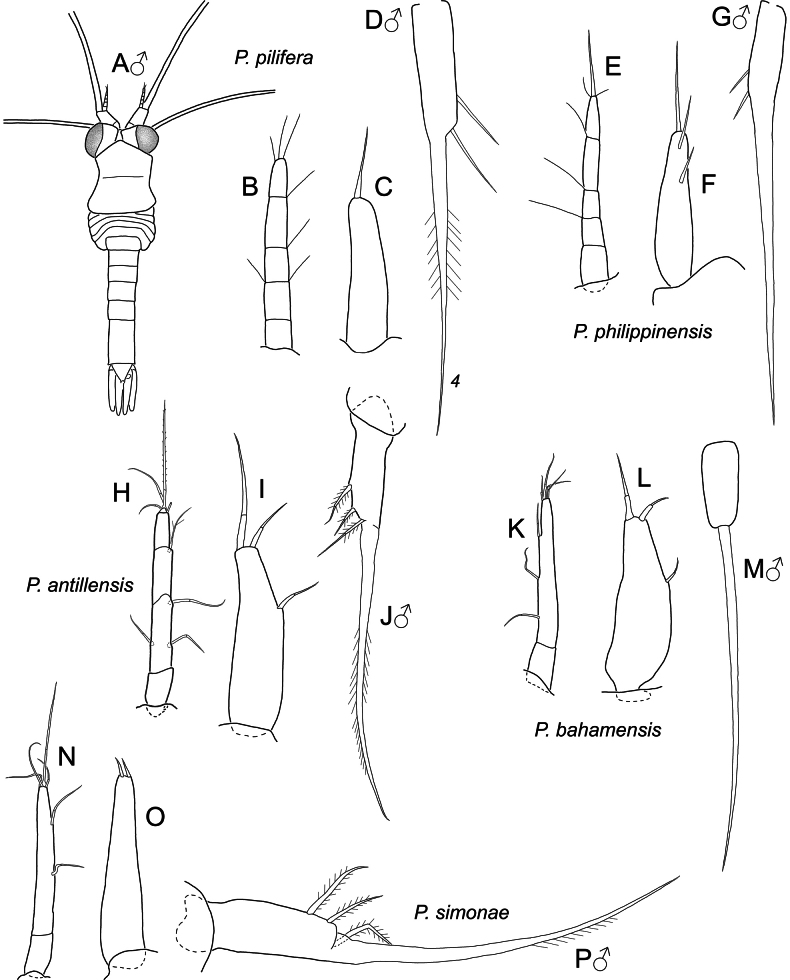
Differentiation between the species of *Palaumysis* Băcescu & Iliffe. **A–D**. Habitus of ♂, dorsal, most appendages omitted (**A**), mesial flagellum of antennula (**B**), antennal scale (**C**) and fourth male pleopod (**D**) in *P. pilifera* Hanamura & Kase; **E–G**. Mesial flagellum of antennula (**E**), antennal scale (**F**) and fourth male pleopod (**G**) in *P. philippinensis* Hanamura & Kase; **H–J**. Mesial flagellum of antennula (**H**), antennal scale (**I**) and fourth male pleopod (**J**) in *P. antillensis* sp. nov.; **K–M**. Mesial flagellum of antennula (**K**), antennal scale (**L**) and fourth male pleopod (**M**) in *P. bahamensis* Pesce & Iliffe; **N–P**. Mesial flagellum of antennula (**N**), antennal scale (**O**) and fourth male pleopod (**P**) in *P. simonae* Băcescu & Iliffe. Modified after Hanamura and Kase 2003 (**A–D**), Hanamura and Kase 2002 (**E–G**) and Pesce and Iliffe 2002 (**K–M**); original drawings (**H–J, N–P**).

**Figure 13.**
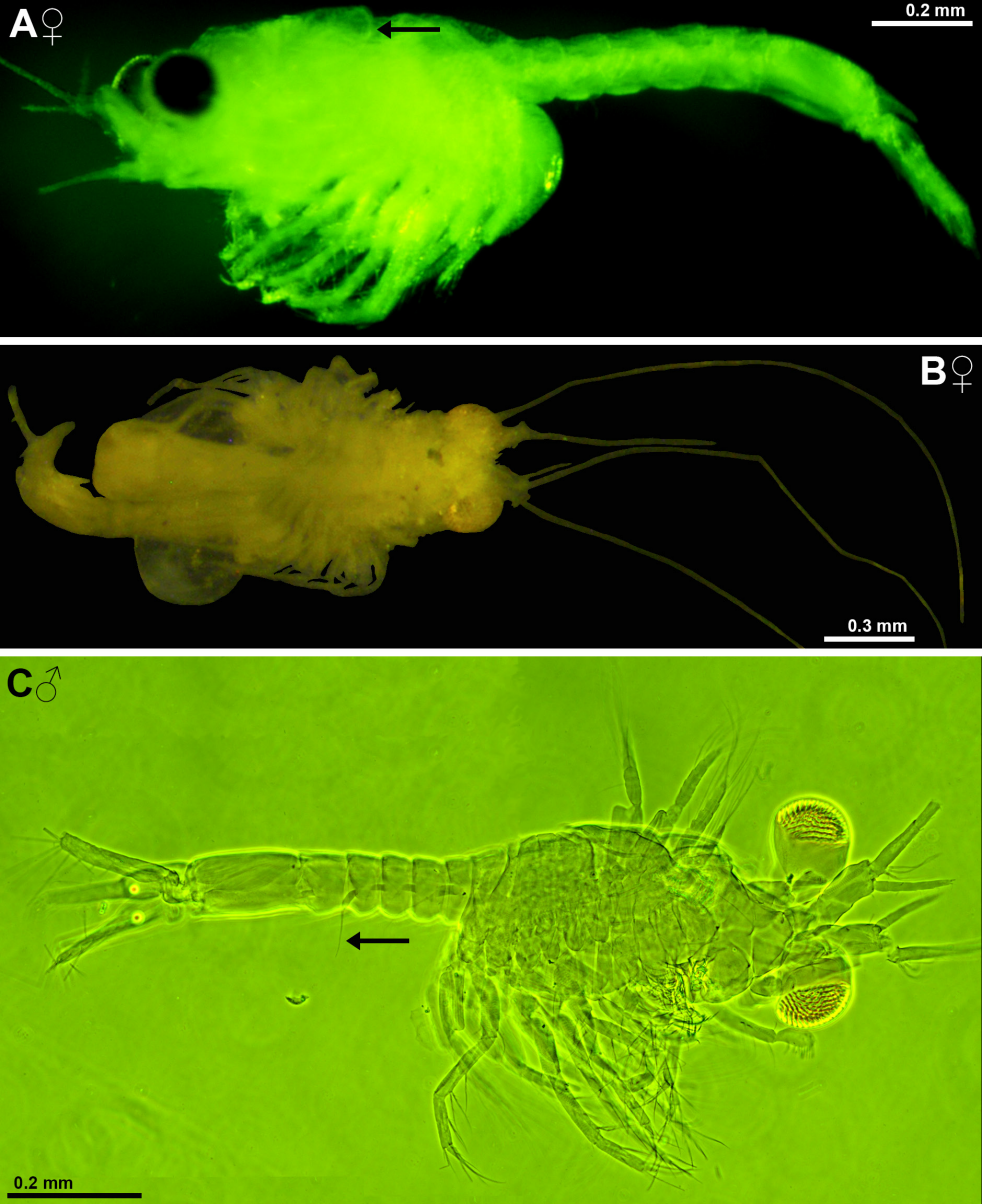
Habitus of *Palaumysis simonae* Băcescu & Iliffe adults from submarine caves at Palau Islands. **A**. Non-type ♀, lateral, arrow points to posterior margin of carapace; **B**. Holotype ♀ carrying an (accidentally somewhat caudally shifted) postnauplioid larva, ventral, the adult pleon bent ventrally, thus extending out of focus; **C**. Non-type ♂ bleached on slide, ventral, focus on pleopod 4 pointed by arrow.

**Figure 14.**
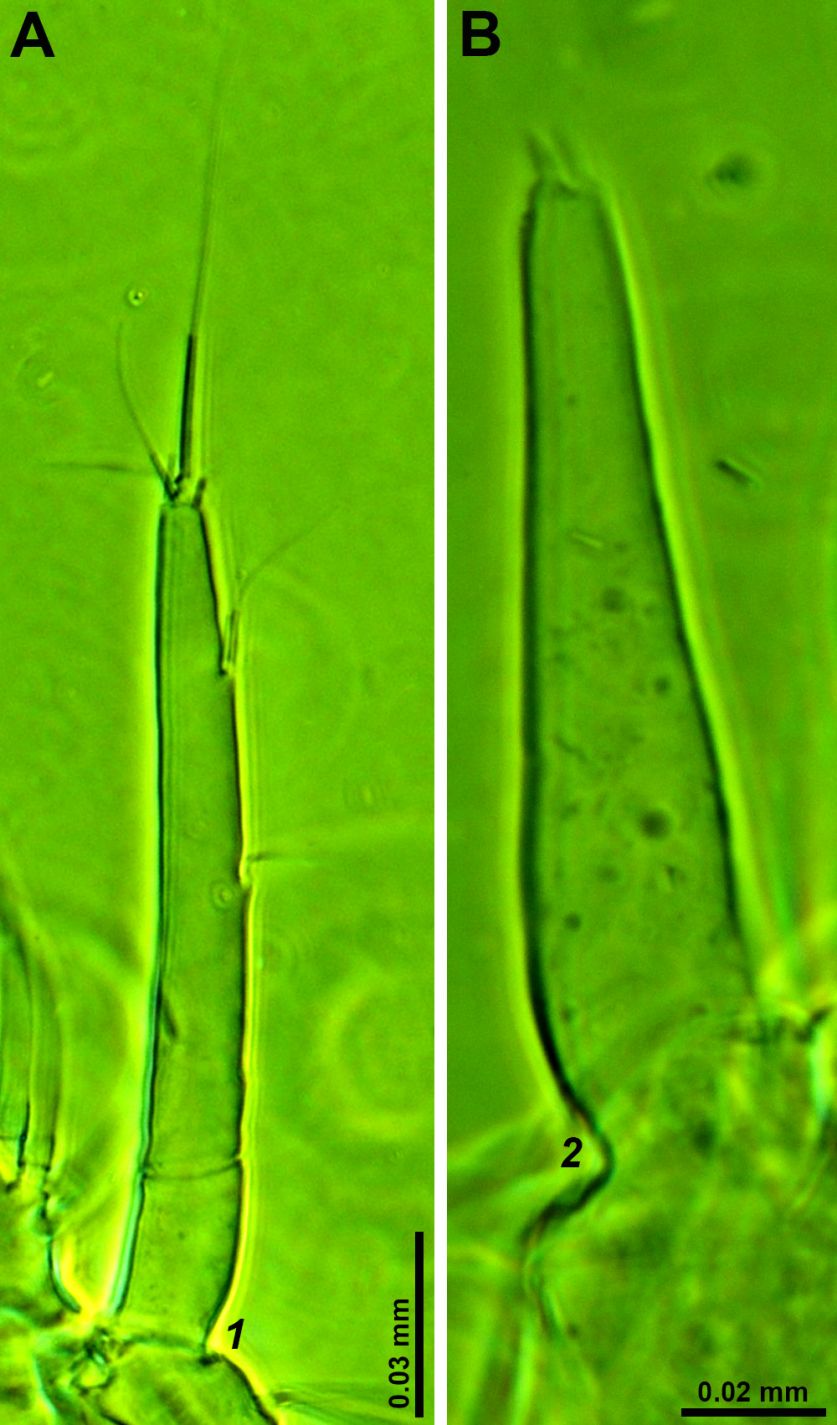
Antennula and antenna of *Palaumysis simonae* Băcescu & Iliffe ♀ from submarine cave near Soft Coral Arch, Palau Islands. **A**. Mesial antennular flagellum; **B**. Antennal scale, dorsal.

Mysidium (Orientomysidium) antillarum Wittmann in Wittmann & Wirtz, 2019: 30–37, table 3, figs 10–14 (original description, in key, cave records); Wittmann and Ariani 2019: suppl. (fluorite statoliths, biogeography).
Mysidium antillarum : Hendrickx et al. 2023: 203 (in citation, distribution).

##### Material examined.

Lesser Antilles — **Bequia** • 2 ♂♂ ad. (BL = 7.5–7.6 mm) from Full Moon Cave (Station 6). Leeward Antilles — **Curaçao** • 1 ♀ ad., 4 ♀♀ subad., 3 ♂♂ subad., 12 imm. (MNHN-IU-2025-2829) from entrance of small caves (Station 17) • 2 ♀♀ ad. (BL = 6.3–7.4 mm), 2 ♂♂ ad. (BL = 7.1–7.2 mm) (NHMW-ZOO-CR-31417), sampling data as for preceding • 1 ♂ ad., 5 ♀♀ subad., 4 ♂♂ subad., 10 imm. (ZMH-K-066769), sampling data as for preceding.

##### Short diagnosis.

*Mysidium* with cornea diameter 1.4–1.7× as long as terminal segment of antennular trunk; only males with anterior margin of antennular trunk dorsally with rounded, shield-like, mediodistal extension; antennal scale extends clearly beyond antennular trunk; thoracic endopod 3 with 3-segmented carpopropodus; male pleopod 4 with 3-segmented exopod, endite of sympod reduced to indistinct projection; telson subrectangular with distinctly concave lateral margins and with distinct apical cleft separating two broadly rounded, apical lobes; acute laminae on terminal margins of telson and its cleft.

##### Distribution and habitat

(Fig. 7E). Type locality is Caribbean, Curaçao, Reef Sint Marie, 12°12.73'N, 69°05.10'W, 0–0.5 m depth (Wittmann and Wirtz 2019). This species previously known only from six localities off the island of Curaçao plus one from Bonaire. The present records from caves in Bequia (Station 6) and Curaçao (Station 17) extend the known range to 12–13°N, 61–69°W, depth 0–28 m. The mysids are known from sublittoral to littoral euhaline waters where they form swarms closely hovering above the sea floor over diverse substrates, also found inside small caves (Wittmann and Wirtz 2019). The present first record from a large diveable cave, namely the Full Moon Cave in Bequia (Station 6), is based on only two specimens among a large swarm of *M. columbiae*. The great dominance of non-cave records classifies *M. antillarum* as trogloxene.

#### Mysidium (Orientomysidium) columbiae

Taxon classificationAnimaliaMysidaMysidae

(Zimmer, 1915)

2F132707-6426-5053-B150-5210BBD8DA0F

Fig. 9L, M

Diamysis columbiae Zimmer, 1915a: 172–174, figs 23–29 (outdated senior synonym); Zimmer 1915b: 215 (in synonymy).
Mysidia columbiae : Zimmer 1915b: 215, fig. 18 (recombination with generic junior homonym).
Mysidium columbiae : W.M. Tattersall 1951: 222, 223 (revised combination); Brattegard 1969: 86–88, fig. 27B (in part, record, description); Price and Heard 2004: 154, fig. 3I (distribution); Miyashita and Calliari 2014: 9, Table 1 (distribution).
Mysidium colombiae : Price and Heard 2009: 938 (misspelling, distribution).
Mysidium (Orientomysidium) columbiae : Wittmann in Wittmann & Wirtz, 2019: 27–30, tables 2, 3, figs 9, 13E–J (subgeneric combination, description, in key); Wittmann and Ariani 2019: suppl. (fluorite statoliths, biogeography).
nec Mysidium columbiae: Brattegard 1969: fig. 27E (male pleopod 4 not matching).

##### Material examined.

Lesser Antilles — **Martinique** • 20 ♀♀ ad., 9 ♂♂ ad., 2 ♀♀ subad. (MNHN-IU-2025-2830) from Zeb Cave (Station 2) — **SW-Bequia** • 4 ♂♂ ad., 3 ♂♂ subad., 8 ♀♀ subad., 15 imm. (ZMH-K-066770) from Full Moon Cave (Station 6) — **Guadeloupe** • 3 ♀♀ ad. (BL = 7.6–8.1 mm), 2 ♂♂ ad. (BL = 7.0–7.5 mm) from Barracuda Cave (Station 11) • 5 ♀♀ ad., 5 ♂♂ ad., 2 ♂♂ subad., 3 imm. (NHMW-ZOO-CR-31418) from Cathedral Cave (Station 10). Leeward Antilles — **Curaçao** • 1 ♀ subad. (BL = 3.5 mm) from entrance of small cave at Sun Reef (Station 17).

##### DNA data.

At the COI locus, 15 individuals were amplified (545 and 658 bp fragments), three from Full Moon Cave (Station 6), Bequia (one single haplotype), two from Zeb Cave (Station 2) in Martinique (2 haplotypes) and ten (4 haplotypes) from Cathedral Cave (Station 10), Guadeloupe (GenBank PV990313–PV990327). All substitutions are synonymous. Aligned 453 bp together with other two *Mysidium* species from this study (Fig. 8), the Martinique and Guadeloupe individuals are differentiated but grouped, with only ≈1% divergence between the two islands (geographic distance 230 km). The divergence between these two and the Bequia sequences, however, is as high as ≈11% for a minimum geographic distance (Bequia-Martinique) of 170 km. This indicates a much stronger barrier to *M. columbiae*’s gene flow south of Martinique than north of this island.

##### Short diagnosis.

*Mysidium* with cornea diameter 1.5–2.0× as long as terminal segment of antennular trunk; only males with anterior margin of antennular trunk dorsally with rounded, shield-like, mediodistal extension; antennal scale extending far beyond antennular trunk (again more than in Fig. 9A); thoracic endopod 3 with 3-segmented carpopropodus; male pleopod 4 with 3-segmented exopod, its sympod with strong endite at 2/5 sympod length from basis; telson subrectangular with distinctly concave lateral margins and with distinct apical cleft separating two broadly rounded, apical lobes; blunt laminae on terminal margins of telson and its cleft.

##### Distribution and habitat

(Fig. 7F). Type locality is Caribbean coast of Colombia, Cartagena, ca 10°22.2'N, 75°31.2'W (Zimmer 1915a; coordinates estimated by Wittmann and Wirtz 2019). Widely distributed in coastal marine waters of the western Atlantic: Bahamas, Caribbean, southern Gulf of Mexico and Brazil, 24°N to 8°S (Price and Heard 2004; Miyashita and Calliari 2014; Wittmann and Wirtz 2019; present data). The present samples from small up to diveable caves in Bequia (Station 6), Martinique (Station 2), Guadeloupe (Stations 10, 11) and Curaçao (Station 17) are within this range. Wittmann and Wirtz (2019) observed swarms hovering around and between corals during daytime and dispersing over the sea floor during the night in 3–26 m depth. Some of the present records were made during daytime from sometimes big swarms at the entrance of caves or at mid-distance from the entrance in the semi-dark zone. Nonetheless, this species is considered trogloxene due to the great dominance of non-cave records in the extensive literature.

#### Mysidium iliffei

Taxon classificationAnimaliaMysidaMysidae

Băcescu, 1991

05A7CA66-7334-5F4D-A543-4498D1142644

Fig. 9I

Mysidium iliffei Băcescu, 1991: 3–5 (original description); Ortiz et al. 2017a: Table 1 (diagnostic characters); Wittmann and Wirtz 2019: 37, 38, Table 2 (revised definition, type locality, distribution, in key).
Mysidium illifei : Băcescu, 1991: species name misspelled in legend of fig. 1.

##### Note.

The types of *M. iliffei* are missing; there is no entry in the inventory booklet of the MINGA, and no other material is available. This poorly known species was not assigned at the subgeneric level by Wittmann and Wirtz (2019).

##### Short diagnosis.

Based on adult females and subadult males. *Mysidium* with cornea diameter 1.5× as long as terminal segment of antennular trunk; antennal scale not extending beyond antennular trunk; thoracic endopod 3 with 3-segmented carpopropodus; telson rectangular with approx. straight lateral margins and with distinct apical cleft separating two broadly rounded, apical lobes; acute laminae on terminal margins of telson and its cleft.

##### Distribution

(Fig. 7G). Băcescu (1991) first described *M. iliffei* from material collected with *M. cubanense* by T.M. Iliffe in Jamaica. According to the sampling protocol by T.M. Iliffe (pers. comm. 09 March 2018; excerpt published by Wittmann and Wirtz 2019) the materials of *M. iliffei* were sampled from mysid swarms in the entrance of Joseph’s Caves, touristic sea cave in Jamaica, 18°16.08'N, 78°22.09'W, depth 1–3 m. The species is provisionally classified as a troglophile.

#### Subfamily Palaumysinae

Taxon classificationAnimaliaMysidaMysidae

Wittmann, 2013

D677D726-DD76-5AEB-AC20-8A3683DA34B2

Mancomysini Băcescu & Iliffe, 1986b: 32 (diagnosis, no type genus); Ortiz et al. 2012: 988 (in key); Meland et al. 2015: 18, table 3 (nomen nudum, synonymy).
Mancomysinae : Meland and Willassen 2007: 1099, app. A (molecular phylogeny, revised rank); Petrescu and Wittmann 2009: 58 (type collection); Chevaldonné et al. 2015: fig. 5 (molecular systematics).
Palaumysinae Wittmann, 2013: 391 (replacement name); Wittmann et al. 2014: 332, 340, figs 4, 5, 21 (diagnosis, morphology, taxonomy); Ortiz and Lalana 2018: 67 (in list, Cuba); Kou et al. 2025: 5 (molecular phylogeny).

##### Diagnosis.

Updated from Wittmann et al. (2014). Mysidae with well-developed eyes, cornea calotte-shaped; antennular trunk robust, no male lobe; mesial antennular flagellum rudimentary, with 2–5 segments depending on species; antennal scale vestigial, unsegmented; labrum rostrally rounded, no distinct rostral process; thoracic endopods 3–8 with carpus separated from propodus by a transverse articulation, unless (in most species) carpopropodus undivided; two pairs of well-developed oostegites; penes of moderate size; all pleopods reduced to uniramous, unsegmented rods in both sexes, pleopod 4 of adult males with large, modified apical seta; both rami of the uropods undivided, endopod with statocyst as far as known composed of fluorite; telson short (sub)triangular, without or with small medio-apical indentation, its lateral margins bare except for each ending in a spine or tooth-like projection in paramedian position, no additional spines, no setae.

##### Type genus.

*Palaumysis* Băcescu & Iliffe, 1986.

##### Genus inventory.

*Gironomysis* Ortiz, García-Debrás & Pérez, 1997 (1 species) and *Palaumysis* (5).

#### Genus Gironomysis

Taxon classificationAnimaliaMysidaMysidae

Ortiz, García-Debrás & Pérez, 1997

BDE491CF-F94F-50C6-87E1-5D5454926F55

Gironomysis Ortiz et al., 1997: 22 (diagnosis, original description); Ortiz et al. 2012: 988, Table 1, fig. 12 (in key to genera, in species list, Caribbean); Wittmann et al. 2014: 340 (taxonomy); Ortiz and Lalana 2018: 67 (in taxa list, in key).

##### Short diagnosis.

Palaumysinae with normal-sized carapace leaving 1–2 ultimate thoracomeres mid-dorsally exposed; thoracic endopods 3–8 with unsegmented or 2-segmented carpopropodus; endopod of uropods setose all around, longer than exopod, no spines; lateral margin of exopod with only few setae on distal half.

#### Gironomysis lalanai

Taxon classificationAnimaliaMysidaMysidae

Ortiz, García-Debrás & Pérez, 1997

4EDF50A5-2DAA-5E9A-93DA-1CFF40CE9DAD

Fig. 11

Gironomysis lalanai Ortiz et al., 1997: 22, 27, figs 1–25 (diagnosis, original description); Ortiz and Lalana 2018: 67, fig. 6 (in list, Cuban archipelago).

##### Short diagnosis.

*Gironomysis* with 3-segmented mesial antennular flagellum; long, well-developed lateral flagellum of antennula with four or five apically blunt aesthetascs on basal segment; rudimentary antennal scale distally with two medium-sized setae, no spines, no teeth; male pleopod 4 with long smooth apical seta; lateral margin of exopod of uropods with only two setae on distal half.

##### Note.

Ortiz et al. (1997) misinterpreted the “widened setae” (aesthetascs) at the basal segment of the lateral antennular flagellum as representing the male lobe (see below remarks on the same subject in *Palaumysis* species).

##### Distribution

(Fig. 10C). Known only from the Casimba Laguna Larga, a brackish well in the cave system of Playa Girón (near 22°04.54'N, 81°03.37'W) at the Caribbean coast of the province Matanzas, Cuba (Ortiz et al. 1997).

#### Genus Palaumysis

Taxon classificationAnimaliaMysidaMysidae

Băcescu & Iliffe, 1986

ECE6D534-F561-5A1D-8B26-9AB1CCA788B6

Figs 12, 13, 14, 15, 16, 17, 18, 19, 20, 21, 22, 23, 24, 25, 26, 27, 28

Palaumysis Băcescu & Iliffe, 1986b: 31 (diagnosis); Hanamura and Kase 2002: 254, 255 (revision, revised definition).

##### Diagnosis.

Modified after Hanamura and Kase (2002) for inclusion of features of *P. bahamensis* Băcescu & Iliffe, 1986, and *P. antillensis* sp. nov. Palaumysinae with long, well-developed lateral flagellum of antennula with 3–5 apically blunt aesthetascs on basal segment in both sexes; antennal scale with a few setae, no spines, no teeth; carapace short, leaving posterior four or five thoracomeres mid-dorsally exposed (in adults); thoracic endopods 3–8 with unsegmented carpopropodus; pleopod 4 with long apical seta in adult males (rarely in adult females); endopod of uropods setose all around, longer than exopod, no spines; lateral margin of exopod with only few setae on distal half.

##### Type species.

*Palaumysis simonae* Băcescu & Iliffe, 1986, by monotypy.

##### Species inventory.

Five species now included at a world-wide scale; bold italics highlight two species recorded from Caribbean caves: *Palaumysis pilifera* Hanamura & Kase, 2003, *P. philippinensis* Hanamura & Kase, 2002, ***P. antillensis*** sp. nov., ***P. bahamensis*** Pesce & Iliffe, 2002, and *P. simonae* Băcescu & Iliffe, 1986. See also below global key to the genera and species of Palaumysinae.

##### Differentiation between *Palaumysis* species.

The now five known species of *Palaumysis* are well distinguished by a range of minor but significant characters. Three morpho-geographic groups may be distinguished: (1) the tropical W-Pacific *P. simonae* with the antennal scale bearing only one or two minute setae at the apex vs a large seta accompanied by 0–2 small setae at the apex in all remaining species; (2) NW-Pacific species, namely *P. philippinensis* and *P. pilifera* with a (sub)rectangular posteroventral edge of the carapace vs well-rounded edge in groups 1 and 3; (3) subtropical NW-Atlantic species, namely *P. bahamensis* and *P. antillensis* sp. nov., characterized by combination of a large seta accompanied by 0–2 small setae at the apex of the antennal scale as in (2) and a rounded posteroventral carapace edge as in (1).

##### Remarks.

Băcescu and Iliffe (1986b) noted upon original description of *P. simonae* “... ± pentagonal zones on the ventral side of each pleonite ...” and listed these “zones” among the main diagnostic features for the definition of the tribe Mancomysini Băcescu & Iliffe, 1986, today considered a nomen nudum due to the missing type genus and, consequently, replaced by Palaumysinae Wittmann, 2013. As shown above, near-pentagonal images are formed by pairs of paramedian muscle structures inside pleomeres, also found in *P. antillensis* sp. nov. (Fig. 27D) and *Bermudamysis speluncola* (Fig. 36E). Hanamura and Kase (2002) made additional corrections upon redescription based on types of *P. simonae*. Aesthetascs drawn by Băcescu and Iliffe (1986b: fig. 2A) as inserting on a prominence of the distal margin of the antennular trunk actually insert on the lateral antennular flagellum as usual in Mysidae (Fig. 38A, B). Upon original description of *P. bahamensis*, Pesce and Iliffe (2002: 273, fig. 13) followed Băcescu and Iliffe (1986b) by displacing the aesthetascs to the same position. Both author groups interpreted the respective mid-dorsal prominence as a rudiment of the (ventral) male lobe without discussing that this prominence is positioned dorsally and present in both sexes. In contrast, the prominence is here interpreted as the mid-dorsal lobe in subterminal position on the antennular trunk (Figs 24A, 38A); it bears no such aesthetascs but a few small barbed setae as in most species of Mysidae; moreover, this lobe bears teeth or spines in addition to setae in many genera other than *Palaumysis*. Upon original description of the closely related *Gironomysis lalanai*, Ortiz et al. (1997: fig. 12) drew the aesthetascs in the correct position but nonetheless followed the erroneous interpretation as remnants of the male lobe by Băcescu and Iliffe (1986b).

#### Palaumysis simonae

Taxon classificationAnimaliaMysidaMysidae

Băcescu & Iliffe, 1986

92F49FA0-ECA1-5953-9974-F0B7D96FC3AD

Figs 12N–P, 13–16

Palaumysis simonae Băcescu & Iliffe, 1986b: 31–34, fig. 2 (original description); Hanamura and Kase 2002: 255–257, figs 1, 2 (revised description); Hanamura and Kase 2003: 151, 152, Table 1, figs 4a, b, 5 (supplementary description); Hanamura and De Grave 2004: 66, 67 (records); Meland and Willassen 2007: fig. 3 (phylogeny, rRNA sequencing); Petrescu and Wittmann 2009: 58 (type repository, etymology); Wittmann 2013: Table 1, fig. 4A (male genital apparatus); Wittmann et al. 2014: figs 5, 42 (female habitus, molecular phylogeny); Chevaldonné et al. 2015: fig. 5 (molecular systematics); Wittmann and Ariani 2019: suppl. (statolith composition, biogeography); Wittmann 2023: table SS1 (eye structure); Kou et al. 2025: figs 8–10 (molecular phylogeny).

##### Note.

This non-Caribbean species treated here for differentiation from *P. bahamensis* and *P. antillensis* sp. nov.

##### Types examined.

Palau Archipelago • ***holotype***, ♀ ad. (BL = 1.20 mm; MINGA 49449) from Chandelier Cave (Station 36: type locality) • ***paratypes***, 2 ♀♀ ad. (BL = 1.10–1.35 mm; MINGA 49448), sampling data as for holotype.

##### Non-types examined.

Palau Archipelago • 1 ♀ ad., 4 ♂♂ ad., 4 imm. (MNHN-IU-2025-2836) from unnamed cave near Soft Coral Arch (Station 37) • 4 ♀♀ ad., 2 ♂♂ ad., 2 imm. (NHMW-ZOO-CR-31419), sampling data as for preceding • 3 ♀♀ ad., 3 ♂♂ ad., 1 ♀ subad., 2 imm. (ZMH-K-066764), sampling data as for preceding • 1 ♀ ad., 1 ♂ ad., 2 imm. (MNHN-IU-2025-2837) from Ishura Cave (Station 38) • 2 ♀♀ ad., 1 ♂ ad., 1 imm. (NHMW-ZOO-CR-31427), sampling data as for preceding • 1 ♂ ad., 3 imm. (ZMH-K-066765), sampling data as for preceding.

##### Distribution.

This species so far known only from submarine caves in the Palau archipelago.

##### Revised diagnosis.

*Palaumysis* with mesial antennular flagellum 2- to 4-segmented, basal segment shortest, apical segment with 4/5 flagellum length in 2-segmented flagella vs 1/5 in 3- to 4-segmented flagella; length of antennal scale 4–5× maximum width, apex with one or two (mostly 2) minute setae, lateral margins bare; post-cervical lateral margins of carapace without or with small, projecting lobe; apex of pleopod 1 with large plumose (barbed) seta only in males caudally overreaching pleopod 2; apex of male pleopod 4 with one large and one short seta, the former reaching at most to apex of pleopod 5 and bearing several minute cils near 2/3 length, lateral margin of pleopod 4 with one or two short setae; pleopod 4 mostly normal in adult females, but male-like with large apical seta in a few specimens with fully developed brood pouch.

##### Notes on holotype

(Fig. 13B). Small brush of aesthetascs on basal segment of mesial antennular flagellum. Cornea spherical with diameter measuring 10% BL in lateral view. Pigment not retracted from cornea. Paramedian pairs of muscle structures, visible as one or two near-pentagonal images (as in Fig. 16D) per segment, located in pleomeres 2–5 between neuronal cord and intestine.

##### Supplementary description.

♀♀ ad. with BL range 1.42–1.77 mm, TL of 1.52–1.93 mm (*n* = 11) and ♂♂ ad. with BL range 0.97–1.62 mm, TL of 1.25–1.88 mm (*n* = 12) in the present material. Hanamura and Kase (2002) obtained BL data within the present ranges. Băcescu and Iliffe (1986b) reported TL of 1.6–2 mm without differentiation between sexes.

***Cephalothorax*** (Fig. 13). Basal segment of the 2- to 4-segmented mesial antennular flagellum without setae; second to penultimate segments (if more than two segments) each with one or two short whip setae at disto-mesial edge; apex with one long plus 3–5 short whip setae (Fig. 14A). Carapace short (Fig. 13A), leaving four or five ultimate thoracic segments dorsally exposed. Pigment not or only weakly retracted from crystalline bodies of the eye. Labrum rostrally convex, broadly rounded.

***Thoracopods***. Flagellum of exopods 1–8 with 6, 7, 7, 7, 7, 7, 7, and 6 or 7 segments, respectively. Endopods 3–8 with unsegmented carpopropodus. Dactylus 1 with strong nail, length as in Fig. 25C though less bent; distal half of inner (concave) margin of this nail microserrated by minute stiff cils; nails 2–8 as given below for *P. antillensis* sp. nov. (Fig. 25F, H, K). Thoracic endopods 3–8 with minute triangular scales on ischium and merus as in *P. antillensis* sp. nov. (Fig. 26D, E).

**Figure 15.**
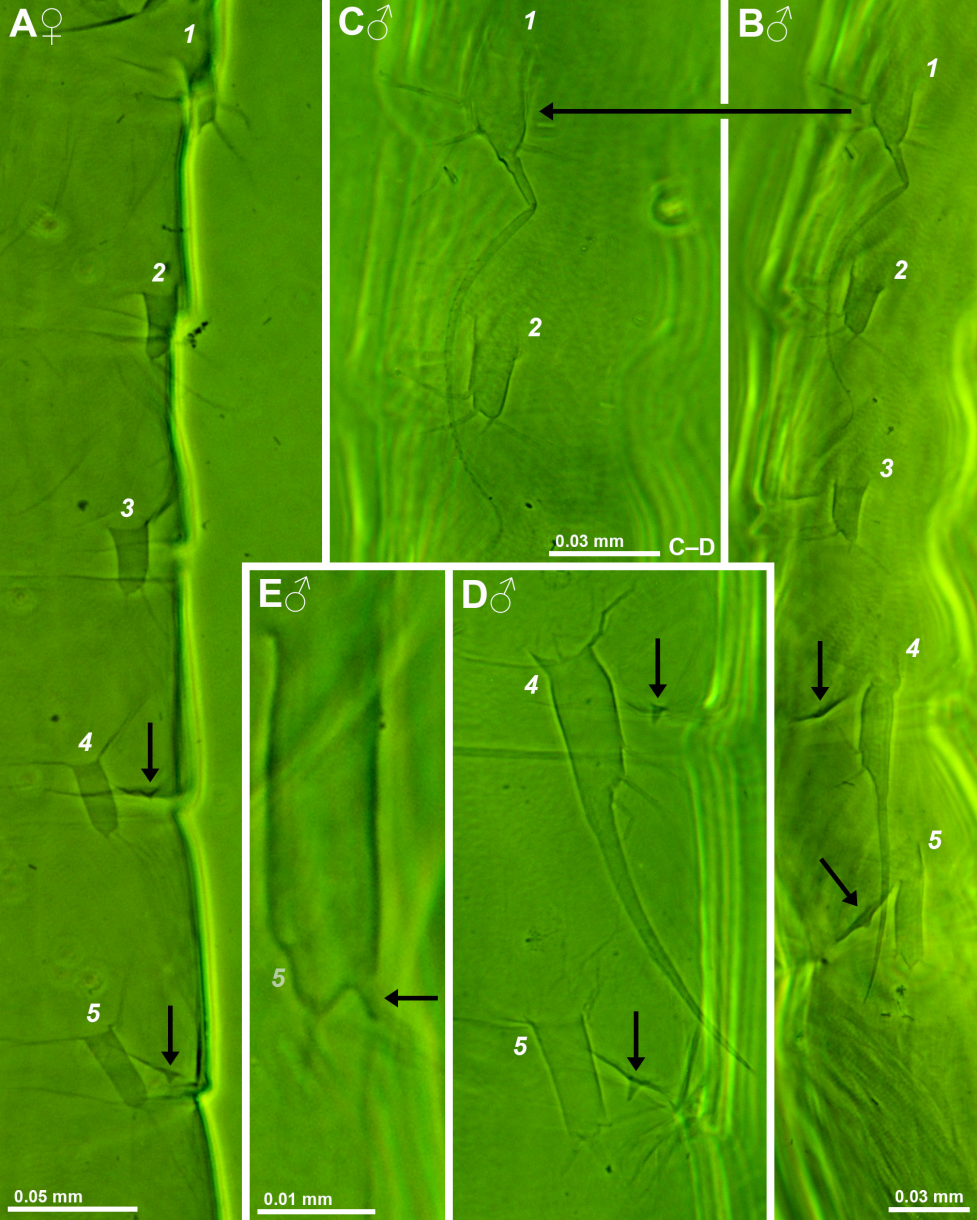
Pleopods of *Palaumysis simonae* Băcescu & Iliffe, material from submarine cave near Soft Coral Arch, Palau Islands. **A**. Left female pleopods 1–5, ventral; **B**. Right male pleopods 1–5, obliquely lateral; **C**. Detail of (**B**) showing pleopods 1, 2; **D**. Left male pleopods 4, 5, ventral; **E**. Right male pleopod 5, lateral, arrow points to spine-like seta; **A, B, D**. Sternites 4 and 5 with short arrows pointing to elevations varying from having a widely obtuse-angled caudal margin (B: *4*) up to bearing an acute tooth-like extension (D: *5*).

**Figure 16.**
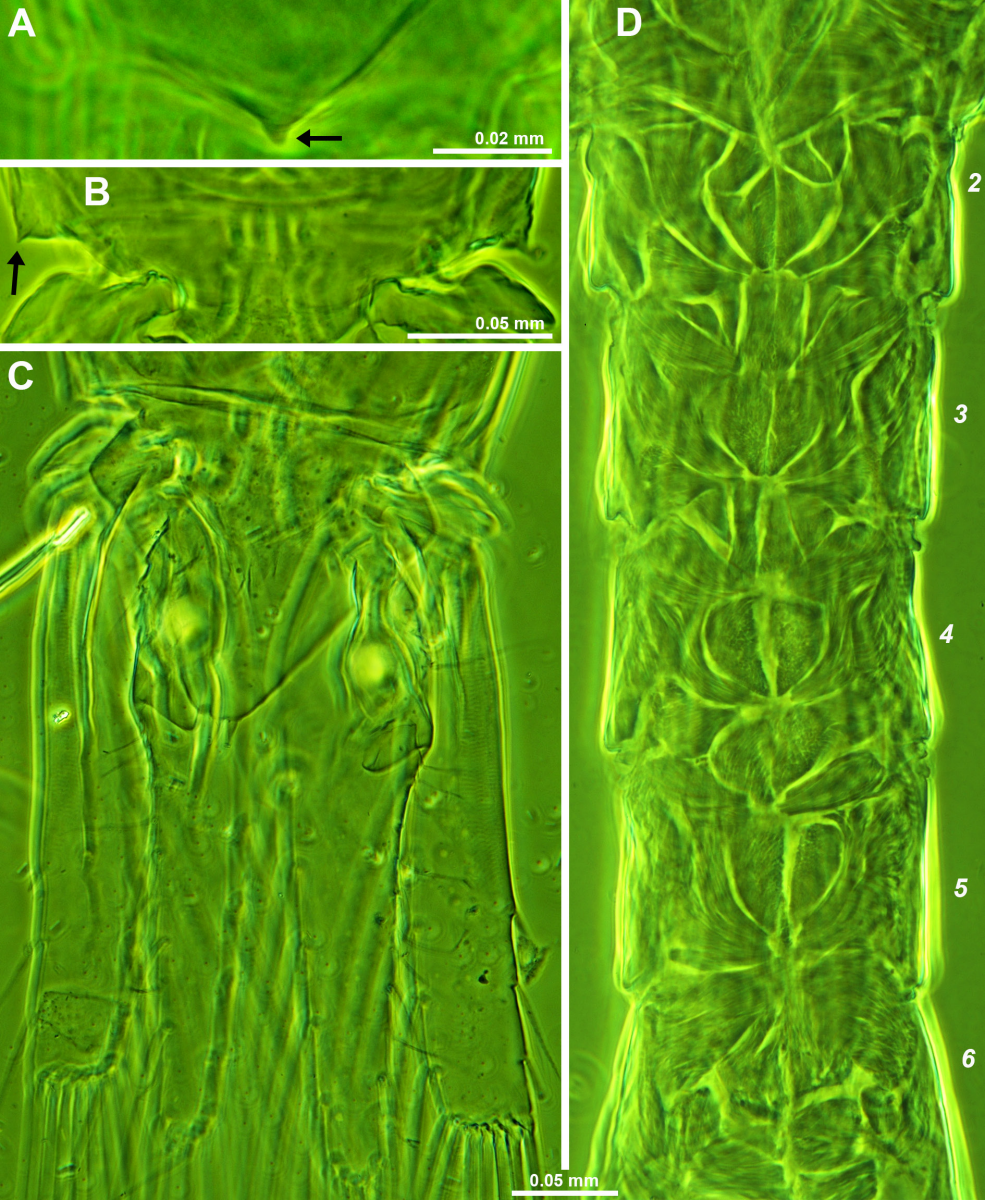
Pleon and tail fan of *Palaumysis simonae* Băcescu & Iliffe adults from submarine cave near Soft Coral Arch, Palau Islands. **A**. Arrow points to apex of right scutellum paracaudale in ♂, lateral; **B**. Posterior margin of pleon in ♀, arrow points to right scutellum paracaudale, ventral view with focus on this scutellum; **C**. Tail fan of same ♀ as before, ventral view with focus on the dorsal telson seen through the bleached tissues; **D**. Pleon in ♂, ventral view with focus on muscle tissue inside pleomeres. A–D, materials expanded and bleached on slides.

***Pleon*** (Figs 15, 16A, B, D). Paramedian pairs of muscle structures as in holotype. Sternites 4, 5 of both sexes with small, obliquely transverse, ventral elevation between pleopods and lateral margin (small arrows in Fig. 15A, B, D). These elevations individually varying from widely blunt (sternite 4 in Fig. 15B) to ending in or bearing a spine-like, caudally facing process (sternite 5 in Fig. 15D). Sternites 1–3 without such processes in both sexes. Pleurite 6 bilaterally shortly in front of caudal margin with apically blunt to acute, triangular scutellum paracaudale in lateral position, laterally (Fig. 16A) only marginally covering part of the telson. Both scutella marking the distolateral edges of the pleon in ventral (arrow in Fig. 16B) as well as dorsal views.

***Pleopods of adult females*** (Fig. 15A). Length (without setae) decreasing from pleopods 1–3 and increasing from 3–5. Pleopods 1–5 with 2–4 small barbed setae on distal half and one smooth seta at or near apex, the latter varying individually in length and from seta-like to strongly spine-like (as in Fig. 15E) in all pleopods. The smooth seta of pleopod 4 in two of 11 available adult females attaining almost the length of the apical seta typical of males.

***Pleopods of adult males*** (Fig. 15B–E). Length decreasing from pleopods 1–3 and increasing from 3 to 4. Pleopod 4 longest, pleopod 1 second longest, pleopod 5 subequal to pleopod 1. Distal half of pleopod 1 bilaterally with total of four or five small barbed setae; apex with one large plumose seta (Fig. 15C), the latter approx. as long or longer (Fig. 15B) than the large apical seta of pleopod 4. Pleopods 2, 3, 5 each with 2–4 small barbed setae on distal half and one smooth seta at or near apex, the latter varying individually in length and from seta-like to strongly spine-like (Fig. 15E) only in these pleopods. Pleopod 4 with one large seta bearing several minute cils near 2/3 length and with an additional small barbed seta at apex; distal half of lateral margin with two or three barbed setae, mesial margin bare (Fig. 15D).

***Tail fan*** (Fig. 16C). The endopod of uropods extends 0.08–0.13× its length beyond exopod and 0.6–0.7× beyond telson; exopod 0.5–0.7× its length beyond telson. Statoliths composed of fluorite, diameter 20–28 µm (*n* = 23 including 15 measurements by Wittmann and Ariani 2019). Exopod 5.2–6.6× longer than wide; proximal 2/3 of lateral margin bare, distal third with three setae increasing in size distally (Fig. 16C). Telson length 0.8–1.2× maximum width or 0.4–0.6× length of pleomere 6. The apical spines are 8–13% telson length.

#### Palaumysis bahamensis

Taxon classificationAnimaliaMysidaMysidae

Pesce & Iliffe, 2002

03D01721-BF83-5DD8-A916-427B1B0BBD44

Figs 12K–M, 17–20

Palaumysis bahamensis Pesce & Iliffe, 2002: 273–275, figs 7–13 (original description); Meland and Willassen 2007: fig. 3 (phylogeny, rRNA sequencing); Chevaldonné et al. 2015: fig. 5 (molecular systematics).

##### Material examined.

Bahamas • 1 ♀ ad. (BL = 1.42 mm; NHMW-ZOO-CR-31426) on slides, from submarine cave (Station 21); this station is ≈260 km SE of the type locality.

##### Types, type locality, and distribution

(Fig. 10J). The types are missing since the 2009 l’Aquila earthquake, when they were probably destroyed (Diana Galassi pers. comm. 2025). Type locality by primary monotypy is Bahamas, South Andros Island, Grassy Cay, submarine cave Atlantis Blue Hole, 23°50.12'N, 77°27.76'W, depth 60–70 m (Pesce and Iliffe 2002; coordinates estimated). Efforts to obtain materials from the type locality repeatedly failed. This mysid is still known only from marine caves in the Bahamas. The present record from a submarine cave at Long Island (Bahamas, Station 21) extends the known range to 23–24°N, 75–77°W, depth 45–70 m.

##### Revised diagnosis.

Essentially based on adult females and on a few characteristics of the only male specimen known from the description by Pesce and Iliffe (2002): *Palaumysis* with mesial antennular flagellum 2-segmented, apical segment 4/5 flagellum length; length of antennal scale 3× maximum width, scale terminally with one long and one short seta, the long seta <1/2 scale length, lateral margin of scale with one short seta; post-cervical lateral margins of carapace with projecting lobes; apex of male pleopod 4 with large smooth seta reaching to pleopod 5, no additional seta.

##### Supplementary description.

Based on single adult female (BL = 1.42 mm) from Long Island, Bahamas. Pesce and Iliffe (2002) reported BL values of 1.9–2.5 mm for five specimens without indication of measurement methods and differentiation between sexes. Pigment not retracted from crystalline bodies of the eye.

**Figure 17.**
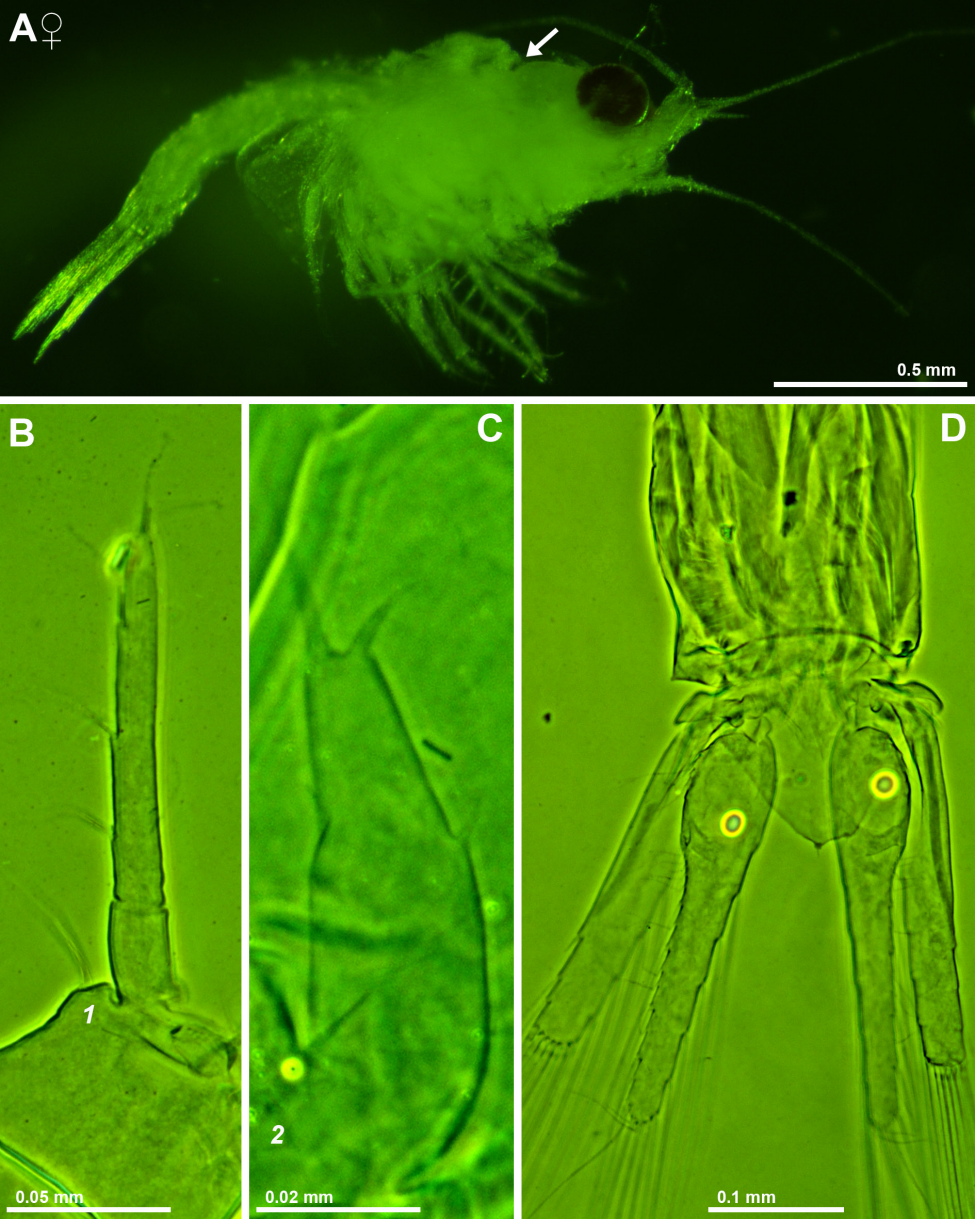
Habitus, part of antennae and tail fan in *Palaumysis bahamensis* Pesce & Iliffe ♀ from submarine cave at Long Island in the Bahamas. **A**. Habitus, arrow points to posterior margin of carapace; **B**. Right mesial flagellum of antennula, dorsal; **C**. Left antennal scale, ventral; **D**. Tail fan, dorsal, terminal spines of telson are damaged.

**Figure 18.**
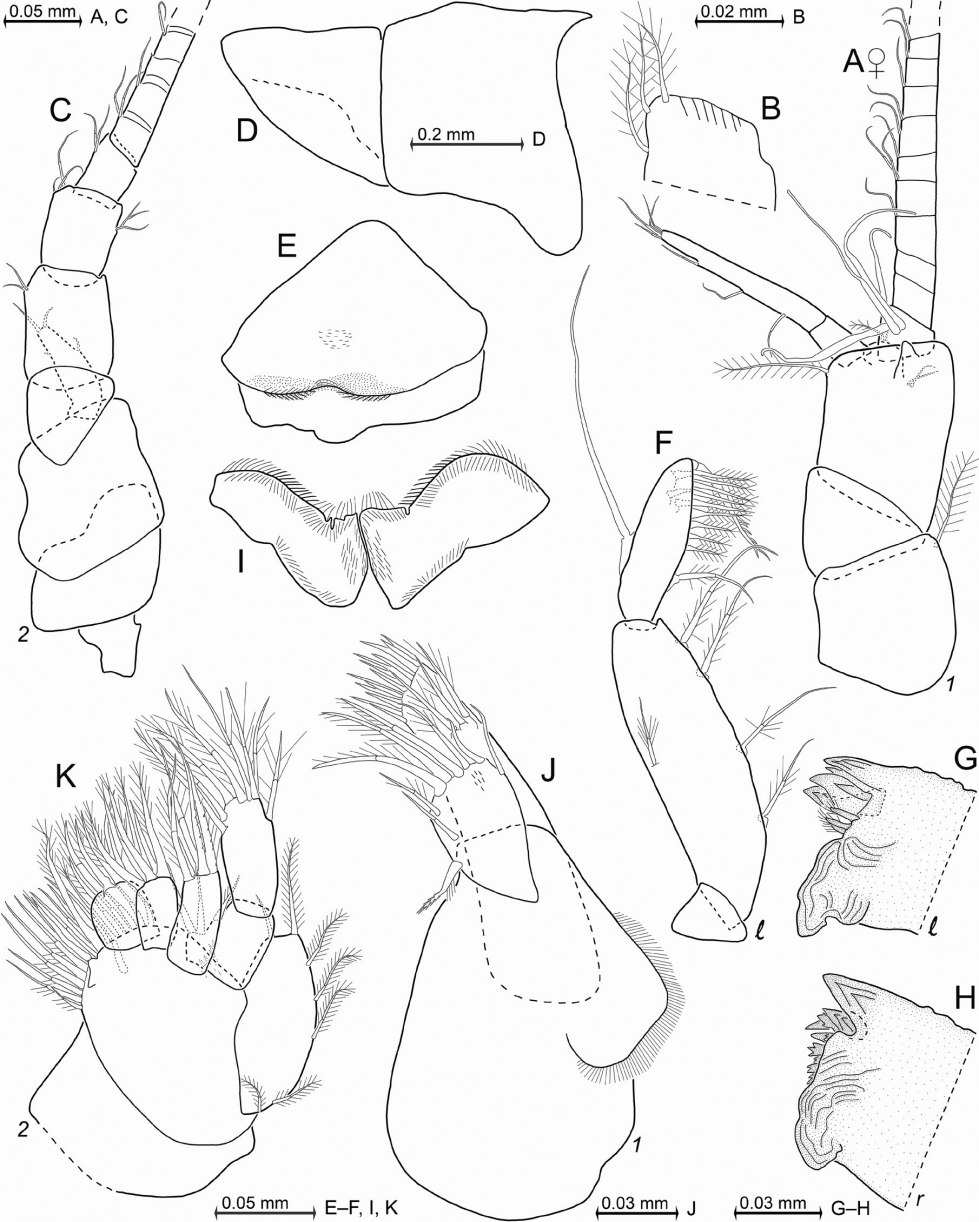
Carapace and cephalic appendages in *Palaumysis bahamensis* Pesce & Iliffe ♀ from submarine cave at Long Island in the Bahamas. **A**. Left antennula, ventral; **B**. Mid-dorsal lobe subterminally on distal segment of right antennular trunk; **C**. Antenna, ventral; **D**. Carapace, lateral; **E**. Labrum, aboral face and foreshortened perspective caudal face; **F**. Mandibular palp, rostral aspect; **G**. Masticatory part of left mandible, caudal; **H**. Masticatory part of right mandible, rostral; **I**. Labium, caudal; **J**. Maxillula, caudal; **K**. Maxilla, caudal.

**Figure 19.**
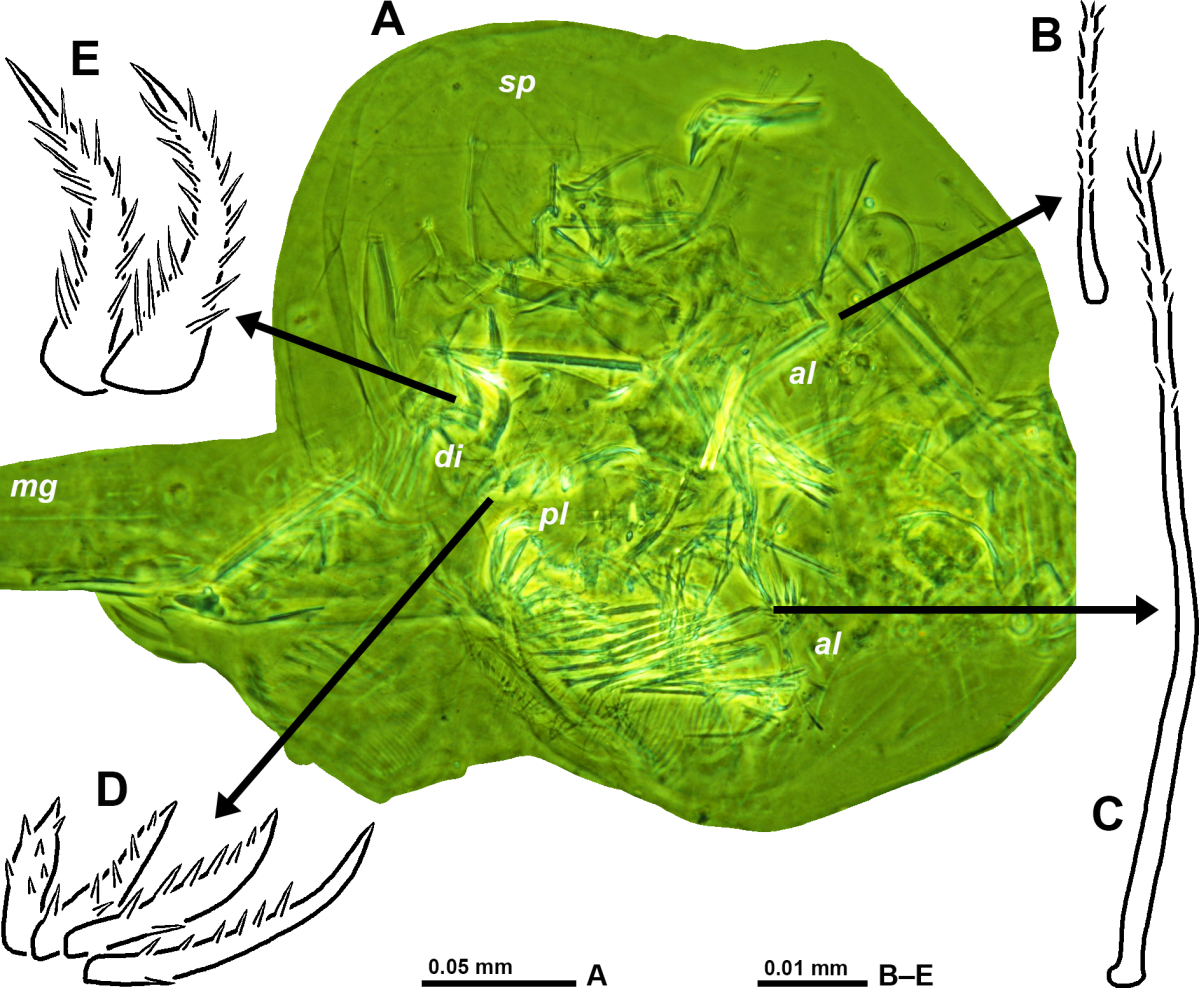
Foregut in *Palaumysis bahamensis* Pesce & Iliffe ♀ from submarine cave at Long Island in the Bahamas. **A**. Foregut with small part of midgut, obliquely lateral, on slide, object artificially separated from background; **B, C**. Spines of anterior lateralia; **D**. Spine group of posterior lateralia; **E**. Spine pair of dorsolateral infoldings. Lower-case labels indicate anterior lateralia (*al*), dorsolateral infolding (*di*), midgut (*mg*), posterior lateralia (*pl*) and storage space (*sp*).

***Cephalothorax*** (Figs 17A–C, 18). Basal segment of the 2-segmented mesial antennular flagellum without setae; terminal segment with three short whip setae along mesial margin; apex with one long and three shorter, widely distributed whip setae (Fig. 17B). Carapace (Figs 17A, 18D) short, leaving ultimate four thoracomeres mid-dorsally exposed; mid-terminal margin marked by arrow in Fig. 17A. Mouthparts as in Fig. 18E–K, foregut as in Fig. 19.

**Figure 20.**
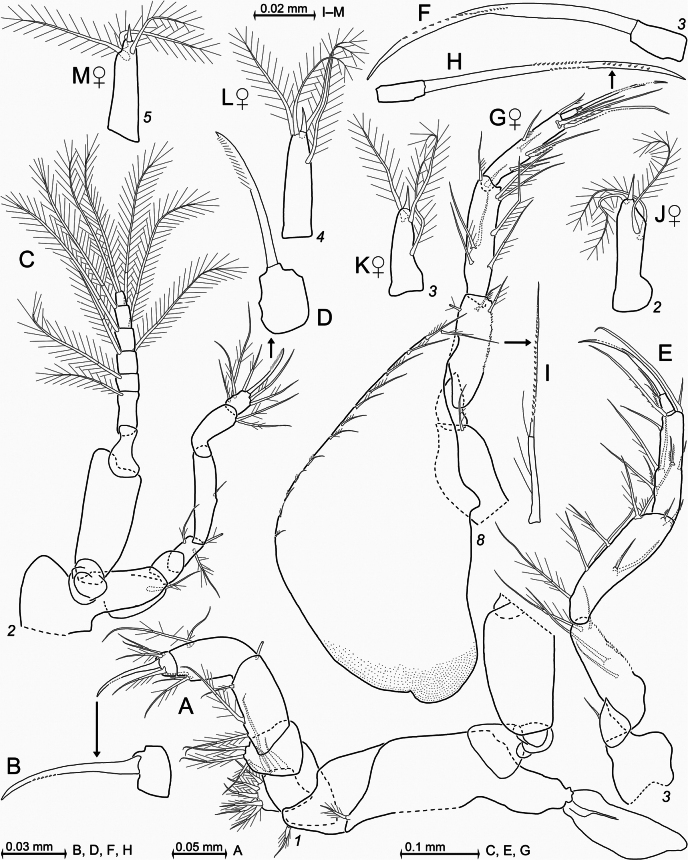
Thoracopods 1–3, 8 and pleopods of *Palaumysis bahamensis* Pesce & Iliffe ♀ from submarine cave at Long Island in the Bahamas. **A**. Thoracic endopod 1 with epipod and basal plate of exopod, caudal; **B**. Dactylus 1; **C**. Thoracopod 2; **D**. Dactylus 2; **E**. Thoracic endopod 3; **F**. Dactylus 3; **G**. Thoracic endopod 8 (rostral) with oostegite 2 (inner = mesial face); **H**. Dactylus 8; **I**. Detail of (G) showing distal modified seta of oostegite; **J–M**. Series of pleopods 2–5, ventral = lateral aspect. **B, D, F, H**. Setae omitted from dactylus.

***Thoracopods*** (Fig. 20A–I). Flagellum of exopods 1–8 with 6, 6 or 7, 7, 6, 6, 6, 7, and 7 segments, respectively. Endopods 3–8 with unsegmented carpopropodus (Fig. 20E, G). Dactyli 1, 2 with normal-sized, weakly bent strong nail (Fig. 20B, D); concave margin of nail 1 weakly microserrated on distal third by stiff cils (Fig. 20B), nail 2 with longer stiff cils along distal half (Fig. 20D). Nails 3–8 long, needle-shaped, weakly bent; the needles microserrated by minute teeth along median to subapical portions (Fig. 20F, H). Thoracic endopods 3–8 with minute triangular scales on ischium and merus as in *P. antillensis* sp. nov. (Fig. 26D, E).

***Marsupium*** (Figs 17A, 20G, 20I). Oostegites 1, 2 from thoracopods 7, 8 representing simple unfolded plates. Lower margin (to the left in Fig. 20G) with series of comparatively few whip setae furnished with a few barbs at handle and minute denticles (spines) along flagellum (Fig. 20I); no other setae modifications.

***Pleon***. As in *P. simonae* (Fig. 16A, B), each pleurite 6 of *P. bahamensis* bears an apically blunt to acute, triangular scutellum paracaudale. In dorsal view the scutella mark the caudolateral edges of the pleon (Fig. 17D).

***Pleopods*** (Fig. 20J–M). Length (without setae) decreasing from pleopods 1 to 3 and increasing from 3 to 4, pleopod 5 subequal to pleopod 4. Pleopods 1–5 with 2–4 small barbed setae on distal half and one smooth seta at or near apex. The smooth seta strongly spine-like as in Fig. 20J–M. The smooth seta of pleopod 4 approx. as long as in pleopods 2, 3.

***Tail fan*** (Fig. 17D). The endopod of uropods extends 0.1–0.2× its length beyond exopod and 0.7–0.8× beyond telson; exopod 0.5–0.7× its length beyond telson. Exopod 7.0× longer than wide; proximal 2/3 of lateral margin bare, distal third with three setae increasing in length distally. Diameter of the spherical fluorite statoliths is 18 μm (*n* = 2). Telson 1/2 as long as pleomere 6, its length 1.1× maximum width.

#### Palaumysis antillensis sp. nov.

Taxon classificationAnimaliaMysidaMysidae

AB007AB1-B0E2-5D0F-90F6-ECFC82D437FD

https://zoobank.org/FCC791E0-3C6C-4E0B-B5BF-763BE469744A

Figs 12H–J, 21–28

##### Type material.

Lesser Antilles — **SW-Bequia** • ***holotype***, ♂ ad. (BL = 1.43 mm; NHMW-ZOO-CR-31412) from Full Moon Cave (Station 4) • ***allotype***, egg-bearing ♀ (BL = 1.60 mm; MNHN-IU-2025-2833), sampling data as for holotype • ***paratypes***, 9 ♀♀ ad., 1 ♀ subad., 8 ♂♂ ad., 11 imm., 1 juv. (MNHN-IU-2025-2834), sampling data as for holotype • ***paratypes***, 6 ♀♀ ad., 11 ♂♂ ad., 13 imm. (NHMW-ZOO-CR-31425), sampling data as for holotype • ***paratypes***, 10 ♂♂ ad. (BL = 1.27–1.67 mm), 10 ♀♀ ad. (BL = 1.60–1.87 mm) (MINGA MYS 457), sampling data as for holotype • ***paratypes***, 9 ♀♀ ad., 8 ♂♂ ad., 13 imm. (ZMH-K-066759), sampling data as for holotype — **Guadeloupe** • ***paratypes***, 8 ♀♀ ad., 10 ♂♂ ad., 12 imm. (MNHN-IU-2025-2835) from Cathedral Cave (Station 13) • ***paratypes***, 9 ♀♀ ad., 2 ♀ subad., 5 ♂♂ ad., 9 imm., 5 juv. (NHMW-ZOO-CR-31413), sampling data as for preceding • ***paratypes***, 16 ♀♀ ad., 1 ♀ subad., 7 ♂♂ ad., 6 imm. (ZMH-K-066760), sampling data as for preceding.

##### Other material.

Cuba • 1 ♀ ad. (BL = 1.87 mm) from the anchialine El Brinco Cave (Station 23).

##### Diagnosis.

*Palaumysis* with mesial antennular flagellum 3- to 5-segmented, apical segment 1/5–1/6 flagellum length; length of antennal scale 3–4× maximum width, scale terminally with one long and one or two short setae, the long seta 0.6–1.0× scale length, lateral margin of scale with none or one short seta; post-cervical lateral margin of carapace with projecting lobes; pleopod 1 with only moderately-sized setae in both sexes, no large plumose seta; apex of male pleopod 4 with one large and one short seta, the former reaching to or beyond pleopod 5 and bearing bilateral series of small cils from submedian to subapical portions, lateral margin of pleopod 4 with 0–3 short setae.

##### Description.

Adult ♀♀ with BL in the range of 1.53–1.92 mm (*n* = 119), ♂♂ with BL of 1.05–1.67 mm (*n* = 129) in the material from the Lesser Antilles. Cephalothorax comprises 34–43% BL, pleon (without telson) 46–56%, telson 5–8%, carapace (without rostrum) 22–33%, and rostrum 3–6%. Clypeus transversely ellipsoidal, wider than long, with short, triangular mid-rostral protrusion (Figs 22, 24D). Thoracic sternites without mid-ventral processes in both sexes, no spines, no setae.

**Figure 21.**
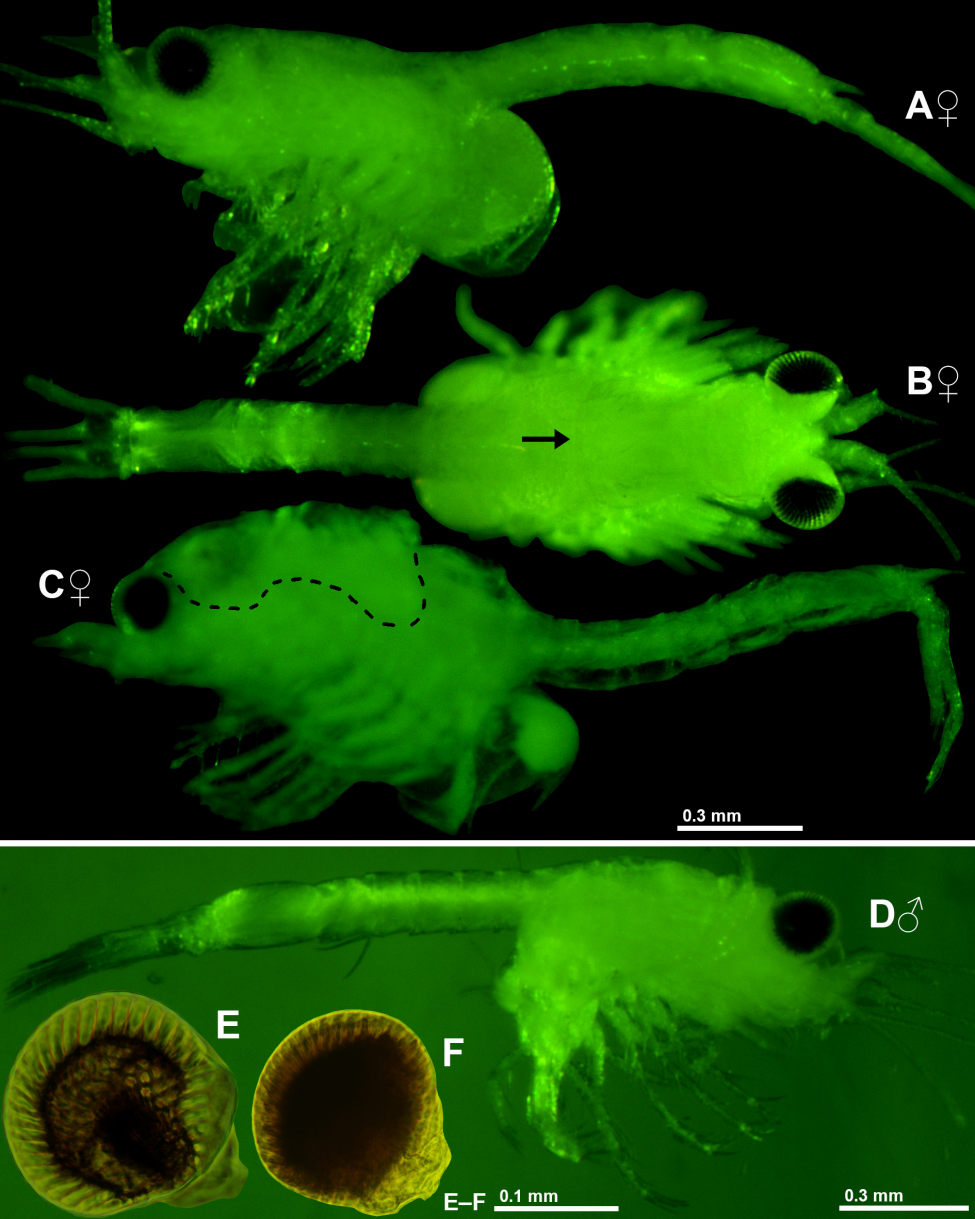
Habitus and distribution of corneal pigment in adults of *Palaumysis antillensis* sp. nov. **A**. Allotype, egg-bearing ♀ from Full Moon Cave in Bequia, lateral; **B**. Paratype, ♀ from Full Moon Cave, dorsal, arrow points to caudal margin of carapace; **C**. Non-type incubating ♀ from El Brinco Cave in Cuba, lateral, dashed line enhances the contour of carapace; **D**. Holotype, ♂ from Full Moon Cave, lateral; **E, F**. Examples of pigment distribution in eyes. **A–C, E, F**. Objects artificially separated from background.

**Figure 22.**
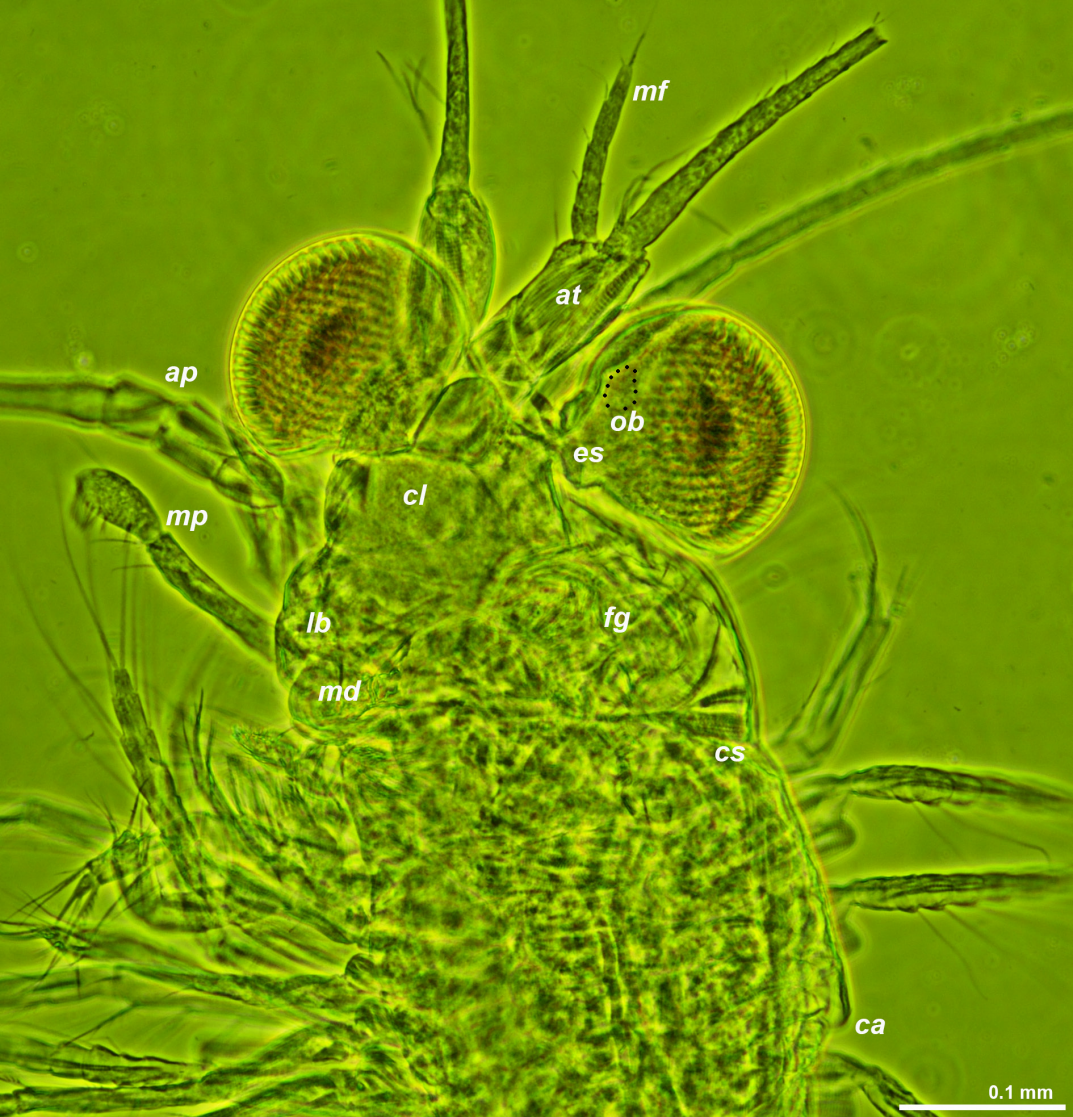
Anterior body region of *Palaumysis antillensis* sp. nov. paratype immature ♀ (BL = 1.03 mm) from Full Moon Cave in Bequia; specimen expanded and bleached on slide, oblique dorsal view with focus on ventral structures seen through the artificially transparent body; contour of OB enhanced by dots. Lower-case labels indicate antennal peduncle (*ap*), antennular trunk (*at*), caudal margin of carapace (*ca*), clypeus (*cl*), cervical sulcus (*cs*), eyestalk (*es*), foregut (*fg*), labrum (*lb*), mandible (*md*), mesial flagellum of antennula (*mf*), mandibular palp (*mp*) and Organ of Bellonci (*ob*).

**Figure 23.**
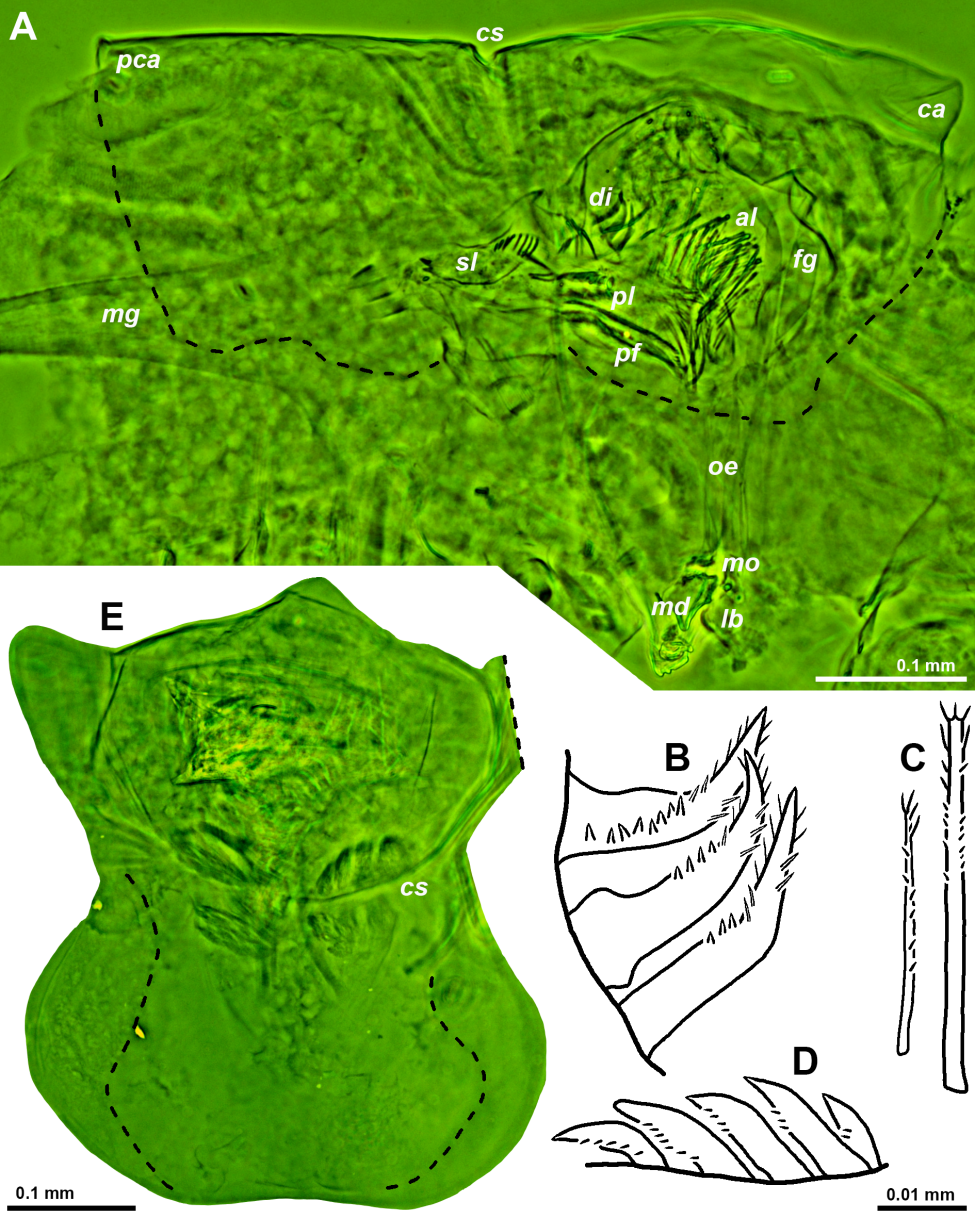
Anterior alimentary tract and carapace in *Palaumysis antillensis* sp. nov. adult paratypes from **A–D**. Cathedral Cave in Guadeloupe and **E**. Full Moon Cave in Bequia. **A**. Lateral view on carapace and anterior half of alimentary tract in ♂, dashed lines enhance contours of carapace, material somewhat malformed by pressure exerted by cover glass, lower-case labels indicate anterior lateralia (*al*), carapace (*ca*), cervical sulcus (*cs*), dorsolateral infolding (*di*), foregut (*fg*), labrum (*lb*), mandible (*md*), midgut (*mg*), mouth opening (*mo*), esophagus (*oe*), post-cervical part of carapace (*pca*), primary cardiac filter (*pf*), posterior lateralia (*pl*) and superolaterale (*sl*); **B**. Spines of dorsolateral infoldings (*di*); **C**. Spines of anterior lateralia (*al*); **D**. Spines of posterior lateralia (*pl*); **E**. Carapace expanded on slide, ♀, dorsal view, underneath lying foregut plus part of midgut largely out of focus, lower dashed lines enhance proximal margin of projecting portions of carapace, object artificially separated from background.

***Carapace*** (Figs 21B, 21C, 22, 23A, 23E). Rostrum as long as 0.3–0.6× terminal segment of antennular trunk in dorsal view. The cervical sulcus forms a deep transverse incision (Fig. 23A). The length of the post-cervical part of the carapace is 0.9–1.2× the cervical part measured without rostrum. The posterior part with comparatively large, projecting lateral lobes (Fig. 23E). Posterior margin slightly concave (Fig. 23E) to slightly convex (Fig. 21B), leaving ultimate four or five thoracomeres mid-dorsally exposed (in adults vs 1–4 in juveniles and immatures).

***Eyes*** (Figs 21E, 21F, 22). Eye length 0.12–0.22 mm (*n* = 20), this is 8–15% BL. Cornea calotte-shaped in dorsal view, diameter of the calotte 0.16–0.39 mm, length 0.07–0.17 mm, this is 0.6–1.3× length of eyestalk (cornea not included). Cornea circular (Fig. 21D) to weakly oval (Fig. 21A) in lateral view, length 0.9–1.4× maximum width. A small number of specimens in the present material appear light-adapted based on the very few pigments in the crystalline zone (Fig. 21E) of the cornea; most of the other specimens appear dark-adapted by displaying more pigment between the crystalline cones (Fig. 21F) upon external inspection (for light adaptation by slow pigment migration see Land 2004). OB completely internal, located at half-length of mesial margin of eyestalk, ellipsoidal to oviform with length 26–33 µm and width 15–22 µm (*n* = 8; Fig. 22).

**Figure 24.**
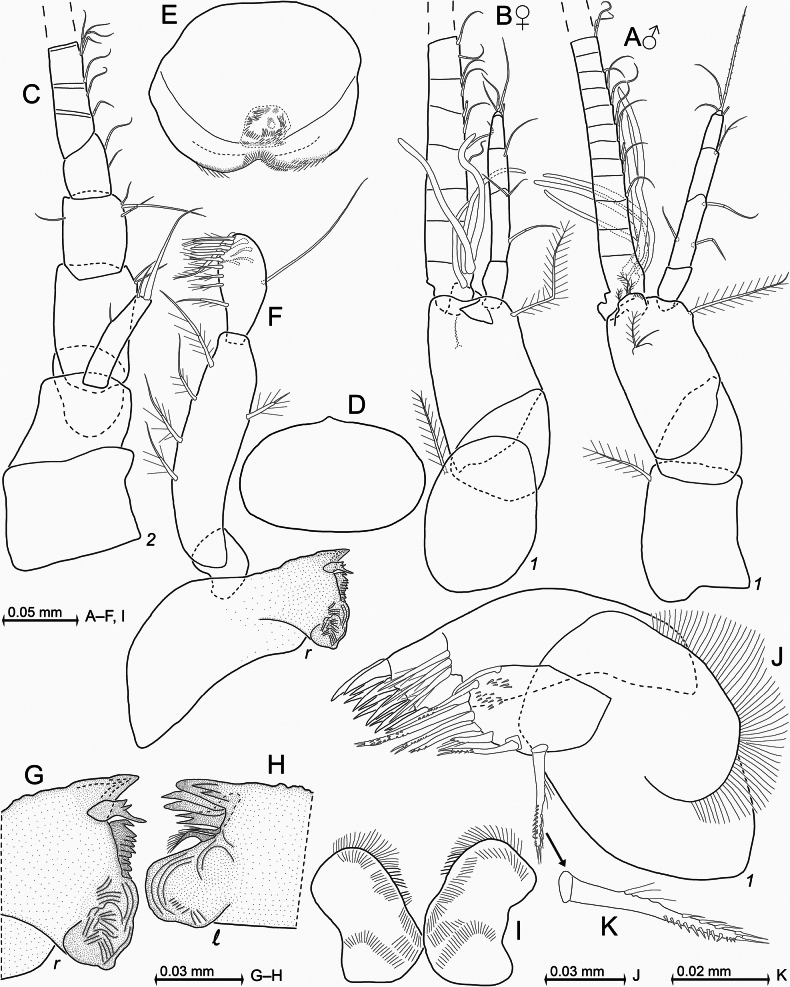
Cephalic appendages in *Palaumysis antillensis* sp. nov. adult paratypes from **A, D**. Cathedral Cave in Guadeloupe; **B, C, E–K**. Full Moon Cave in Bequia; **A**. Left male antennula, dorsal; **B**. Right female antennula, ventral; **C**. Left antenna, ♀, dorsal; **D**. Clypeus; **E**. Labrum, oral face; **F**. Right mandible with palpus, caudal aspect; **G, H**. Masticatory parts of right (**G**) and left (**H**) mandibles, caudal; **I**. Labium, caudal; **J**. Maxillula, caudal; **K**. Detail of (**J**) showing modified seta of endite.

***Antennulae*** (Figs 22, 24A, B). Antennular trunk non-dimorphic, 3-segmented, with few setae, no spines, no teeth. Trunk extends 22–50% its length beyond eyes in normal orientation (Fig. 21B). Measured along dorsal midline, the basal segment is 30–43% of trunk length, median segment 10–18%, and terminal segment 47–56%. Basal segment distolaterally with only one plumose seta, no apophysis. Median segment shows oblique segmental borders with both flanking segments in the sagittal plane, no apophysis, no setae. Terminal segment 1.6–2.1× as long as wide; distolateral edge with a large plumose seta. Only this segment mid-dorsally with a subterminal setose apophysis bearing three or four small barbed setae (Fig. 24A). The rudimentary mesial antennular flagellum 3- to 5-segmented; if 5-segmented with second segment shortest and fourth segment longest, otherwise (Fig. 24A) with first segment shortest and second segment longest. Apical segment 1/5–1/6 flagellum length, its apex with long seta accompanied by 2–4 short setae. The lateral antennular flagellum normal, its basal segment with 3–5 long, apically blunt aesthetascs.

***Antennae*** (Fig. 24C). Antennal sympod 2-segmented, simple, no spines, no setae. Peduncle 3-segmented, basal segment contributes 26–34% to total length of peduncle, median segment 36–43%, and terminal segment 28–33%. Antennal scale vestigial, unsegmented, length is 3.1–4.7× maximum width. Terminal margin truncate, with large bare seta subterminally to terminally accompanied by a small one; lateral margin with none or one small seta. Scale not reaching to distal margin of median segment of peduncle.

**Figure 25.**
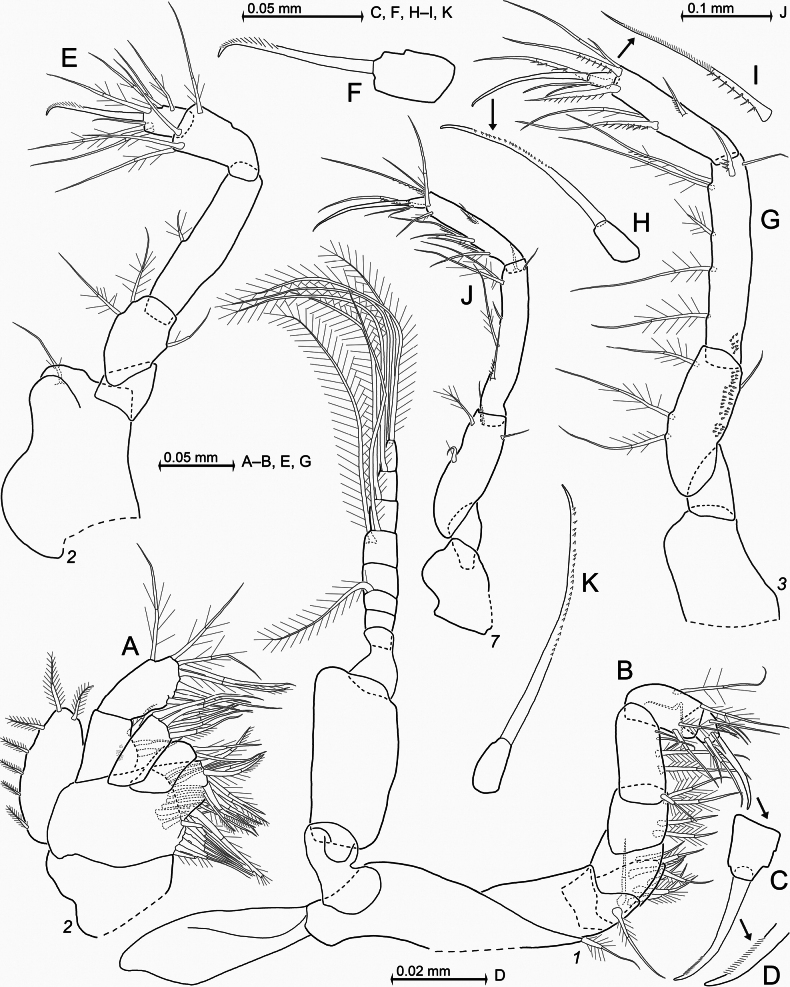
Maxilla and thoracopods 1–3, 7 in *Palaumysis antillensis* sp. nov. male paratypes from **A**. Full Moon Cave in Bequia; **B–K**. Cathedral Cave in Guadeloupe. **A**. Maxilla, caudal aspect; **B**. Thoracopod 1, caudal; **C**. Detail of (**B**) showing dactylus 1 with nail; **D**. Detail of (**C**) showing tip of nail; **E**. Thoracic endopod 2; **F**. Detail of (**E**) showing dactylus 2 with nail; **G**. Thoracic endopod 3; **H, I**. Details of (**G**) showing dactylus 3 with nail (**H**) and paradactylar seta (**I**); **J**. Thoracic endopod 7 (note smaller scale with respect to panels **A, B, E, G**); **K**. Detail of (**J**) showing dactylus 7 with nail. **C, F, H, K**. Setae omitted from dactylus.

***Mouthparts*** (Figs 23A, 24E–K, 25A). Labrum (Fig. 24E) rostrally well rounded; caudally weakly rugged and with small, stiff bristles; fields of setae on caudal and oral faces. Mandibles (Fig. 24F–H). Proximal segment of palp 10–13% palp length, no setae. Length of median segment 3.1–4.1× its maximum width and 55–65% palp length. Distal half of mesial margin of median segment with none or one sparsely barbed whip seta, lateral margin along distal 3/4 with four setae of that type, transverse terminal margin without seta (Fig. 24F). Terminal segment well setose, 26–31% palp length; its mesial margin with long smooth seta (longer than the segment). Pars molaris with moderately developed grinding surface. Left and right mandibles differ in armature with teeth and spines (Fig. 24H vs Fig. 24G). Pars incisiva of left mandible with four or five teeth, digitus mobilis with 2–4 teeth, and pars centralis with two or three spines bearing stiff bristles. Pars incisiva of right mandible with three or four teeth, digitus mobilis with three or four teeth, and pars centralis with complex of 7–10 bare teeth densely set on a large common basis. Labium (Fig. 24I) normal, comprising two hairy lobes with stiff bristles on part of mesial face, no spines.

***Foregut*** (Fig. 23A–D). Gross structure as described by Wittmann and Abed-Navandi (2019) for *Heteromysis domusmaris* Wittmann & Abed-Navandi, 2019. Lateralia anteriorly with apically pronged spines (Fig. 23C), each with small denticles along distal 1/2–2/3; more caudally with group of four or five unilaterally weakly serrated spines (Fig. 23D). Dorsolateral infoldings with group formed by two or three long spines bearing unequally strong teeth along distal 1/2–4/5 of shaft (Fig. 23B). Four of eight dissected foreguts were (almost) empty; the remaining were filled to ≈10–30% with mostly unidentified organic material (detritus) and mineral particles.

***Maxillula*** (Fig. 24J, K) normal; distal segment terminally with 10–12 bare spines; subterminally with three setae bearing long barbs on their distal half; no pores detected. Endite of maxillula terminally with four or five strong, distally spiny setae proximally accompanied by one barbed seta; oral margin with two distally spiny setae, the most proximal one modified as in Fig. 24K and pointing to the mouth field.

***Maxilla*** (Fig. 25A) normal; endopod with bare lateral margin, not counting a single seta subterminally on the distal segment; this segment 1.6–1.8× as long as maximum width; proximal segment mesially with three basally barbed whip setae with thick handles. Lateral margin of exopod with five or six plumose whip setae, terminal margin with two plumose whip setae longer than the lateral setae.

**Figure 26.**
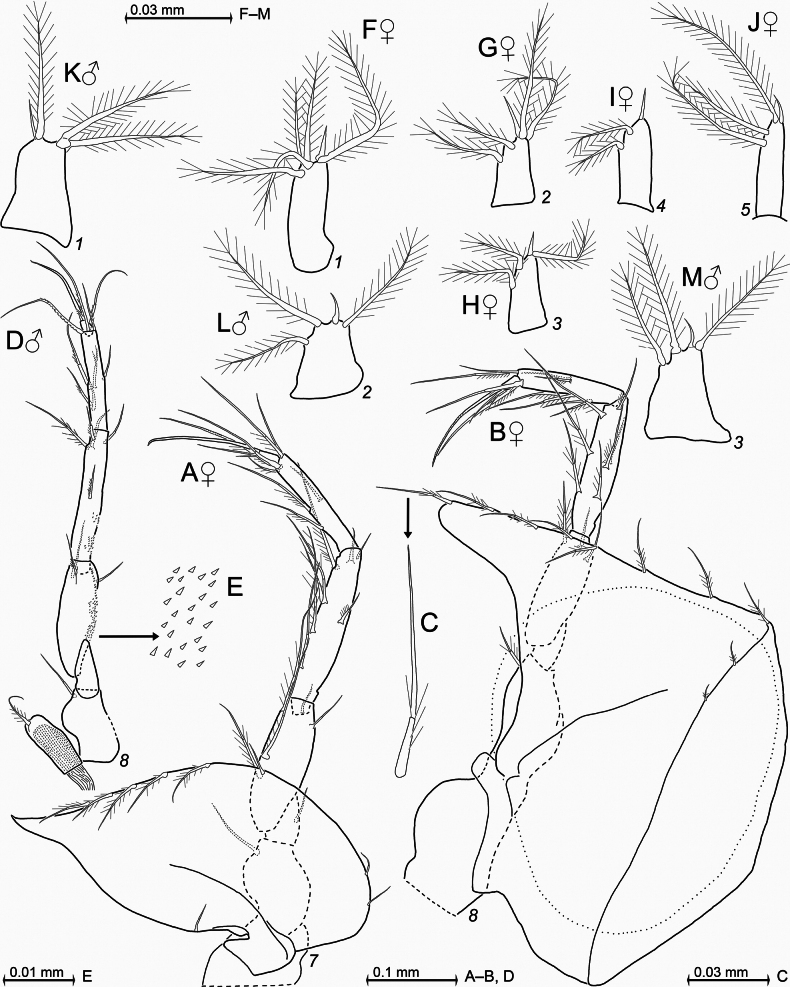
Thoracopods 7, 8 and part of pleopods in *Palaumysis antillensis* sp. nov. adult paratypes from **A–C, K–M**. Full Moon Cave in Bequia and **D–J**. Cathedral Cave in Guadeloupe. **A**. Thoracic endopod 7 (caudal aspect) with oostegite 1 (inner face); **B**. Thoracic endopod 8 (caudal) with oostegite 2 (inner face), dotted line indicates size and position of the respective egg; **C**. Detail of (B) showing weakly modified seta; **D**. Thoracic endopod 8 with penis; **E**. Detail of (**D**) showing examples of scales on ischium and merus; **F–J**. Series of female pleopods 1–5; **K–M**. Series of male pleopods 1–3.

***Thoracopods*** (general, Figs 25B–K, 26A–E). Both sexes with flagellum of exopods 1–8 with 6 or 7, 7, 7, 6 or 7, 7, 7, 7, and 6 or 7 segments, respectively. Basal plates expanded, 1.6–2.4× as long as wide, distolateral corner well rounded (Fig. 25B). Thoracopod 1 with leaf-like, smooth epipod. Total length of endopods increases in series of endopods 1–7, while endopod 8 is shorter than endopods 5–7. Endopods 3–8 with unsegmented carpopropodus, with minute triangular scales (Fig. 26E) on rostral face near and on lateral margin of ischium and merus (Figs 25G, 26D). Endopods 3–4 each with three paradactylar whip setae, endopods 5–8 with two or three such setae. Flagellum in most of these setae furnished with minute cils, while handle with normal-sized cils (barbs). Cils of the handle spine-like, each accompanied by a tiny cil (Fig. 25I) in some paradactylar setae only in endopod 3. Dactylus 1 (Fig. 25C) with strong, weakly bent nail; distal fourth of the inner (concave) margin microserrated by minute stiff cils except for a short, most apical bare portion in this nail (Fig. 25D). Dactylus 2 (Fig. 25F) with strong nail bent near apex; distal third of this nail with outer (convex) margin microserrated by minute stiff cils which are longer than those of nail 1. Dactyli 3–8 (Fig. 25H, K) with long, slender, needle-shaped, weakly bent nail, distal 1/2–2/3 of the outer (convex) margin of this nail microserrated by minute teeth. Length increases from nail 1 to nail 7 (series of nails 1–3, 7 in Fig. 25C, F, H, K), nail 8 shorter than nail 7. Endopod 3 is 1/4 BL (Fig. 25G). When stretched anteriorly, endopod 8 reaches to the eyes, when stretched posteriorly to pleopod 5 or shortly beyond.

***Maxillipeds*** (Fig. 25B–F). Tip of coxa of first maxilliped (thoracic endopod 1, Fig. 25B–D) with one short whip seta with barbed handle. Basis with large, prominent endite that is densely setose on and near its mesial margin. Ischium and merus without endites. Basis of second maxilliped (thoracic endopod 2, Fig. 25E, F) with mesially projecting endite bearing one large, sparsely barbed seta. Combined praeischium plus ischium are 0.6–0.8× length of merus, carpopropodus plus dactylus 0.7–0.8× merus. Dactylus large, with comparatively meager brush formed by few basally barbed whip setae and even fewer bare setae.

***Marsupium*** (Fig. 26A–C). Oostegite 1 (Fig. 26A) arising from thoracopod 7 represents a simple unfolded plate; lower margin of rostral half with series of whip setae with barbed handles; caudal half furnished with only few small whip setae. Oostegite 2 (Fig. 26B) from thoracopod 8 with inward (mesially) folded caudal portion forming the caudal wall of the marsupial chamber; lower margin with series of barbed whip setae (Fig. 26C) decreasing in length caudally. No setae on outer face of marsupium.

***Penes*** (Fig. 26D) simple, 0.5–0.6× length of ischium 8. Terminally well rounded, only one barbed seta on distal third.

**Figure 27.**
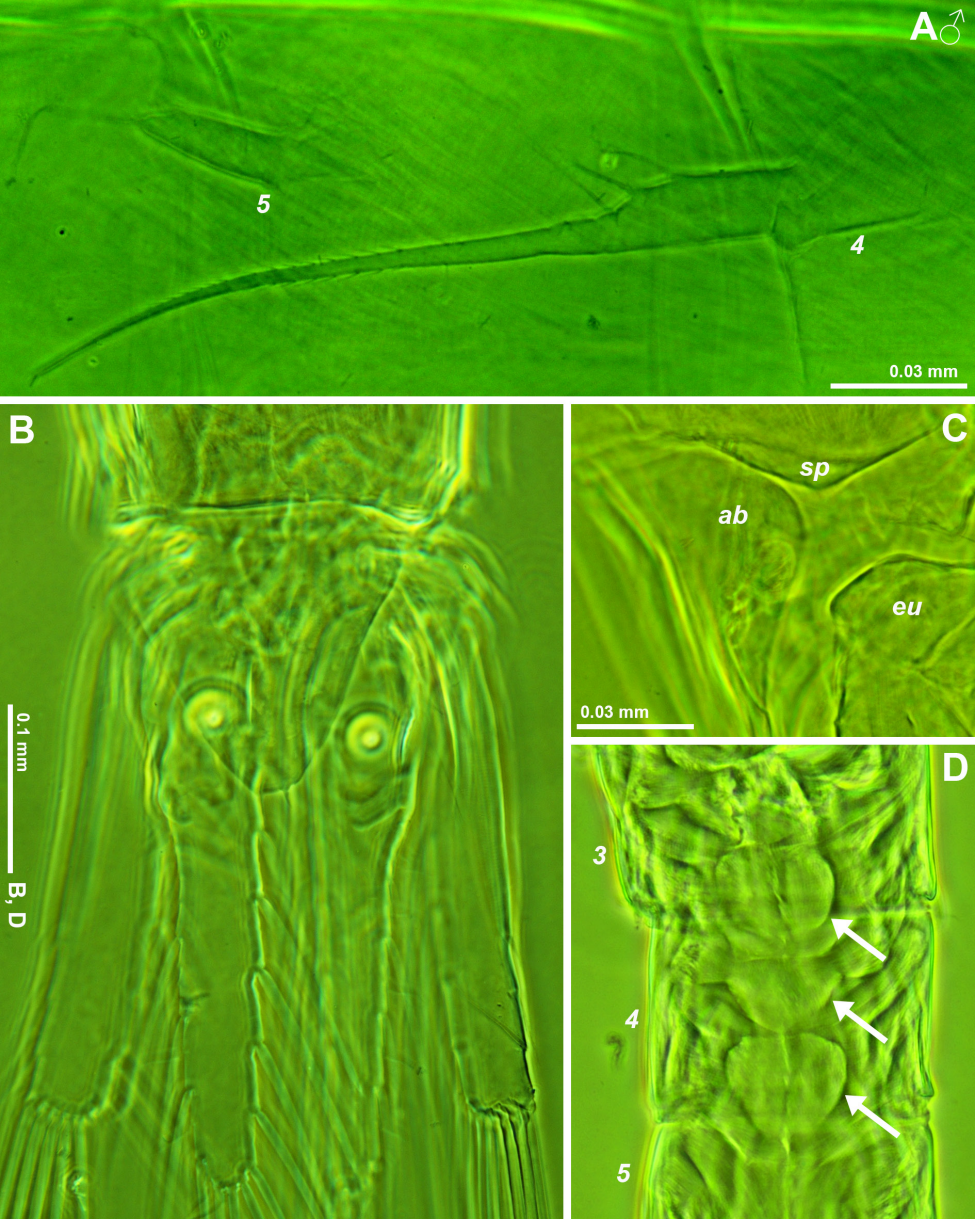
Pleon and tail fan in *Palaumysis antillensis* sp. nov. adult paratypes from Full Moon Cave in Bequia. **A**. Male pleopods 4, 5 in loco, ventral view on sternite 5 flanked by part of sternites 4, 6; **B**. Tail fan in ♀, dorsal; **C**. Scutellum paracaudale (*sp*), anal bulge (*ab*) of telson and basis of endopod of uropod (*eu*) in ♂, right lateral aspect; **D**. Pleomeres 3, 4, and part of pleomere 5 in ♀, dorsal view with focus on tissue inside pleomeres, arrows point to paramedian pairs of muscle structures visible as near-pentagonal images.

***Pleon and pleopods*** (Figs 26F–M, 27A, 27D). Pleomeres 1–5 showing 0.4, 0.3–0.4, 0.3–0.4, 0.3–0.4 and 0.4–0.5 times the length of pleomere 6, respectively. Paramedian pairs of muscle structures, visible as near-pentagonal images (arrows in Fig. 27D), located in pleomeres 2–4 between neuronal cord and intestine. With some variation, length (without setae) increases in order of pleopods 2–5, 1 in females (Fig. 26F–J) and in order of pleopods 2, 3, 1, (5 ≤ 4) in males (Figs 26K–M, 27A). Length of the respective pleopods 1–3, 5 approx. the same in both sexes, while pleopod 4 is mostly larger in males than in females. All pleopods of both sexes with 2–5 barbed setae (plus other setae). Apex of pleopods 1–3, 5 in both sexes and of pleopod 4 in most females with a short spine-like, bare seta. Apex of male pleopod 4 with none or one short seta in addition to the large modified seta, the latter reaching to or beyond pleopod 5 (Fig. 27A); lateral margin of pleopod 4 with 0–3 short setae. A large seta at apex of pleopod 4 also found in many immatures and in three of 25 adult females inspected in this respect. Strong size differences of the apical seta between right and left pleopods 4 were also recorded. The pleopods alone clearly do not yield sufficient data for firm distinction between sexes in this species.

***Tail fan*** (Fig. 27B, C). Scutellum paracaudale short, triangular, tip narrowly rounded; not (Fig. 27C) or only marginally, laterally covering part of telson. The exopod of uropods measures 0.9–1.1 (mostly 0.9) × length of endopod. In part due to inserting more caudally, the endopod extends by 0.04–0.2× its length beyond exopod and 0.6–0.8× beyond telson; exopod 0.5–0.7× its length beyond telson. Exopod 5.3–6.8× longer than wide; proximal 2/3 of lateral margin bare, distal third with 3–4 (mostly 3) setae increasing in size distally (Fig. 27B). Diameter of the spherical fluorite statoliths is 15–28 μm (*n* = 20). Telson length 0.9–1.2× its maximum width or 0.4–0.6× length of pleomere 6. Telson length 2.1–4.2× maximum thickness at anal bulge (Fig. 27C), rendering the telson clearly wedge-shaped in lateral view. The apical spines are 4–8% telson length.

##### Notes on the Cuban material.

The only specimen, an adult female, available from Cuba was completely dissected. Mesial antennular flagellum 4-segmented, apical segment 1/6 flagellum length; length of antennal scale 3.3× maximum width, terminal margin of scale with one long and one short seta, the long seta 0.6× scale length, lateral margin of scale with one additional short seta; post-cervical lateral margin of carapace with projecting lobe; pleopod 1 with three setae, the largest seta 0.9× pleopod length. The length of the longest seta at the terminal margin of the antennal scale is at the lower diagnostic limit of *P. antillensis* sp. nov., remaining features are well within limits. Numbers of segments and size of the apical segment of the mesial antennular flagellum exclude *P. bahamensis*.

##### DNA data.

Only two DNA sequences are available for *Palaumysis* in GenBank, at the 18S nuclear locus: one for *P. bahamensis* (AM422517) collected at Long Island, Bahamas and one for *P. simonae* (AM422516) collected at Palau, Micronesia, both originating from Meland and Willassen (2007). The two Lesser Antilles populations of *P. antillensis* sp. nov. sampled here (Bequia and Guadeloupe) are geographically ≈400 km apart and ≈2000 km from the Cuban occurrence reported here. The Antillean populations are also at least 1500 km away from the known populations of *P. bahamensis*. The Bequia and Guadeloupe 18S sequences (GenBank PV990185–PV990200) obtained (from 16 individuals) are all identical with the total sequence length of 813 bp. Unfortunately, no DNA data were available for the more distant Cuban population.

When aligning the Antilles 18S sequences with published ones, they are 0.37% divergent from *P. bahamensis*. As reflected in divergence between *P. bahamensis* and *P. simonae*, which is 1.24%, 18S must be considered a conserved gene in *Palaumysis* as in most other mysids (Lejeusne and Chevaldonné 2006; Rastorgueff et al. 2014). The divergence data corresponds well with the minor morphological differences noted here between the W-Atlantic sister species *P. bahamensis* and *P. antillensis* sp. nov. compared with the differences between this W-Atlantic pair and the W-Pacific *P. simonae*.

##### Derivatio nominis.

The species name of *P. antillensis* sp. nov. is a Latinized adjective with a feminine ending, referring to the occurrence in islands of the Lesser Antilles.

##### Distribution and habitat

**Figure 28.**
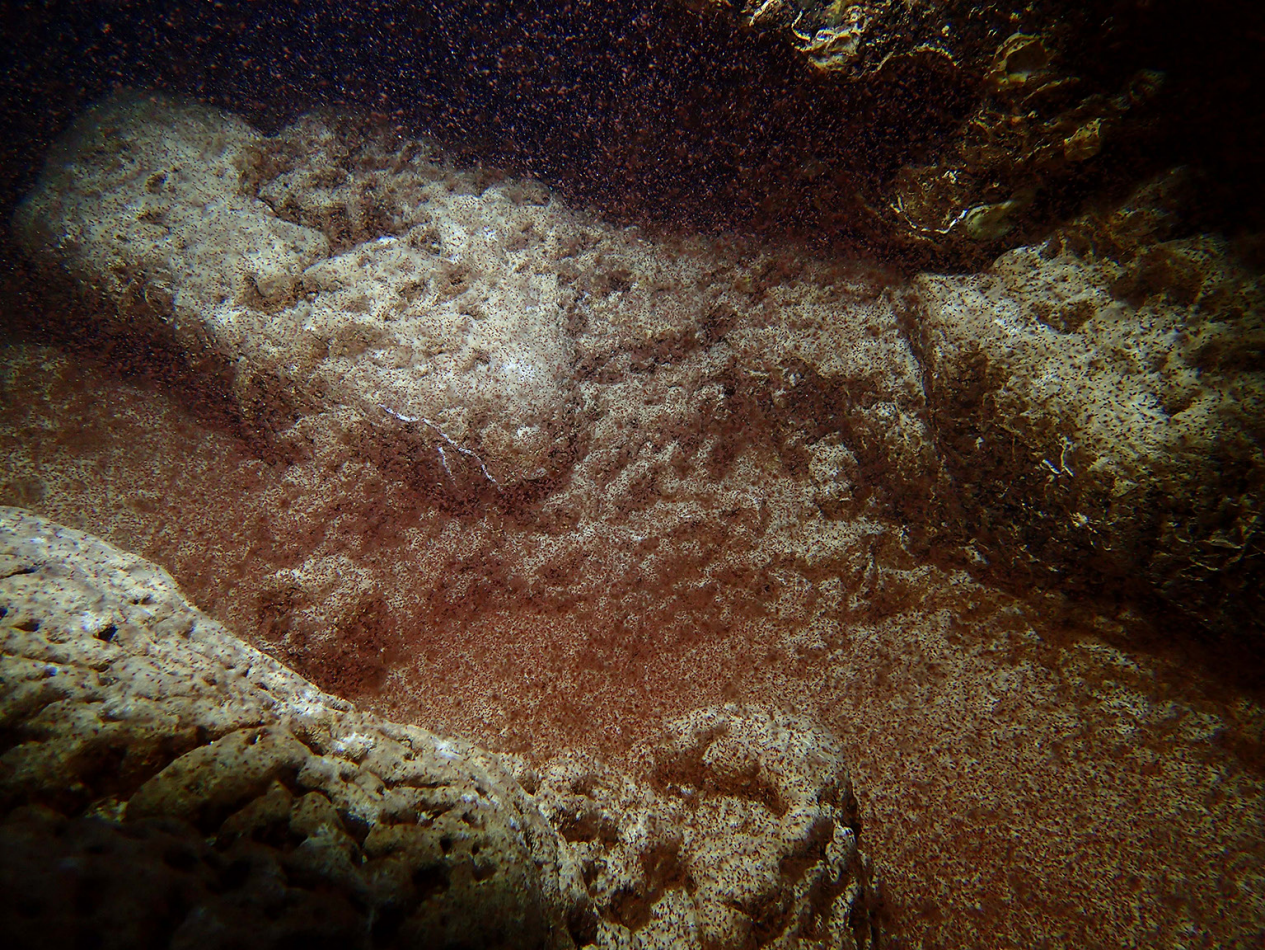
Dense swarm of *Palaumysis antillensis* sp. nov. observed in the innermost dark zone of Cathedral Cave in Guadeloupe. Photo P. Chevaldonné.

(Figs 10I, 28). Type locality is the submarine Full Moon Cave (Station 4) in Bequia. The species was also recorded from the submarine Cathedral Cave (Station 13) in Guadeloupe and the anchialine El Brinco Cave (Station 23) in Cuba, total range 13–22°N, 61–81°W, 13–15 m depth. During the day the mysids formed dense swarms (Fig. 28) in dark recesses inside large caves. The species is classified as a troglophile or a stygophile.

##### Remarks.

The new species differs geographically and, as far as known, also genetically from the above described closest congener *P. bahamensis* by 3- to 5-segmented (vs 2-segmented) mesial antennular flagellum with apical segment measuring 1/5–1/6 (vs 4/5) flagellum length, antennal scale with its longest seta longer (vs shorter) than 1/2 scale length, and apex of male pleopod 4 with one large and one short seta, the former bearing bilateral series of small cils from submedian to subapical portions vs apex with large smooth seta only.

The new species differs from the W-Pacific *P. simonae* (described above) by large seta accompanied by one or two short setae at the apex of the antennal scale (vs total of only one or two minute setae), apex of pleopod 1 with only moderately-sized setae in both sexes, no large seta (vs large plumose seta present in male) and length of antennal scale 3–4 × (vs 4–5 ×) maximum width.

The NW-Pacific *P. philippinensis* and *P. pilifera* share a (sub)rectangular posteroventral edge of the carapace and tip of antennal scale with only one seta vs well-rounded edge of carapace and tip of antennal scale with two or three setae in the new species.

### Subfamily Heteromysinae Norman, 1892

#### Tribe Mysidetini

Taxon classificationAnimaliaMysidaMysidae

Holt & Tattersall, 1906

4E61B99C-A528-53D1-A968-C72B698748B8

Mysidetinae Holt & Tattersall, 1906: 39 (new subfamily, definition, type genus defined); Hansen 1910: 4 (unaccepted); W.M. Tattersall 1923: 277 (withdrawal in favor of Leptomysini).
Mysidetini : Wittmann et al. 2014: 341, 346 (re-establishment at tribe level, diagnosis, in key); San Vicente 2017: 166 (distribution); Kou et al. 2020: Table 1 (molecular analysis); Wittmann and Chevaldonné 2021: 166, Table 1 (morphology, sensory structures); Daneliya 2023: 415 (short diagnosis).

##### Type genus.

*Mysidetes* Holt & Tattersall, 1906.

##### Diagnosis.

Slightly modified after Wittmann et al. (2014): Heteromysinae with appendix masculina well-developed to reduced; thoracic endopods 3–8 normal, pediform; endopod 3 not prehensile, not conspicuously swollen, carpopropodus with two or more (in some species many) segments; penes mostly long, tubular, stiff or changeable in size and form; pleopods reduced to simple, unsegmented setose plates in both sexes, no modified setae or spines; telson entire or with apical cleft, lateral margins with spines.

##### Seven genera included.

Bold italics highlight two genera with species recorded from Caribbean caves: ***Bermudamysis*** Băcescu & Iliffe, 1986, *Burrimysis* Jaume & Garcia, 1993, *Deltamysis* Bowman & Orsi, 1992, *Mysidetes* Holt & Tattersall, 1906, *Mysifaun* Wittmann, 1996, ***Platyops*** Băcescu & Iliffe, 1986, and *Pseudomysidetes* W.M. Tattersall, 1936.

#### Genus Platyops

Taxon classificationAnimaliaMysidaMysidae

Băcescu & Iliffe, 1986

C0912470-918E-59B1-AAC8-47036B8F2B28

Figs 29-32

Platyops Băcescu & Iliffe, 1986a: 100 (diagnosis); Pesce et al. 1994: 117 (in list of subterranean fauna); Fukuoka 2006: fig. 1 (taxonomy, geographic distribution); San Vicente and Monniot 2014: 334, 341 (taxonomy, in key); Wittmann et al. 2014: 341 (taxonomy, transfer to Mysidetini); Daneliya 2021: 45 (revision, transfer to Heteromysini).

##### Type species.

*Platyops sterreri* Băcescu & Iliffe, 1986, by monotypy. Syntypes here examined and lectotype defined.

##### Revised diagnosis.

Mysidetini with large, subquadrangular to subcircular, flattened eyestalks, no eye papilla; cornea or its rudiments implanted laterally; antennular trunk with normal setae (including, if any, whip setae), no flagellate spines, no backward-directed modified setae; antennal scale, pleopods and uropods setose, no spines, no teeth; thoracic endopod 3 non-prehensile, with 2- to 3-segmented carpopropodus; endopods 4–8 normal, praeischium without distal projection, ischium and merus without flagellate spines; marsupial chamber formed by oostegites from sympods 7–8; penes tube-like, <1/5 BL; structure of pleopods non-dimorphic, reduced to unsegmented setose plates with inconspicuous rudiments of pseudobranchial lobes; uropods normal, both rami undivided; telson trapezoid to triangular with bare proximal half, only distal ≤1/2 of lateral margins with spines, its transverse terminal margin not incised or with an only minute incision, no laminae, no setae.

**Figure 29.**
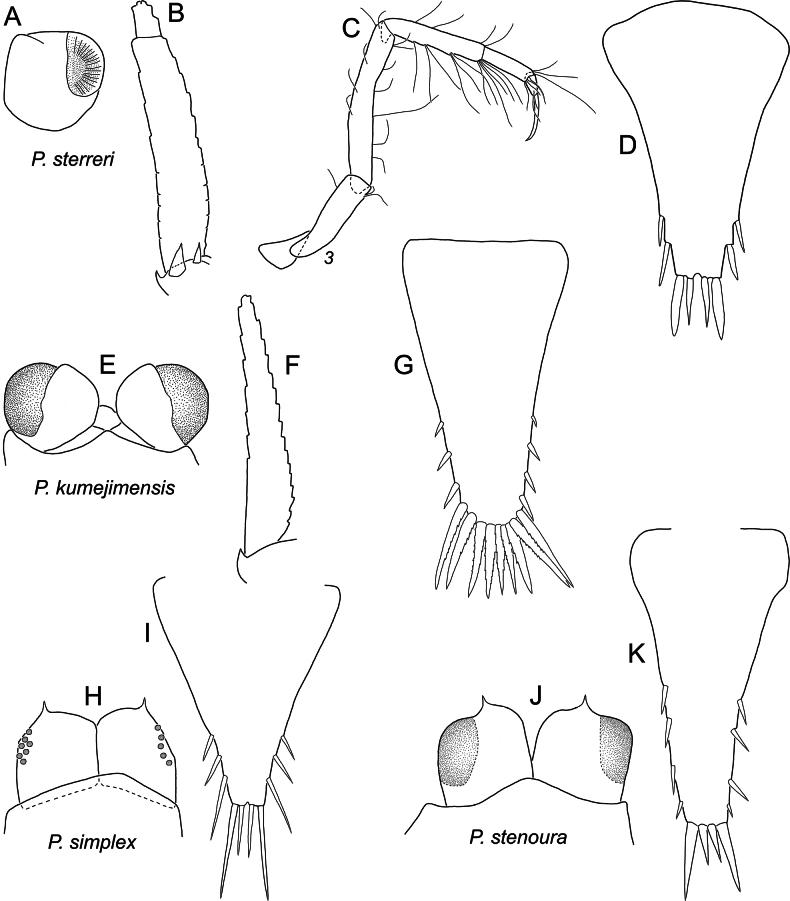
Differentiation between all acknowledged species of *Platyops* Băcescu & Iliffe. **A–D**. Right eye, dorsal (**A**), antennal scale, setae omitted (**B**), thoracic endopod 3 (**C**) and telson (**D**) in *P. sterreri* Băcescu & Iliffe. **E–G**. Eyes with anterior margin of carapace (**E**), antennal scale, setae omitted (**F**) and telson (**G**) in *P. kumejimensis* (Shimomura & Fujita); **H, I**. Eyes with anterior margin of carapace (**H**) and telson (**I**) in *P. simplex* (Hanamura & Kase); **J, K**. Eyes with anterior margin of carapace (**J**) and telson (**K**) in *P. stenoura* (Hanamura & Kase). Modified after Shimomura and Fujita 2020 (**E–G**), Hanamura and Kase 2001b (**H, I**) and Hanamura and Kase 2004 (**J, K**); original drawings (**A–D**).

##### Species inventory.

Four species are acknowledged and given in below key to species; bold italics highlight two species recorded from Caribbean caves: ***Platyops sterreri*** Băcescu & Iliffe, 1986, *P. simplex* (Hanamura & Kase, 2001), ***P. stenoura*** (Hanamura & Kase, 2004) and *P. kumejimensis* (Shimomura & Fujita, 2020), comb. nov. The latter species recombined from *Heteromysoides kumejimensis* Shimomura & Fujita, 2020, due to conforming with the revised diagnosis of the genus *Platyops* above, unlike *Heteromysis* and *Deltamysis*. This recombination is necessary since Daneliya (2021) synonymized the genus *Heteromysoides* with *Heteromysis* and divided it between *Heteromysis*, *Platyops*, and *Deltamysis* Bowman & Orsi, 1992.

##### Diagnosis and affiliation of the genus *Platyops*.

**Figure 30.**
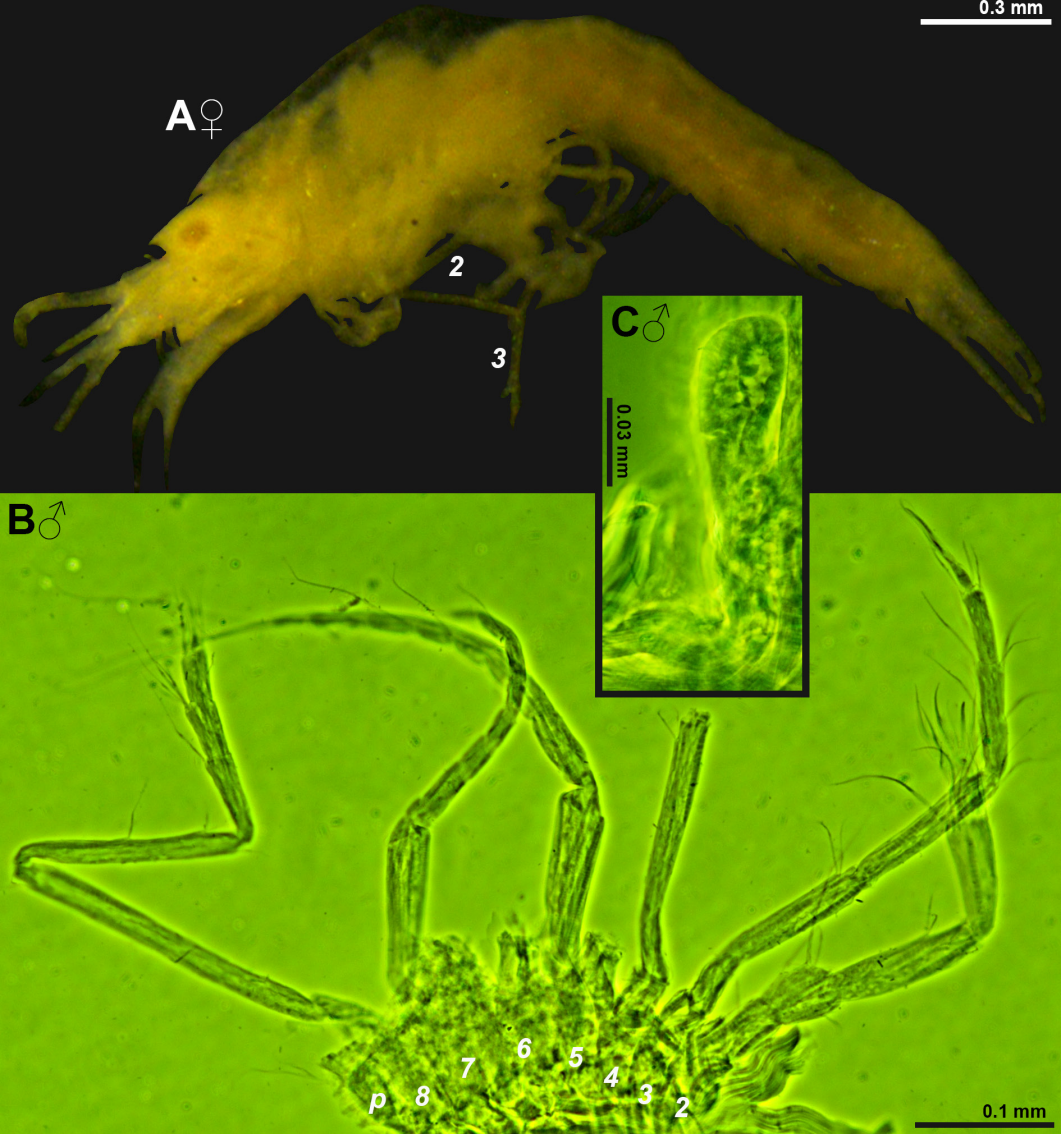
Female habitus, male thoracopods and penis in *Platyops sterreri* Băcescu & Iliffe. **A**. Lectotype ♀ from Castle Grotto in Bermuda; **B, C**. Non-type ♂ from Cathedral Cave in Guadeloupe. **A**. Habitus, lateral, numerals indicate thoracic endopods 2 and 3, object artificially separated from background; **B**. Thoracic sympods 2–8 with endopods 2–4, 8, exopods 5, 7, and penis (*p*); **C**. Detail of (B) showing penis.

**Figure 31.**
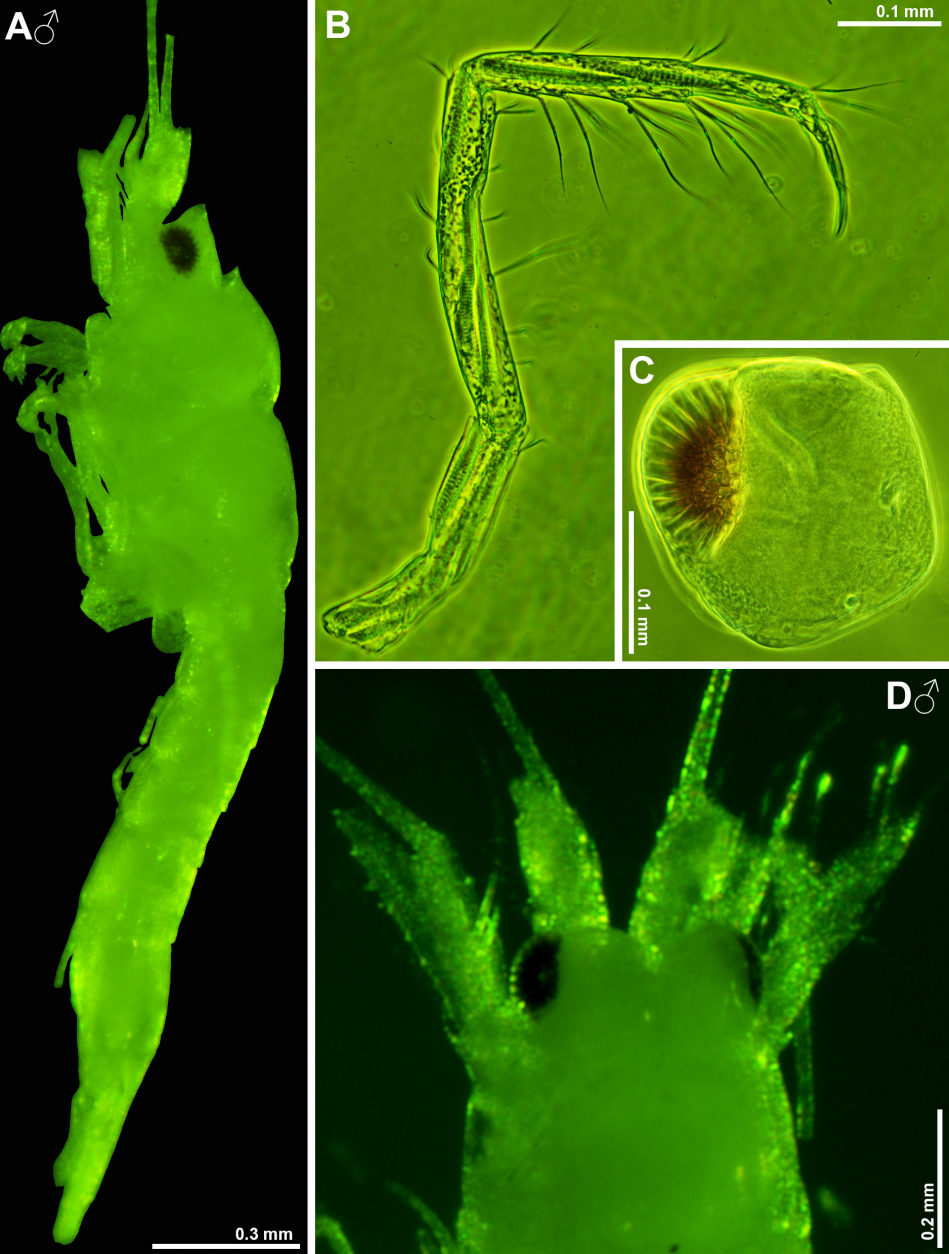
Male habitus, eye and thoracopod 3 in *Platyops sterreri* Băcescu & Iliffe ♂ from Cathedral Cave in Guadeloupe. **A**. Habitus, lateral, most thoracopods broken, object artificially separated from background; **B**. Thoracic endopod 3; **C**. Left eye, dorsal; **D**. Cephalic area, dorsal.

Daneliya (2021) stated upon revision of the genus *Platyops* “The most characteristic feature of this genus is the eye structure, particularly the presence of a strong anterior spine, directed forward”. As shown below, there is no such spine in the types of the type species *P. sterreri*. Actually, a spine is feigned only in lateral view (Figs 30A, 31A), but clearly no spine-like process is visible in dorsal view (Fig. 31C). The laterally wide, transverse, anterior margin of the distally wedge-shaped eyestalk was correctly interpreted already upon original description by Băcescu and Iliffe (1986a) as “... strongly flattened chisel-like …”. The here-confirmed absence of a spine clearly contradicts the revision by Daneliya (2021). For the revised diagnosis of the genus, see above.

**Figure 32.**
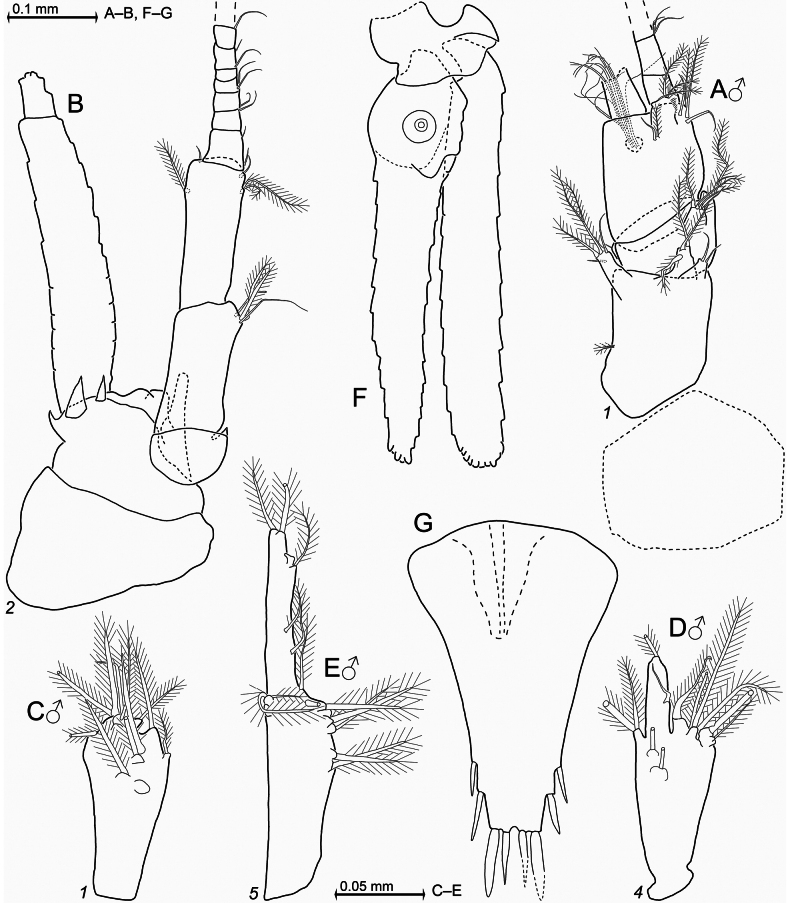
Antennae, pleopods and tail fan in *Platyops sterreri* Băcescu & Iliffe ♂ from Cathedral Cave in Guadeloupe. **A**. Left antennula, dorsal, and relative position of clypeus (dashed line) on ventral face; **B**. Right antenna, ventral, setae omitted from antennal scale; **C–E**. Pleopods 1, 4, 5, ventral view on rostral face; **F**. Right uropods, dorsal, setae omitted; **G**. Telson, dorsal.

By including *Heteromysoides dennisi* Bowman, 1985, whose eyestalks bear a spine (Fig. 43A) and which has a prehensile endopod 3 (Fig. 43B), Daneliya (2021) amplified the diagnosis of *Platyops* in favor of transferring this genus from the tribe Mysidetini to the Heteromysini. The present examination of the type specimens of the type species *P. sterreri* at the MINGA confirms a normal non-prehensile structure of this endopod and the absence of an eyestalk spine (Figs 30, 31). Consequently, the genus *Platyops* is here retransferred to the Mysidetini, where it had been previously placed by Wittmann et al. (2014), mainly based on the non-prehensile structure of thoracic endopod 3 (Figs 30A, 30B, 31B).

##### Affiliation of *Heteromysoides kumejimensis* Shimomura & Fujita, 2020.

Daneliya (2021) synonymized the genus *Heteromysoides* Băcescu, 1968, with the genus *Heteromysis* S.I. Smith, 1873, based on morphology of the type species *Heteromysoides spongicola* Băcescu, 1968. He did not mention the publication of Shimomura and Fujita (2020), so *Heteromysoides kumejimensis* may have escaped the revision by Daneliya (2021) merely by temporal overlap. This species fits the above definition of the genus *Platyops* by the antennular trunk bearing only normal setae, non-prehensile thoracic endopod 3 with 3-segmented carpopropodus, non-dimorphic pleopods reduced to setose plates and by a trapezoid, terminally slightly convex telson with bare basal half (Fig. 29G). Structure of thoracic endopod 3 and telson exclude affiliation with *Heteromysis*. Rather than perpetuation of a ‘ghost’ combination, we propose recombination as *Platyops kumejimensis* (Shimomura & Fujita, 2020).

#### Platyops sterreri

Taxon classificationAnimaliaMysidaMysidae

Băcescu & Iliffe, 1986

4FC238B2-57DD-53B9-8DFA-9679C0867D86

Figs 29A–D, 30–32

Platyops sterreri Băcescu & Iliffe, 1986a: 100–102, fig. 2A–L (original description); Pesce et al. 1994: 117 (in list of stygophile species); Wittmann and Ariani 2019: suppl. (statolith composition); Daneliya 2021: 45, 46 (taxonomy); Iliffe and Calderón-Gutiérrez 2021: Table 1 (stygobiont species, Bermuda); Wittmann 2023: table SS1 (non-visual eyestalk organs).

##### Type material examined.

Bermuda • Here designated ***lectotype***, ♀ ad. (BL = 1.9 mm, TL = 2.4 mm, MINGA MYS 448, Fig. 30A) from Castle Grotto (Station 28) • ***paralectotype***, 1 damaged ♀ ad. (BL = 2.1 mm, MINGA MYS 449), sampling data as for lectotype.

##### Other material.

Bermuda • 1 ♀ ad. (also with BL 2.1 mm, MINGA MYS 451) from Walsingham Cave (Station 29) — Lesser Antilles **Guadeloupe** • 1 ♂ ad. (BL = 2.0 mm) on slides (NHMW-ZOO-CR-31428) from Cathedral Cave (Station 14).

##### Identification of the types.

Previously, there was no entry in the inventory of the MINGA, and the species is therefore not listed by Petrescu and Wittmann (2009). Vials with handwritten labels by Mihai Băcescu were recently identified by Iorgu Petrescu (pers. comm. 2025) as containing the syntypes of *P. sterreri*.

An additional vial with one female specimen (MINGA MYS 451) is labeled “Sus 1 ♀ j ..... st. 6 Platyops mirandus”, whereby “st. 6" refers to Walsingham Cave in Băcescu and Iliffe (1986a). *Platyops mirandus* is an unpublished nomen nudum, and this material therefore cannot considered part of the type series. Nonetheless, this specimen is here determined as *P. sterreri*.

##### Revised diagnosis.

*Platyops* with eyestalks forming thick plates with distal 1/5–1/6 obliquely truncate in the transversal plane, resulting in dorsal face extending more anteriorly than the ventral face, no eye papilla, no spine, no teeth; antennal scale with small apical segment; thoracic sympod 3 subequal to sympod 2; endopod 3 less stout than endopod 2, endopod 3 not prehensile, with 2- to 3-segmented carpopropodus; penes tube-like, only 1/20 BL; endopod of uropods approx. as long as exopod; telson trapezoid, length without spines 1.3–1.5× maximum width near basis, terminal width 1/4 maximum width; telson with proximal 2/3 bare, distal 1/3 of each lateral margin with two or three spines, truncate terminal margin with pair of large distolateral spines flanking a paramedian pair of shorter spines, minute median notch present.

##### Supplementary notes on females.

Lectotype from Castle Grotto (Station 28): rostrum measures 3% BL, carapace without rostrum 30%, cephalothorax 38%, pleon without telson 49%, and telson 10%. Eyestalks form thick plates with distal 1/5 obliquely truncate in the transversal plane. Eye 2.0× longer than wide, structure as described below for males. Cornea fully functional, lens-shaped in dorsal view (as in Fig. 31C, D), diameter 0.10 mm, length (thickness) 0.06 mm; this is 1/2 length of eyestalk (cornea not included). Cornea dorsally flattened and even flatter ventrally as seen in lateral view, length 1.6× maximum width. Pigment partly retracted from cornea. Thoracic endopod 3 non-prehensile, thicker than endopods 5, 7. Carpopropodus 3, 5, 7 with 3, 3, 4 segments, respectively, slenderness increasing caudally. Pleopod 5 measures 1/16 BL, reaching 1/2 length of pleomere 6.

Non-type from Walsingham Cave (Station 29): carpopropodus of thoracic endopods 3–8 with 3, 6, 4, 4, 4 and 4 segments, respectively. Endopod 4 more slender than the remaining endopods.

##### First description of the male.

Based on non-type from Guadeloupe (Station 14). Cephalothorax comprises 36% BL, pleon (without telson) 44%, telson 17%, carapace (without rostrum) 31%, and rostrum 3%. Clypeus pentagonal, wider than long (lower dashed line in Fig. 32A). Thoracic sternites without mid-ventral processes, no spines, no setae.

***Carapace***. Rostrum broadly rounded, as long as 0.4× the terminal segment of antennular trunk in dorsal view. The cervical sulcus forms a deep transverse incision (Fig. 31A). The post-cervical part of carapace as long as 1.2× cervical part measured along midline without rostrum. Caudolateral margin of carapace broadly rounded; posterior margin slightly concave, leaving the ultimate thoracomere mid-dorsally exposed.

***Eyes*** (Fig. 31A, C, D). Eye 11% BL, subquadrangular with transverse, weakly convex, anterior margin in dorsal view (Fig. 31C, D). Eye forming a 1.8× longer than wide, proximally rectangular trapezoid with obliquely truncate distal margin; this renders dorsal margin longer than ventral margin in lateral view (Fig. 31A). Cornea fully functional, lens-shaped in dorsal view (Fig. 31C, D), diameter quite variable, 0.6–0.9× eyestalk length (cornea not included), cornea length (thickness) ≈1/3 length of eyestalk. Cornea oval in lateral view, length 1.9× maximum width (ventral margin covered by left antennular trunk in Fig. 31A).

***Antennula*** (Fig. 32A). Antennular trunk 3-segmented, well setose, no spines, no teeth. Trunk extends 0.5–0.6× its length beyond eyes in normal orientation. Measured along dorsal midline, the basal segment is 42% trunk length, median segment 20%, and terminal segment 38%. Lateral margin of basal segment at half-length with two minute barbed setae; subterminally with a rectangular, distally setose lobe anteriorly approaching but not extending beyond rostral margin of the median segment. On dorsal face near mesial margin distally with two setose apophyses. Dorsal face of median segment disto-mesially with one setose apophysis. Terminal segment with the usual mid-dorsal lobe bearing a few barbed setae, no spines, no teeth. Lateral margin bare, disto-mesial edge with diverse barbed and smooth setae. Appendix masculina well setose despite its small size, only 8% length of terminal segment. Flagella damaged.

***Antenna*** (Fig. 32B). Sympod 2-segmented, no setae. Distolateral edge extended into a tooth-like projection. Sympod with two triangular acutely pointed teeth subterminally below antennal scale; one digitiform lobe above basis of peduncle (dashed line in Fig. 32B). Peduncle 3-segmented, basal segment contributes 17% to total length of peduncle, median segment 39%, and terminal segment 44%. Antennal scale with small terminal segment, total length of scale 5× maximum width. Scale extends 1/4 its length beyond antennal peduncle and 0.2–0.3× its length beyond antennular trunk.

***Mouthparts***. Labrum rostrally rounded. Proximal segment of left mandibular palp 14% palp length, no setae. Length of median segment 2.7× its maximum width and 64% palp length. Central half of mesial margin of median segment with three basally barbed whip setae, central half of lateral margin with four setae of that type, lateral margin with an additional subterminal seta, transverse terminal margin with two setae on mesial corner. Terminal segment well setose, 22% palp length. Pars molaris of left mandible with well-developed grinding surface. Pars incisiva and digitus mobilis each with ≈3 teeth, pars centralis with four spines bearing stiff bristles. Right mandible, labium and both maxillulae damaged. Distal 2/3 of endopod of maxilla all around well setose; its distal segment 1.7–1.8× as long as maximum width; proximal segment mesially with four basally barbed whip setae with thick handle. Lateral margin of exopod with 10 or 11 plumose whip setae, terminal margin with two such setae.

***Foregut***. Gross structure as in Fig. 23. Lateralia anteriorly each with apically pronged spines, with bare shaft; more caudally with group of four or five unilaterally weakly serrated spines as in Fig. 23D. Dorsolateral infoldings each with pair of long spines unilaterally bearing series of small teeth increasing in size distally along distal 4/5 of shaft.

***Thoracopods*** (Figs 30B, 31B). Width decreases from thoracic sympod 1 to 5 and then increases from 5 to 8. Flagellum of exopods 3–7 with 8, 9, 9, 9, 8 segments, respectively, remaining exopods damaged or broken. Basal plates expanded, 2–3× as long as wide, distolateral corner well rounded. Total length of endopods increases in series of endopods 1–3, 6, 8, remaining endopods broken. Thoracopod 1 with leaf-like, smooth epipod. Tip of coxa of endopod 1 with one short whip seta with barbed handle. Basis with large, prominent endite that is densely setose on mesial and terminal margins. Ischium and merus with short endites. Nail 1 thick, slightly bent, approx. as long as dactylus. Basis of endopod 2 with weakly developed mesial endite bearing two sparsely barbed setae. Combined praeischium plus ischium are 0.8–0.9× length of merus, carpopropodus plus dactylus 1.1–1.2× merus. Dactylus large, densely setose, mainly by whip setae with handle basally, bilaterally bearing stiff barbs. Nail smooth, approx. as long as dactylus; nail 2 thinner than nail 1. Endopod 3 non-prehensile, its carpopropodus, if 2-segmented (Figs 30B, 31B), with proximal segment longer than combined distal segment and dactylus; terminal margin of distal segment with only one paradactylar whip seta (plus other setae) bearing barbed handle and smooth flagellum; nail 3 smooth, weakly bent, approx. as long as distal segment of carpopropodus. Endopod 6 slender; carpopropodus 3-segmented with proximal segment shorter than combined median and distal segments; terminal margin of distal segment with two smooth, paradactylar whip setae (plus other setae); nail as in endopod 3. Penes as in Fig. 30C.

***Pleon and pleopods*** (Figs 31A, 32C–E). Pleomeres 1–5 are 0.8, 0.6, 0.6, 0.7 and 0.8 times the length of pleomere 6, respectively. Length of pleopods 1–5 increasing distally. Pleopod 5 (Fig. 32E) measures 1/12 BL and 0.9× length of pleomere 5, thus reaching to 2/3 caudal extension of pleomere 6. Pleopod 5 not fully visualized in Fig. 31A. Pleopods 1–4 (Fig. 32C, D) with barbed (plumose) setae only on distal half; pleopod 5 with setae on distal 2/3.

***Tail fan*** (Fig. 32F, G). Scutellum paracaudale short, sinusoid, laterally covering part of the telson basis. Both rami of uropods setose all around. Exopod approx. as long as endopod, each caudally reaching 0.4× their length beyond telson. Exopod 7× longer than wide. Diameter of the spherical fluorite statoliths 34–38 μm. Telson 17% BL, equaling 1.1× length of pleomere 6. Telson length 1.5× maximum width; distolateral spines 22% telson length.

##### DNA data.

Only one 622 bp mitochondrial COI sequence was obtained from a single individual from Cathedral Cave (GenBank PV990329).

##### Distribution and habitat

(Fig. 10L). Type locality not stated originally. Here fixed as Bermuda, submarine cave Castle Grotto (Station 28). The species was first described by Băcescu and Iliffe (1986a) from two submarine caves 4 km apart in Bermuda: Castle Grotto and Green Bay Cave. The present record from Cathedral Cave (Station 14) in Guadeloupe is the first from outside Bermuda, namely 1770 km away. Total distribution range 16–32°N, 62–65°W, 0.5–18 m depth. From submarine caves of Bermuda (Băcescu and Iliffe 1986a) and Lesser Antilles; at Bermuda also found over the open sea floor during the night (Wittmann and Ariani 2019; Wittmann 2023). At Guadeloupe, the species was not directly observed *in situ*, but captured together with large numbers of *Palaumysis antillensis* sp. nov. with which it shares the dark zone habitat of Cathedral Cave (Station 14). *Platyops sterreri* is considered a stygophile (Pesce et al. 1994) or a troglophile (Wittmann 2023).

#### Platyops stenoura

Taxon classificationAnimaliaMysidaMysidae

(Hanamura & Kase, 2004)

FD303C23-C583-5C8F-B0AF-582C16CFD1F6

Fig. 29J, K

Heteromysis stenoura Hanamura & Kase, 2004: 2148–2151, figs 2, 3 (original description, Grand Cayman Island).
Platyops stenoura : Daneliya 2021: 45, 46 (revised assignment, new combination).

##### Short diagnosis.

*Platyops* with eyestalks forming thick plates with sharp spiniform mid-anterior projection, comparatively small cornea implanted laterally, cornea composed of numerous densely-set ommatidia; antennal scale undivided; thoracic sympod 3 subequal to sympod 2; endopod 3 less stout than endopod 2, endopod 3 non-prehensile, with 3-segmented carpopropodus; penes tube-like, ≈1/6 BL; endopod of uropods approx. as long as exopod; telson trapezoid, length without spines 1.8× maximum width near basis, terminal width 1/4 maximum width; telson with bare proximal half, distal half with four or five spines on each lateral margin; transverse terminal margin with pair of large distolateral spines flanking a paramedian pair of spines with 1/2–2/3 length of the distolateral spines.

##### Distribution

(Fig. 10K). Known only from the submarine cave ‘Mouse Trap’ (type locality) ≈400 m off the western coast of Grand Cayman Island, 19°19.25'N, 81°23.38'W, depth 9–11 m (as *Heteromysis stenoura* in Hanamura and Kase 2004).

#### Genus Bermudamysis

Taxon classificationAnimaliaMysidaMysidae

Băcescu & Iliffe, 1986

9C4048EB-BF75-57A8-BA9C-4F77C8C10E7D

Figs 33, 34, 35, 36, 37, 38, 39, 40, 41, 42

Bermudamysis Băcescu & Iliffe, 1986a: 95, 96 (definition, short description); Iliffe 1992a: 623 (in list of anchialine taxa); Pesce et al. 1994: 117 (in list of subterranean taxa); Nouvel et al. 1999: 79 (morphology, taxonomy, in list of genera); San Vicente and Monniot 2014: 333, 341 (taxonomy, in key); Wittmann et al. 2014: 324, 341 (taxonomy, biogeography, transfer to Mysidetini); Daneliya 2021: 6 (taxonomy, claimed transfer to Heteromysini).

##### Type species.

*Bermudamysis speluncola* Băcescu & Iliffe, 1986, by monotypy.

##### Species inventory.

*Bermudamysis speluncola* from Bermuda and *B. caribbaea* sp. nov. from islands of the Lesser Antilles are described below and given in key to species.

##### Revised diagnosis.

Designed for inclusion of the below-described *B. caribbaea* sp. nov. and underlining differences from the genus *Mysidetes* Holt & Tattersall, 1906: Mysidetini with well-developed eyes; antennal scale setose all around, with small apical segment, no spines, no teeth; thoracic endopod 3 normal, non-prehensile, its carpopropodus with 2–4 segments, endopods 4–8 with 3- to 6-segmented carpopropodus; female sympod 6 with vestigial oostegite; marsupial chamber formed by oostegites from sympods 7–8; penes tube-like, <1/4 BL; pleopods non-dimorphic, reduced to unsegmented setose plates with inconspicuous rudiments of pseudobranchial lobes, no spines, no teeth; distal portion of pleopods 1–4 not longer than basal portion marked by the pseudobranchial rudiments; uropods setose all around, no spines; telson terminally incised; cleft with laminae (plus one small spine subterminally on each side in the type species); lateral and terminal margins of telson with spines, no setae; nauplioid larvae with smooth cuticle all around, no cercopods.

##### Remarks.

As already discussed above for *Platyops*, Wittmann et al. (2014) placed *Bermudamysis* within the tribe Mysidetini mainly based on the non-prehensile structure of thoracic endopod 3. Daneliya (2021) transferred *Bermudamysis* to the Heteromysini by valuating a 2-segmented carpopropodus of thoracic endopod 3 rather than the (more complex) prehensile structure in Heteromysini as decisive. However, Băcescu and Iliffe (1986a) had indicated a 2-segmented carpopropodus 3 only in drawing (fig. 1G) but a 3-segmented carpopropodus (expressed as “4-segmented tarsus”) in text (p. 97) upon original description of the type species *B. speluncola*. The different numbers are due to individual variability as confirmed by present findings of 2- to 3-segmented carpopropodites 3 in the type specimens. In addition, the here-described *B. caribbaea* sp. nov. has 3- to 4-segmented carpopropodites 3. This indicates that segmental numbers of carpopropodites are more variable (as expected) in serial pediform endopods compared with stand-alone prehensile endopods. Consequently, *Bermudamysis* is here retransferred to the Mysidetini.

Băcescu and Iliffe (1986a) provided a short diagnosis of the genus *Bermudamysis*, regarding the marsupium by stating “Two marsupial laminae with 2 or 4 eggs, ....” and stating for the type species *B. speluncola* “It is provided with only 1 pair of oostegites and bears 2 or 4 genital produces”. The counts of oostegites are revised by the present findings (Fig. 36B–D) of two pairs of fully functional oostegites (as in most Mysidae) plus one pair of vestigial oostegites in the type specimens.

According to the above-revised diagnosis the genus *Bermudamysis* differs from the genus *Mysidetes* by shorter pleopods 1–4 in both sexes, nauplioid larvae without cercopods, and by the combination of shorter penes, carpopropodus of thoracic endopods 3–8 with fewer segments and endopod of uropods without spines.

#### Bermudamysis speluncola

Taxon classificationAnimaliaMysidaMysidae

Băcescu & Iliffe, 1986

223566FA-0C69-5F5C-9B50-FD2A88ADE1C8

Figs 33, 34, 35, 36

Bermudamysis speluncola Băcescu & Iliffe, 1986a: 96–99, fig. 1A–J (original description); Iliffe 1996: e. T2766A9478950: 1–3 (Red List assessment as critically endangered); Wittmann and Ariani 2019: suppl. (statolith composition, biogeography); Kou et al. 2020: Table 1, figs 7–9 (molecular analysis, phylogenetic analysis); Iliffe and Calderón-Gutiérrez 2021: Table 1, fig. 3C (stygobiont species, Bermuda); Wittmann 2023: table SS1 (troglophile, non-visual eyestalk organs).

##### Note.

Redescription of this non-Caribbean species necessary for differentiation from the below described *B. caribbaea* sp. nov.

##### Type material.

**Figure 33.**
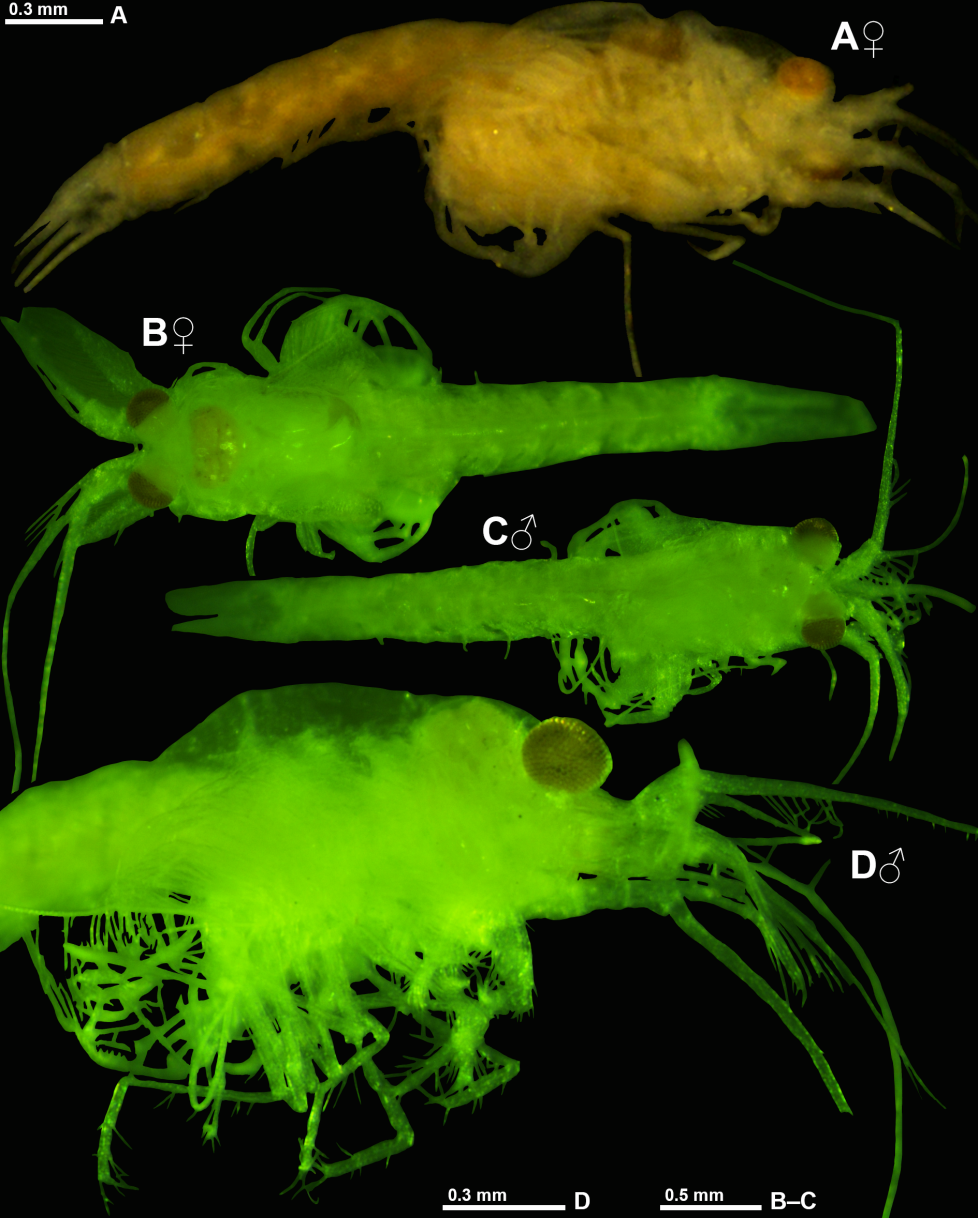
Habitus of *Bermudamysis speluncola* Băcescu & Iliffe, from Bermuda. **A**. Lectotype from Green Bay Cave and **B–D**. Non-types from Grenadier Pool. **A**. ♀ with nauplioid larvae, obliquely lateral; **B**. Another ♀, dorsal; **C**. ♂, dorsal; **D**. Cephalothorax of another ♂, lateral. **A–D**. Objects artificially separated from background.

Bermuda • Here designated ***lectotype***, ♀ (BL = 2.6 mm, TL = 3.3 mm, MINGA MYS 447, Fig. 33A) carrying nauplioid larvae from Green Bay Cave (Station 30) • ***paralectotypes***, 33 ♀♀ ad. (BL = 2.2–2.9 mm, MINGA MYS 446), sampling data as for lectotype • ***paralectotypes***, 2 ♀♀ ad. (BL = 2.2–2.5 mm), 9 ♂♂ ad. (BL = 2.3–2.5 mm), 9 imm., 8 juv. (MINGA MYS 452) from Palm Cave (Station 32) • ***paralectotypes***, 5 ♀♀ ad. (BL = 2.3–2.9 mm), 3 ♂♂ ad. (BL = 2.4–2.7 mm) (MINGA MYS 450) from Cherry Pit Cave (Station 33).

##### Other material.

Bermuda • 3 ♀♀ ad. (BL = 3.2–4.5 mm), 1 ♂ ad. (BL = 3.0 mm), 6 imm., 10 juv. from Green Bay Cave (Station 31) • 1 ♂ ad. (BL = 3.1 mm; ZMH-K-066761) from Deep Blue Cave (Station 34) • 7 ♀♀ ad., 10 ♂♂ ad., 13 imm. (MNHN-IU-2025-2827) from Grenadier Pool (Station 35) • 8 ♀♀ ad., 9 ♂♂ ad., 12 imm., 1 juv. (NHMW-ZOO-CR-31421), sampling data as for preceding • 5 ♀♀ ad., 10 ♂♂ ad., 14 imm., 1 juv. (ZMH-K-066762), sampling data as for preceding • 11 ♀♀ ad., 2 ♀♀ subad., 11 ♂♂ ad., 6 imm. (MNHN-IU-2025-2828), sampling data as for preceding • 5 ♀♀ ad., 1 ♀ subad., 11 ♂♂ ad., 2 ♂♂ subad., 11 imm. (NHMW-ZOO-CR-31422), sampling data as for preceding • 13 ♀♀ ad., 10 ♂♂ ad., 7 imm. (ZMH-K-066763), sampling data as for preceding.

##### Identification of the types.

Just recently discovered and identified as types by Iorgu Petrescu (pers. comm. in 15 Mar. 2024) in vials with hand-written labels by Mihai Băcescu.

##### Type locality and distribution.

Type locality not stated originally, here defined as Bermuda, Green Bay Cave (Station 30). Upon original description, Băcescu and Iliffe (1986a) listed seven cave localities from Bermuda, five of which are represented in the present material (types plus non-types). The mysids were recorded up to now only from polyhaline to euhaline cave waters in Bermuda, depth range 0.5–18 m, where they tend to gather in swarms hovering close to the sediment surface or in the water column (Băcescu and Iliffe 1986a).

##### Revised diagnosis.

*Bermudamysis* with triangular rostrum with rounded apex reaching to at most half-length of basal segment of antennular trunk; antennal scale reaching 0.3–0.5× its length beyond antennular trunk and 0.3–0.4× beyond antennal peduncle; thoracic endopod 3 non-prehensile, endopods 4–8 longer and more slender than endopod 3, endopod 7 the longest, ≈1/2 BL; carpopropodus of endopods 3–8 with 2 or 3, 3, 3, 3, 3 and 3 segments, respectively; penes tubular, <1/10 BL and 0.4–0.6× length of ischium 8; each lateral margin of telson with 4–6 (mostly 4) subequal spines positioned only on distal half; U-shaped terminal cleft penetrating 15–30% telson length, its margins bearing seven to ten long, slender laminae on proximal 2/3; distolateral lobes of telson each with narrow apex ending in one large spine, one additional <1/2 as long spine positioned nearby on each side shortly inside cleft; telson with total of 12–16 spines.

##### Short description of the lectotype

(Fig. 33A). Only external features checked: cephalothorax 29% BL, rostrum 6%, carapace without rostrum 25%, pleon without telson 53%, and telson 12%. Antennal scale length 4.7× maximum width. Scale extends 1/5 its length beyond antennular trunk. Eye length 7% BL. Eyes dorsoventrally compressed by a factor of 1.7. Eye pigment slightly retracted from cornea. Thoracic endopod 3 non-prehensile, its carpopropodus with two segments, carpopropodi 4–6 with three segments. Marsupial chamber formed by large oostegites from thoracopods 7–8, inside with total of four nauplioid larvae. Paramedian pairs of muscle structures, visible as near-pentagonal images (arrows in Fig. 36E), are located in pleomeres 1–3 between neuronal cord and intestine. Each distolateral lobe of telson with large terminal spine mesially inside cleft accompanied by a shorter subterminal spine; each lateral margin with four spines on distal half. Cleft penetrates 1/4 telson length.

##### Non-type supplements to the original description.

**Figure 34.**
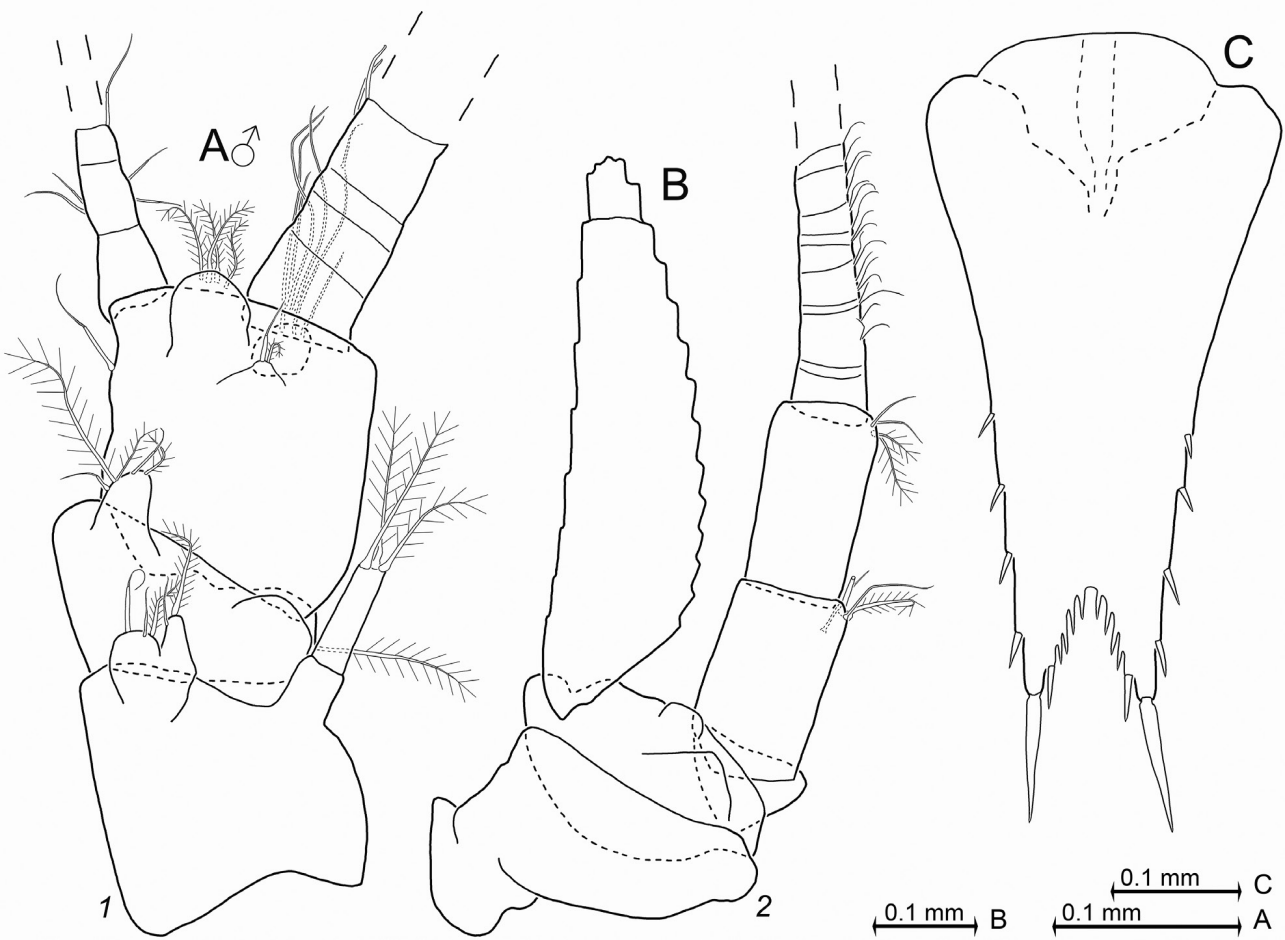
Antennae and telson in *Bermudamysis speluncola* Băcescu & Iliffe material from Grenadier Pool in Bermuda. **A**. Right male antennula, dorsal; **B**. Left antenna with end sac of antennal gland, dorsal, setae omitted from antennal scale; **C**. Telson, dorsal.

**Figure 35.**
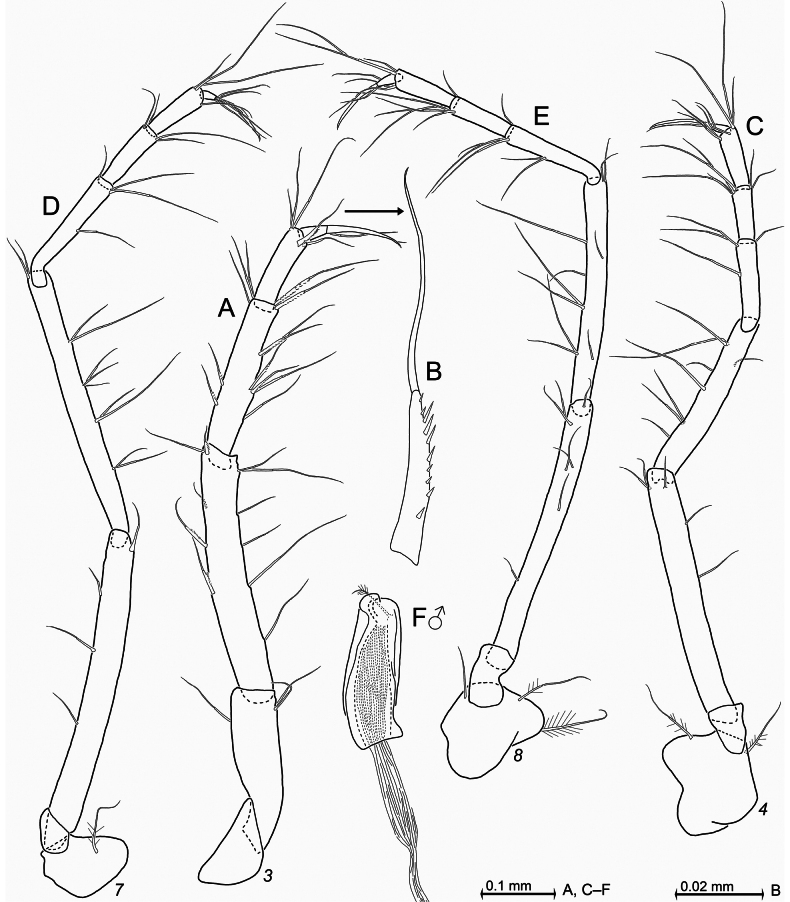
Thoracic endopods 3, 4, 7, 8 and penis in *Bermudamysis speluncola* Băcescu & Iliffe adults from Grenadier Pool in Bermuda. **A**. Endopod 3 from praeischium to dactylus in ♀; **B**. Detail of (**A**) showing proximally spiny paradactylar seta; **C–E**. Endopods 4, 7, 8 in ♂; **F**. Penis.

**Figure 36.**
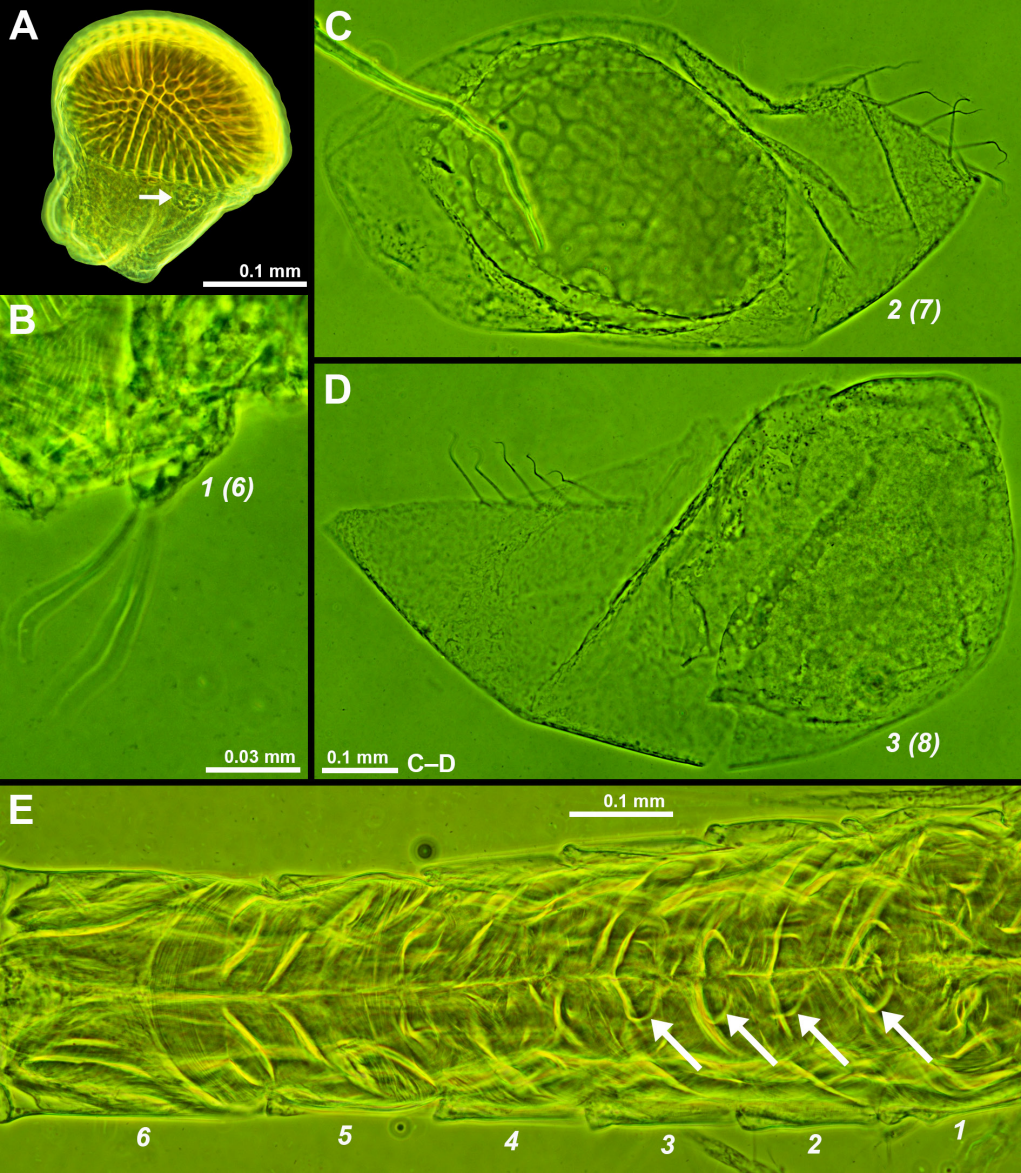
Eye, marsupium and pleon in *Bermudamysis speluncola* Băcescu & Iliffe adults from Grenadier Pool in Bermuda. **A**. Eye of ♂, dorsal, arrow points to OB, object artificially separated from background; **B**. Vestigial oostegite 1 from thoracic sympod 6, both terminal setae marginally at focus; **C**. Oostegite 2 from sympod 7 with egg attached, outer = lateral aspect; **D**. Oostegite 3 from sympod 8, inner = mesial aspect; **E**. Pleon of ♀, dorsal view with focus on muscle tissue inside pleomeres, arrows point to paramedian pairs of muscle structures visible as near-pentagonal images inside pleomeres 1–3. **A–E**. Materials expanded on slide.

Dissected parts included: eye length 0.24–0.29 mm (*n* = 10 adults), this is 6–11% BL. OB completely internal, close to cornea, diameter 28–71 µm (*n* = 6). Setose mesial lobe dorsally at median segment of antennular trunk with length not exceeding width at basis (Fig. 34A) in both sexes. Antennal sympod with two lobes emerging between scale and peduncle, both lobes shorter than basally wide (Fig. 34B). Antennal scale length 3.8–5.1× maximum width. Thoracic epipod 1 leaf-like, with subbasal seta. Thoracic endopod 3 with 2- to 3-segmented carpopropodus, endopod length 1/3 BL, distinctly less slender (Fig. 35A) than endopods 4–8 (Fig. 35C–E). Endopod 3 with smooth paradactylar whip setae except one whip seta with spiny handle and smooth flagellum (Fig. 35B). Merus and carpopropodus of endopod 3 with additional basally spiny whip setae plus several smooth whip setae. Endopods 4–8 with only smooth setae all along (Fig. 35C–E), not considering setae of the sympod. Basal plates of thoracic exopods 1–8 are ≈3× longer than wide, distolateral corner rounded; flagellum with 7, 8, 8, 8, 8, 8, 8 and 7 segments, respectively. Penes terminally trilobate with barbed seta (Fig. 35F). Paramedian pairs of muscle structures, visible as near-pentagonal images (arrows in Fig. 36E), are located in pleomeres 1–3 between neuronal cord and intestine.

***Marsupium*** (Fig. 36B–D). As in most Mysidae, the marsupium is formed by two pairs of fully functional oostegites emerging from thoracic sympods 7, 8. Length of chamber ≈1/3 BL, maximum height and width each 1/6–1/4 BL, the values varying with brood volume by adjustment of the non-locked, motile oostegites. The main large oostegite 3 (Fig. 36D) from sympod 8 forms a deep, rostrally converging bowl, rostrally enclosing part of oostegite 2 and caudally containing, if any, one egg or larva; rostral half of mesial margin with series of sparsely barbed setae. Oostegite 2 (Fig. 36C) from sympod 7 forms a less deep bowl with, if any, again only one egg or larva; rostral margin obliquely truncate, bearing a short series of barbed setae. Outer face of marsupium without setae, no spines. The left and right pairs of oostegites together forming a main chamber with four subchambers (bowls), each providing space for only one offspring. In addition to the main oostegites, a vestigial oostegite 1 is present on sympod 6. This vestige represents a small flat lobe, distally obliquely truncate with two setae on tip. Both setae with minute cils along distal half (only marginally in focus in Fig. 36B).

#### Bermudamysis caribbaea sp. nov.

Taxon classificationAnimaliaMysidaMysidae

7E0C1A66-4EF4-521E-895D-AA0284FBA20A

https://zoobank.org/669B8C63-DAF1-4C14-A1E2-2FE0E33594FC

**Figure 37.**
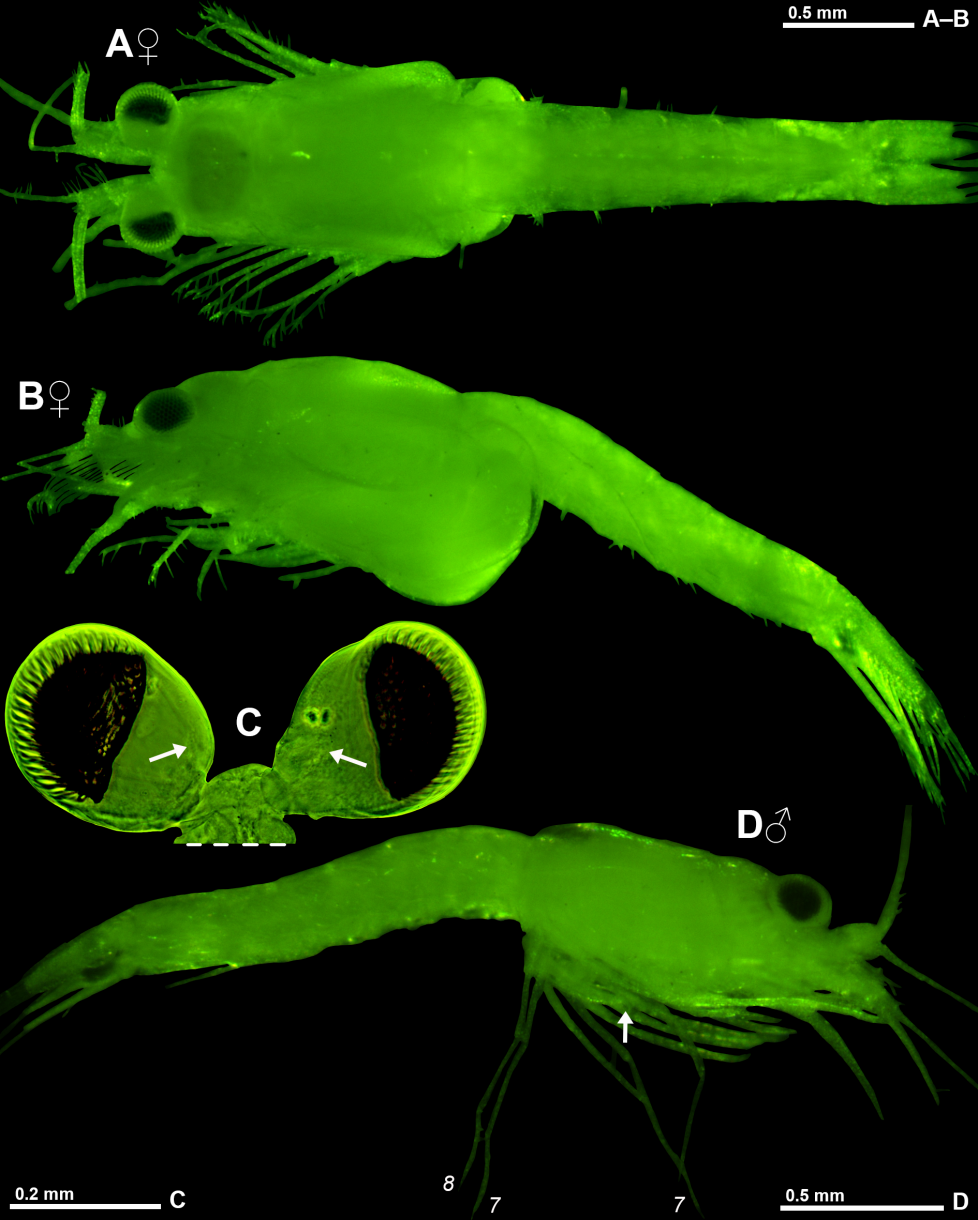
Habitus and eyes in *Bermudamysis caribbaea* sp. nov. adult types from Barracuda Cave in Guadeloupe. **A**. Paratype ♀, dorsal aspect; **B**. Allotype ♀ with nauplioid larvae in the brood pouch, lateral; **C**. Eyes of paratype ♂, dorsal, arrows point to OB; **D**. Habitus of holotype ♂, lateral, arrow points to tip of right penis, numerals indicate thoracic endopods 7 and 8. Objects artificially separated from background.

**Figure 38.**
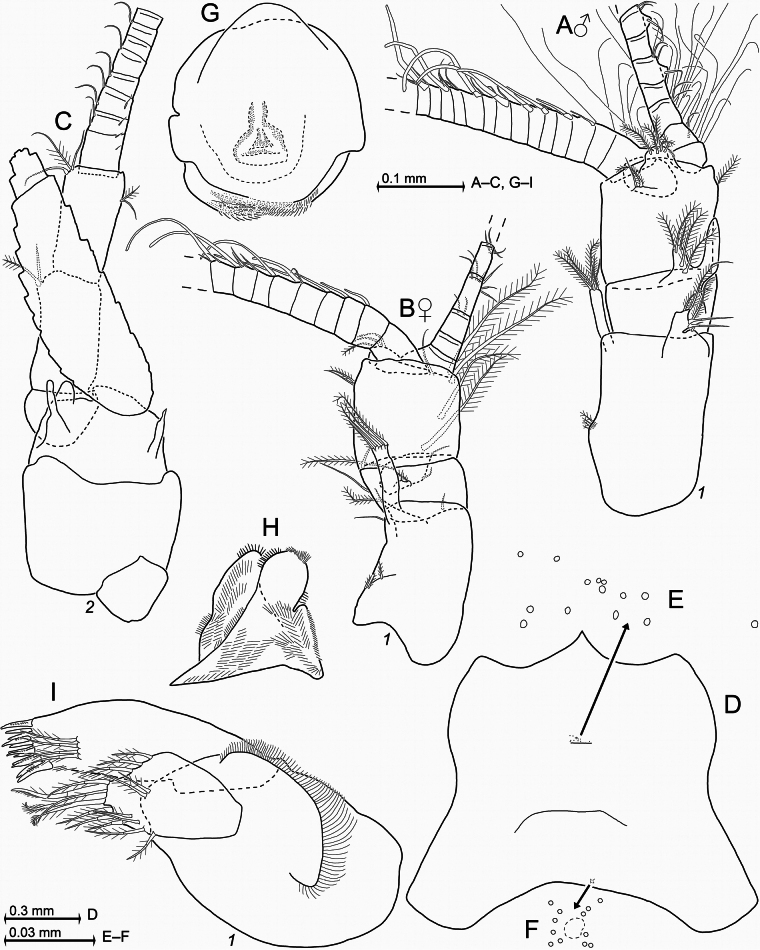
Carapace and part of cephalic appendages in *Bermudamysis caribbaea* sp. nov., adult paratypes from Barracuda Cave in Guadeloupe. **A**. Left male antennula, dorsal; **B**. Right female antennula, ventral; **C**. Right antenna with antennal gland, ♂, dorsal, setae omitted from antennal scale; **D**. Carapace expanded on slide, ♀, dorsal; **E, F**. Details of (**D**) showing cervical (**E**) and caudal (**F**) pore groups; **G**. Labrum, aboral; **H**. Labium, obliquely lateral; **I**. Maxillula, caudal.

**Figure 39.**
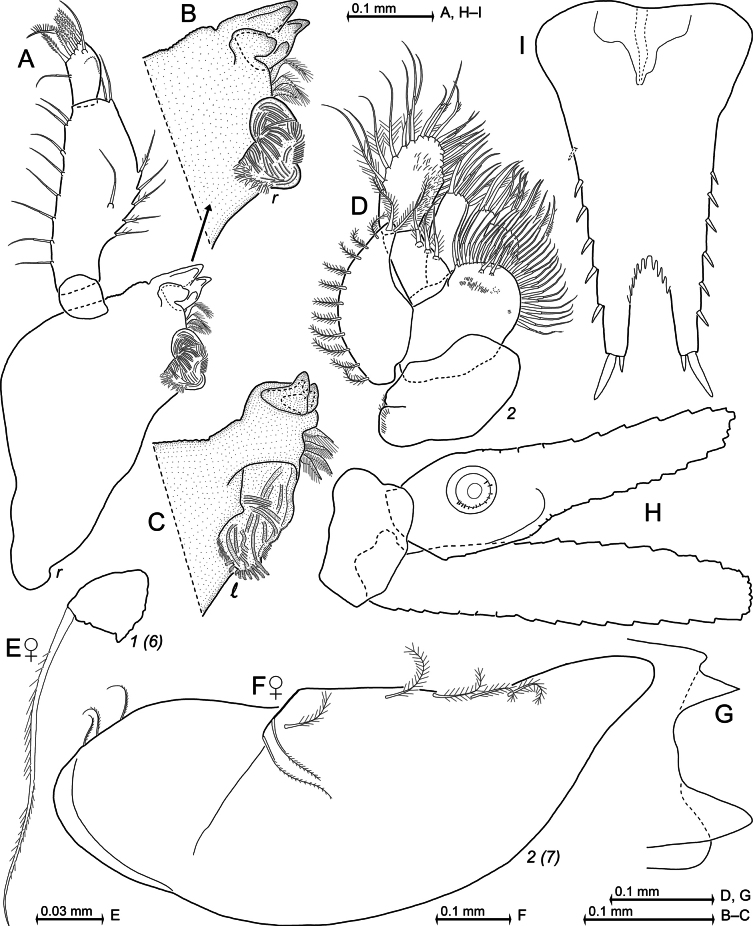
Mouthparts, marsupium and tail fan in *Bermudamysis caribbaea* sp. nov. adult paratypes from Barracuda Cave in Guadeloupe. **A**. Right mandible with palpus, caudal; **B**. Masticatory part of right mandible, caudal; **C**. Masticatory part of left mandible, rostral; **D**. Maxilla, rostral; **E, F**. Oostegites 1 and 2 expanded on slide, inner (mesial) aspect; **G**. Caudal margin of pleon, left lateral aspect; **H**. Left uropods, dorsal, setae omitted; **I**. Telson, ventral.

**Figure 40.**
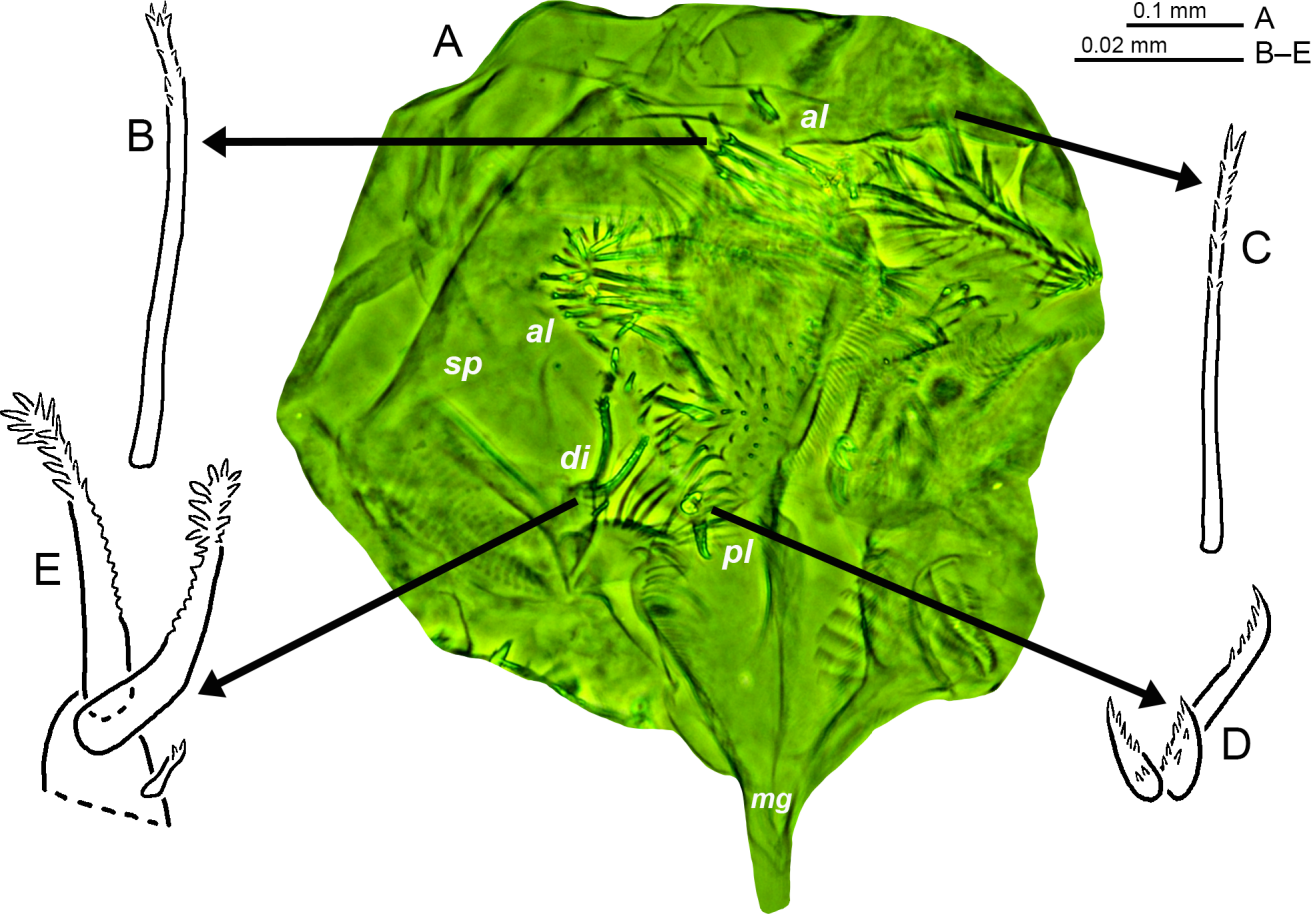
Foregut of *Bermudamysis caribbaea* sp. nov. paratype ♂ from Barracuda Cave in Guadeloupe. **A**. Somewhat malformed foregut, dorsal, object artificially separated from background, lower case labels indicate anterior lateralia (*al*), dorsolateral infolding (*di*), midgut (*mg*), posterior lateralia (*pl*) and storage space (*sp*); **B, C**. Spines of anterior lateralia; **D**. Spine group of posterior lateralia; **E**. Spine group of dorsolateral infolding.

**Figure 41.**
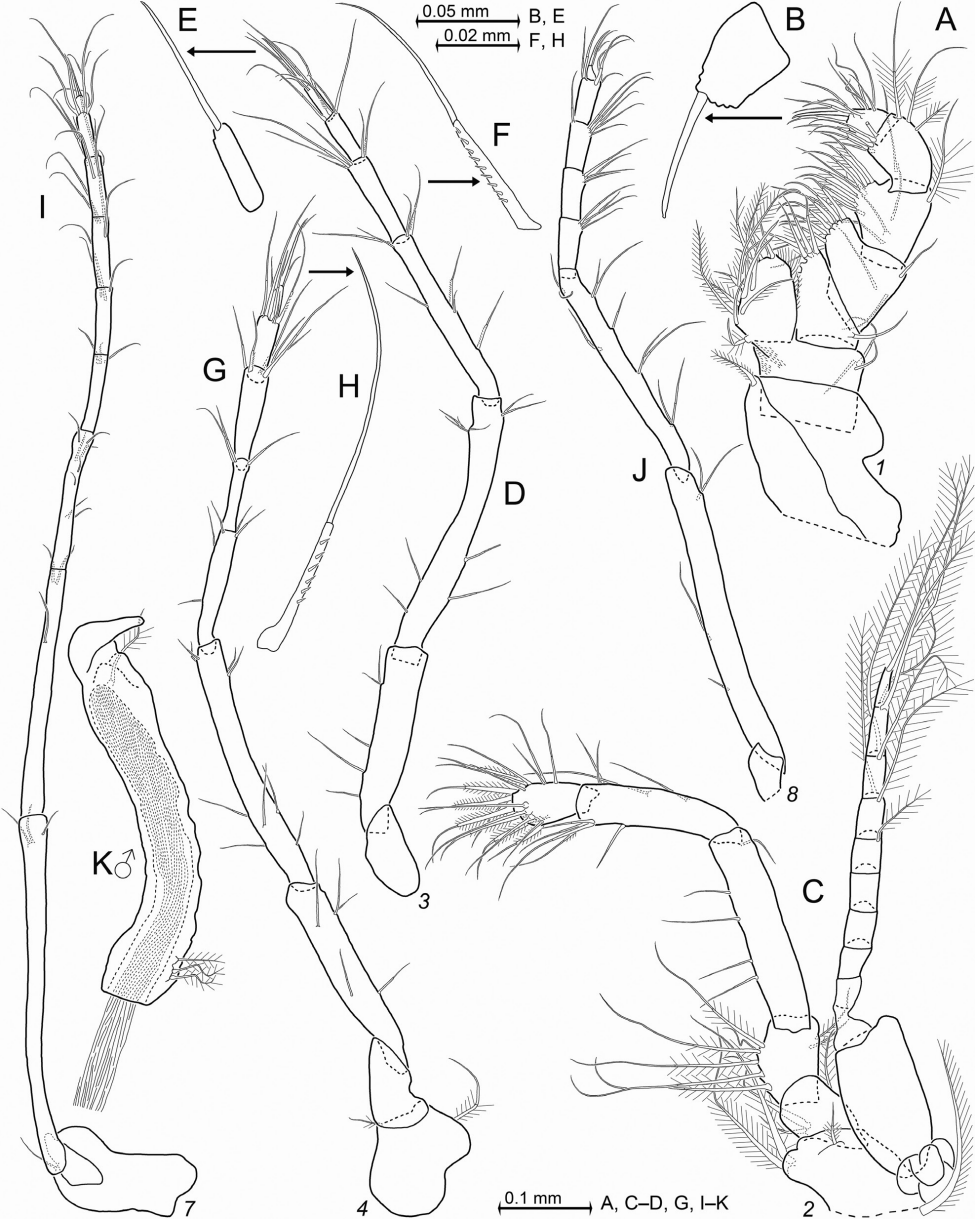
Thoracopods 1–4, 7, 8 and penis of *Bermudamysis caribbaea* sp. nov. adult paratypes from Barracuda Cave in Guadeloupe. **A**. Endopod 1 with part of sympod, rostral; **B**. Detail of (**A**) showing dactylus with nail; **C**. Thoracopod 2; **D**. Endopod 3 from praeischium to dactylus; **E**. Detail of (**D**) showing dactylus with nail; **F**. Detail of (**D**) showing proximally spiny seta on proximal segment of carpopropodus; **G**. Endopod 4; **H**. Detail of (**G**) showing basally spiny paradactylar seta; **I**. Endopod 7; **J**. Endopod 8 from praeischium to dactylus; **K**. Penis. **B, E**. Setae omitted from dactylus.

**Figure 42.**
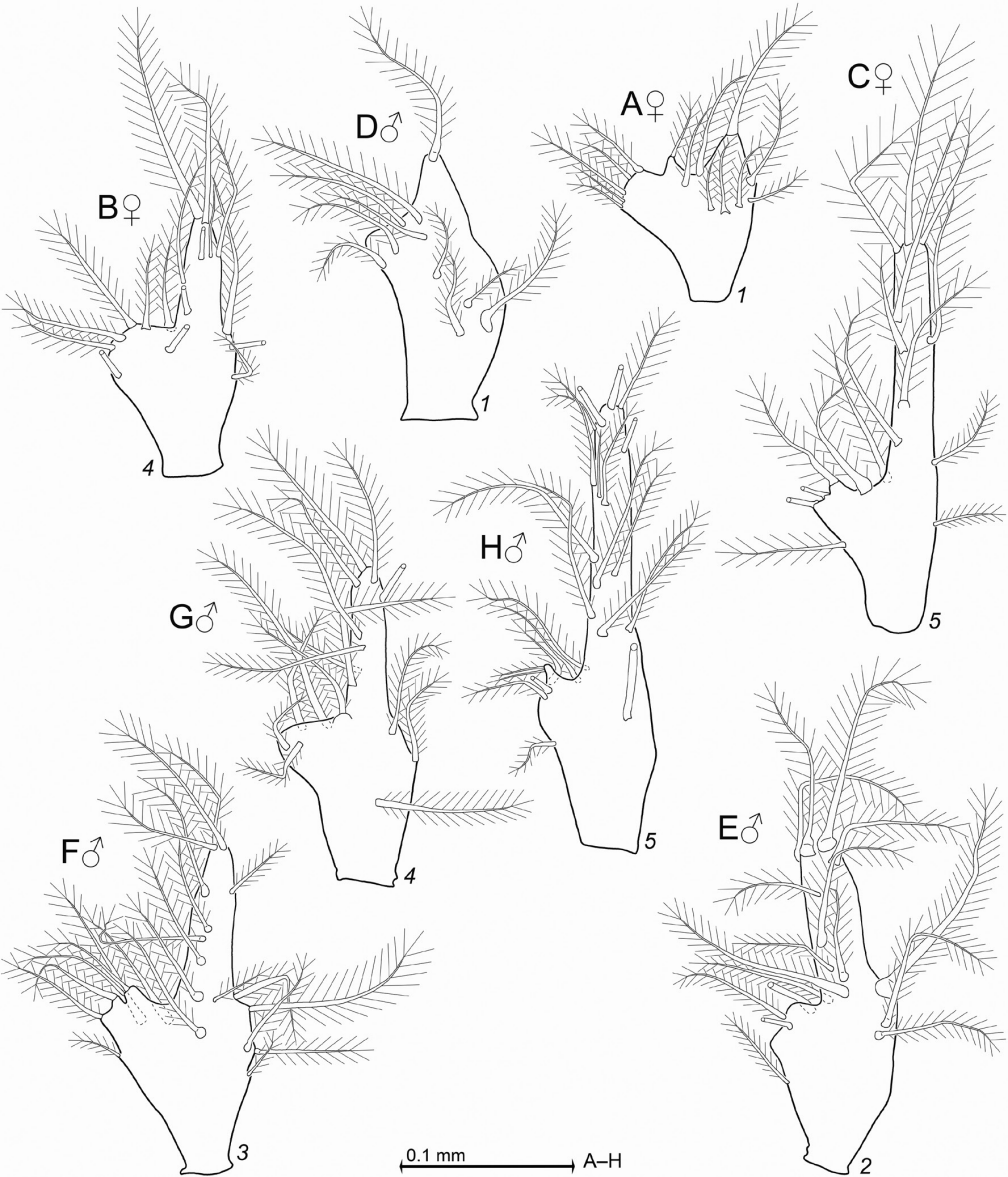
Pleopods of *Bermudamysis caribbaea* sp. nov. adult paratypes from Barracuda Cave in Guadeloupe. **A–C**. Female pleopods 1, 4, 5, ventral = lateral aspect; **D–H**. Male pleopods 1–5, ventral = lateral aspect.

Figs 37, 38, 39, 40, 41, 42

##### Type material.

All types from Lesser Antilles — **Guadeloupe**, Barracuda Cave (Station 11) • ***holotype***, ♂ ad. (BL = 2.6 mm; NHMW-ZOO-CR-31410) • ***allotype***, ♀ ad. (BL = 3.2 mm; MNHN-IU-2025-2825) • ***paratypes***, 1 ♀ ad., 1 ♂ ad. (MNHN-IU-2025-2826) • ***paratypes***, 1 ♀ ad., 1 imm. (NHMW-ZOO-CR-31411) • ***paratypes***, 1 ♀ ad., 1 ♂ ad. (ZMH-K-066758) • ***paratypes***, 3 ♀♀ ad., 1 ♂ ad. (MINGA MYS 456).

##### Diagnosis.

*Bermudamysis* with triangular rostrum, distally narrowly rounded to acute, short, not reaching to half-length of basal segment of antennular trunk; antennal scale reaching to half-length of distal segment of antennular trunk, up to 1/10 its length beyond this segment; scale reaching <1/10 length beyond antennal peduncle; thoracic endopods 3–8 long and slender, endopod 7 the longest, 0.4–0.6× BL; carpopropodus of endopods 3–8 with 3 or 4, 3 or 4, 3 or 4, 4 or 5, 5 or 6 and 4 or 5 segments, respectively; penes tubular, 1/6 BL and 1.2–1.5× length of ischium 8; each lateral margin of telson with six or seven spines only on distal 1/2–2/3; spines weakly increasing in length distally; U-shaped terminal cleft penetrating 24–35% telson length, margins of cleft with nine or ten laminae only on proximal half; distolateral lobes of telson transversely truncate, each with large spine mesially accompanied by a <1/2 as long spine; telson with total of 16–18 spines.

##### Description.

♀♀ ad. with BL = 2.6–3.4 mm (*n* = 11) and ♂♂ ad. with BL = 2.6–2.8 mm (*n* = 5). Cephalothorax comprises 33–38% BL, pleon (without telson) 48–54%, telson 10–14%, carapace (without rostrum) 28–31%, and rostrum 3–5%. Thoracic sternites without mid-ventral processes in both sexes. Pleomeres 1–5 showing 0.7–1.0, 0.6–0.9, 0.7–0.9, 0.6–0.8, and 0.7–0.8× the length of pleomere 6, respectively.

***Carapace*** (Fig. 38D–F). Rostrum as long as 0.5–0.8× length of terminal segment of antennular trunk. Posterior margin of carapace leaves 0.5–1 posterior thoracic somite mid-dorsally exposed. Cervical sulcus very short but distinct. Cervical pore group (Fig. 38E) formed by 11–16 pores with varying diameter (1–3 μm) arranged closely in front of the cervical sulcus. Another pore group (Fig. 38F) closely in front of the mid-posterior margin of carapace; it consists of 10–12 pores ≈1.4 μm in diameter, arranged in subgroups around a larger but indistinct, rounded structure.

***Eyes*** (Fig. 37). Eye length 0.23–0.30 mm (*n* = 11), this is 7–10% BL. OB completely internal, at basis of eyestalk, diameter 34–59 µm (*n* = 4). Cornea calotte-shaped in dorsal view, diameter of calotte 0.20–0.27 mm, length 0.14–0.18 mm, this is 0.6–0.9× length of eyestalk (cornea not included); ovoid in lateral view, length 1.2–1.5× maximum width. Pigment to varying degrees centripetally retracted from crystalline bodies of eye (Fig. 37C), thus feigning a smaller cornea upon low-power microscopy (Fig. 37B, D). According to a review by Land (2004), retreated pigment is indicative of light adaption by pigment migration (a slow process) in crustaceans with superposition eyes.

***Antennulae*** (Fig. 38A, B). Antennular trunk of both sexes with setose apophyses and flagella, no spines, no teeth. Trunk extends 36–57% its length beyond eyes. Measured along dorsal midline, the basal segment is 47–51% trunk length, median segment 13–20%, and terminal segment 31–39%. Basal segment on basal half of its outer face with three or four small, barbed setae. Its dorsal apophysis with two or three simple barbed setae and 1–3 smooth whip setae. The slender lateral apophysis (lobe) reaching to distal margin of median segment or shortly beyond; its transverse terminal margin with three or four barbed setae and none or one smooth seta. Median segment dorsally with bifid apophysis bearing four or five barbed setae; mesial branch of this apophysis longer in males than in females. Terminal segment 0.9–1.1× as long as wide; mesial half with three or four large plumose setae in females only. Mid-dorsal apophysis with 3–5 barbed setae. In both sexes outer antennular flagellum is thicker than inner one. Male lobe well setose, somewhat longer than wide, 1/3 as long as terminal segment of trunk, inserts ventrally close to terminal margin of antennular trunk.

***Antennae*** (Fig. 38C). Antennal sympod with two longer than basally wide, anteriorly projecting lobes between scale and peduncle. Antennal scale length is 3.1–4.3× maximum width; apical segment contributes 12–16% to total length; scale reaching 3–10% of its length beyond antennal peduncle. Basal segment contributes 18–21% to total length of peduncle, median segment 40–43%, and terminal segment 38–42%.

***Primary mouthparts*** (Figs 38G, 38H, 39A–C). Labrum (Fig. 38G) not produced into a spiniform process; caudally weakly rugged and with small, stiff bristles; fields of setae on caudal and oral faces. Left and right mandibles (Fig. 39A–C) essentially alike. Proximal segment of palp 11–18% palp length, no setae. Length of median segment 2.1–2.6× its maximum width and 63–70% palp length. Mesial margin of median segment with 4–6 mostly sparsely barbed whip setae in near-median position, lateral margin almost all along with 6–9 smooth whip setae, terminal margin with two smooth whip setae. Terminal segment distally well setose, 19–23% palp length. Pars molaris with well-developed grinding surface. Pars incisiva with 2–4 teeth, digitus mobilis with two or three teeth, and pars centralis with three or four spines bearing stiff bristles. Labium (Fig. 38H) normal, comprising two hairy lobes with dense set of stiff bristles on distal third of inner face, no spines.

***Foregut*** (Fig. 40). Gross structure as described by Wittmann and Abed-Navandi (2019) for *Heteromysis domusmaris*. Lateralia anteriorly with apically coronate spines (Fig. 40B) and apically pronged spines (Fig. 40C), each with loose series of small denticles along distal 1/5–1/3. Lateralia more caudally with separate group of three unilaterally serrated spines (Fig. 40D). Dorsolateral infoldings with spine group formed by a small apically toothed spine together with two long spines, the latter furnished with unequal-sized teeth along distal 2/3 of shaft (Fig. 40E). Two of three dissected foreguts were almost empty, the third one filled to ≈20% with copepod remains, mostly unidentified organic material and few mineral particles.

***Maxillula*** (Fig. 38I) normal; distal segment terminally with 10–12 mostly weakly serrated spines; subterminally with four whip setae barbed on their distal 2/3; no pores detected. Endite of maxillula terminally with three strong, distally spiny setae accompanied by two barbed whip setae; inner and outer margins with numerous shorter, barbed whip setae.

***Maxilla*** (Fig. 39D) normal; terminal segment of endopod 1.7–1.9× as long as maximum width. Lateral margin of exopod all along with 10–13 plumose setae, the two apical setae 2–3× longer than the lateral setae.

***Thoracopods*** (general, Figs 37D, 41). Both sexes with flagellum of thoracic exopod 1 with eight segments, flagella 2–8 with nine segments. Basal plates expanded, 1.9–2.5× as long as wide in both sexes, distolateral corner well rounded (Fig. 41C). First thoracopods with leaf-like, smooth epipod. A large plumose seta present at the intersegmental joint connecting sympod 2 with the corresponding thoracic sternite; no such setae detected in remaining thoracopods. Total length of endopods, including length and slenderness of ischium, increase in series of endopods 1–7 and then decrease to endopod 8. Endopod 3 (Fig. 41D) measures 1/3 BL and shows somewhat thicker and shorter ischium compared with endopods 4–8. Endopods 3, 4, partly also endopod 5, with one basally spiny (Fig. 41H) and several smooth paradactylar whip setae. Endopod 3 with additional basally spiny setae (Fig. 41F) on merus and carpopropodus, none on praeischium and ischium. Endopods 4, 5 only with smooth setae from praeischium to merus; endopods 6–8 all along with smooth setae. Endopod 1 with smooth, slender, weakly bent nail (Fig. 41B), endopod 2 without nail, endopods 3–8 with smooth, even more slender, weakly bent to straight nail (Fig. 41E). Nail 3 longer than nail 1, length then decreasing in series of nails 3–8. When stretched anteriorly, endopod 8 reaches to antennae, when stretched posteriorly to pleopod 5.

***Maxillipeds*** (Fig. 41A–C). Coxa of first maxilliped (thoracic endopod 1, Fig. 41A, B) with small endite bearing one barbed seta at tip. Basis with large, prominent endite that is densely setose on mesial margin. Ischium and merus each with one smaller, mesially setose endite. Basis of second maxilliped (thoracic endopod 2, Fig. 41C) with mesially projecting endite bearing one or two large plumose seta. In both sexes, combined praeischium plus ischium 0.5–0.7× length of merus, carpopropodus plus dactylus 1.0–1.2× merus. Dactylus very large, with dense brush formed by large numbers of normal setae and 7–9 modified setae, the latter apically bent, bearing two symmetrical series of stiff cils on either side in subbasal to median portions.

***Marsupium*** (Figs 37B, 39E, 39F). Gross structure of marsupium as described above for *B. speluncola*. The vestigial oostegite 1 (Fig. 39E) with only one seta penetrating into marsupial chamber, this seta ‘hispid’ by bilateral series of minute cils along subbasal to subapical portions. Caudal third of oostegite 2 (Fig. 39F) with several hispid setae, rostral third with bilaterally well-barbed setae, central third with hispid as well as barbed setae. Oostegite 3 with only few hispid setae near conjunction with sympod; most margins of the (folded) plate with numerous setae barbed at least along distal 2/3. No setae on outer face of marsupium.

***Penes*** (Figs 37D, 41K) 1.1–1.4× length of ischium 7, when stretched anteriorly extending up to thoracic sympods 2–4. Tip trilobate. Penes subapically with one barbed seta, subbasally with group of three shorter barbed setae.

***Pleopods*** (Fig. 42). Structure as in the above genus definition. Pleopod 1 shorter than the subequal pleopods 2–4, pleopod 5 longest. Male pleopods slightly longer than corresponding female pleopods. No additional differences between sexes.

***Tail fan*** (Fig. 39G–I). Scutellum paracaudale triangular, tip acute (Fig. 39G) to narrowly rounded. Exopod of uropods 1.1–1.2× length of endopod (Fig. 39H). Exopod extends by 4–9% its length beyond endopod and 29–33% beyond telson; endopod 20–24% its length beyond telson. Diameter of fluorite statoliths 55–71 μm (*n* = 6). Statolith formula 2 + 3 + (2–3) + 3 + 2 = 12–13 or 2 + 3 + (2–3) + 5 = 12–13 (*n* = 4 statoliths from 3 specimens). Latero-apical spines of telson (Fig. 39I) 12–15% telson length; medio-apical spines 0.3–0.4× the length of latero-apical spines. Telson length 1.3–1.7× its maximum width or 1.1–1.4× length of sixth pleomere.

##### DNA data.

Only one 629 bp mitochondrial COI sequence obtained from a single individual at Barracuda Cave (GenBank PV990330).

##### Derivatio nominis.

The species name is a Latinized adjective with feminine ending, derived from a Kalinago language noun, referring to occurrence in the Caribbean.

##### Distribution and habitat

(Fig. 10A). Known only from the type locality in the marine sublittoral Barracuda Cave (Station 11), Guadeloupe. The mysids were observed occurring in small groups hovering above the sediment of semi-dark parts in the cave. They were reddish *in situ* and co-occurred with *Parvimysis laminata*. The observed retreated eye pigment (Fig. 37C) correlates with occurrence in light rather than dark parts. The species is classified as a troglophile based on its occurrence in somewhat lighter rather than very dark cave parts in combination with large functional eyes.

##### Remarks.

The new species differs from its only congener *B. speluncola* by carpopropodus of thoracic endopods 6–8 with 4–6 segments (vs three segments), carpopropodus of endopod 3 with three or four segments (vs two or three segments), and penes 1.2–1.5 × (vs 0.4–0.6 ×) as long as ischium 8.

### Tribe Heteromysini Norman, 1892

#### Chelitrapezura gen. nov.

Taxon classificationAnimaliaMysidaMysidae

C45B1787-67DA-5B25-9FBA-AFD2028E2D7B

https://zoobank.org/33FD3ACD-0132-445A-9E7E-779D20EB0FBD

Fig. 43

Heteromysoides : Bowman 1985: 945 (partim; first description of stygobiont species).
Platyops : Daneliya 2021: 4 (partim; reassignment of Heteromysoides species).

##### Type species.

*Heteromysoides dennisi* Bowman, 1985, by present designation. The combination *Platyops dennisi* (Bowman, 1985) emend. Daneliya 2021, here recombined as *Chelitrapezura dennisi* (Bowman, 1985), comb. nov. This species so far known only from the type locality at Grand Bahama Island, Cemetery Cave.

##### Derivatio nominis.

The genus name is a noun with feminine ending, formed by fusion of the Greek nouns χηλή (claw), τραπέζιο (trapeze) and ουρά (tail), referring to the subchelate thoracic endopod 3 in combination with the trapezoid telson as the main diagnostic characters.

##### Diagnosis.

Heteromysini with large, subquadrangular eyestalks, no eye papilla, small cornea implanted laterally; antennular trunk with normal setae, no flagellate spines, no backward-directed modified setae; antennal scale, pleopods and uropods setose, no spine, no teeth; thoracic endopod 3 forming a stout subchela with thick fused carpopropodus mesially armed with spines; thoracic endopods 4–8 normal, long, slender, with 2- to 4-segmented carpopropodus, praeischium without distal projection, ischium and merus without flagellate spines; penes tube-like; pleopods reduced to unsegmented setose plates with inconspicuous rudiments of pseudobranchial lobes; uropods normal, both rami undivided; telson trapezoid with bare proximal half, only distal ≤1/2 of lateral margins and terminal margin with spines.

##### Remarks.

**Figure 43.**
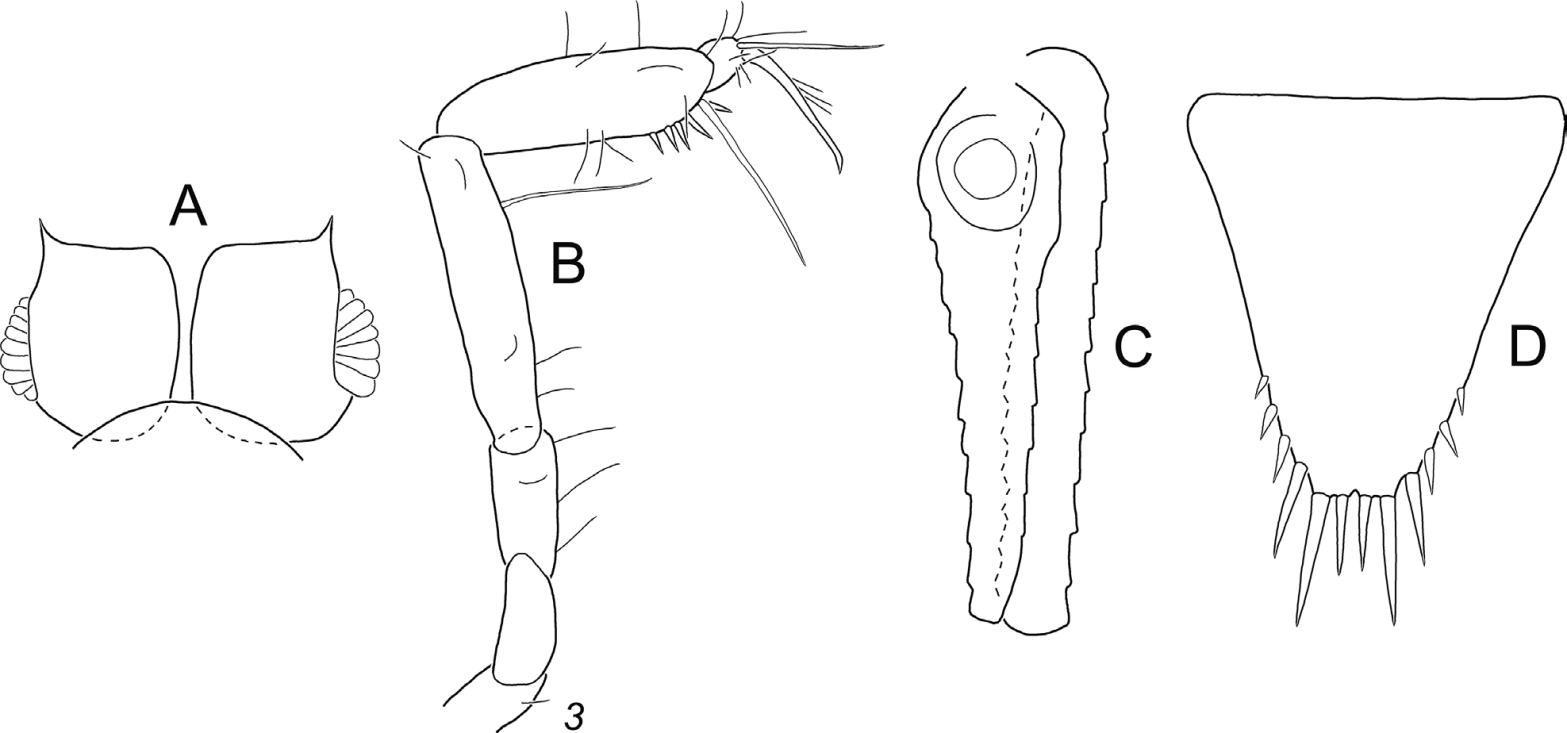
Selected diagnostic characters of *Chelitrapezura dennisi* (Bowman). **A**. Eyes, dorsal; **B**. Thoracic endopod 3; **C**. Right uropods, dorsal, setae omitted; **D**. Telson. Modified after Bowman 1985.

Due to its prehensile thoracic endopod 3 with swollen unsegmented carpopropodus mesially armed with spines, *Platyops dennisi* (Bowman, 1985) or (as originally) *Heteromysoides dennisi* Bowman, 1985, respectively, cannot remain in the genus *Platyops*. The combination of a strong prehensile endopod 3 and trapezoid telson does not fit with any currently known genus of the subfamily Heteromysinae. This combination points to morphologically based paraphyly that arose from the integration of *Heteromysoides dennisi* into *Platyops* based on failed assumptions (as shown above) about the morphology of the type species *P. sterreri* by Daneliya (2021). This problem is addressed here by installing the new genus *Chelitrapezura* with *Heteromysoides dennisi* Bowman, 1985, as type species, this taxon then recombined as *Chelitrapezura dennisi* (Bowman, 1985), comb. nov. The new genus differs from the four other genera of the tribe Heteromysini and the single genus of the tribe Harmelinellini (namely *Harmelinella* Ledoyer, 1989) by a terminally truncate telson without cleft (Fig. 43D). It differs from all seven currently known genera of Mysidetini by the subchelate thoracic endopod 3 with fused, mesially spinose carpopropodus (Fig. 43B). Considering the modification of endopod 3 as a more complex structure than telson shape, the genus *Chelitrapezura* is here affiliated to the Heteromysini.

#### Chelitrapezura dennisi

Taxon classificationAnimaliaMysidaMysidae

(Bowman, 1985) comb. nov.

BE061DF6-4581-5800-83CC-3D20EAA82428

Fig. 43

Heteromysoides dennisi Bowman, 1985; Juberthie-Jupeau and Iliffe 1994: Table 1 (stygobiont, Blue Holes in Bahama Islands); Hanamura and Kase 2001b: 70 (in key); Daneliya 2021: 4 (taxonomy).
Platyops dennisi : Daneliya 2021: 4, 45–46 (revised combination).

##### Distribution

(Fig. 10B). Known only from the type locality at Grand Bahama Island, Cemetery Cave, ca 100 m off the coast in a large blue hole, in 17 m depth, 330 m from entrance (as *Heteromysoides dennisi* in Bowman 1985, and in Juberthie-Jupeau and Iliffe 1994).

##### Short diagnosis.

Rostrum evenly rounded; distolateral edges of eyestalks anteriorly extended into a tooth-like projection, penes with long apical seta; endopod and exopod of uropods (sub)equal in length, no spines; telson slightly longer than maximum width near basis, distal ≤1/2 of each lateral margin with four spines increasing in length distally, terminal margin with pair of large disto-lateral spines flanking two half as long paramedian spines.

#### Genus Heteromysis

Taxon classificationAnimaliaMysidaMysidae

S.I. Smith, 1873

DE23E4D5-FFFA-5E4D-9276-B06D43EB9FAD

Figs 44, 45, 46, 47, 48, 49, 50, 51

Heteromysis S.I. Smith, 1873: 553, 554 (original description); G.O. Sars 1883: 55 (synonymy); Daneliya 2021: 6–8 (revision, key to subgenera).
Chiromysis G.O. Sars, 1877: 56, 57 (original description of junior synonym); G.O. Sars 1883: 55 (in synonymy).
nec Heteromysis Czerniavsky, 1882a: 57 (homonym of Heteromysis S.I. Smith, diagnosis in key); Czerniavsky 1882b: 33 (diagnosis).

##### Short diagnosis.

Heteromysini with cornea normal or reduced to various degrees; disto-mesial corner of terminal segment of antennular trunk with or without flagellate spine opposed by a large smooth seta, no backward-oriented modified setae at this corner; thoracic endopod 3 prehensile, mostly stout, with swollen 1- to 2-segmented carpopropodus armed with spines on mesial (= inner) margin; praeischium of thoracic endopod 8 without spiniform extension in both sexes; penes tubular; pleopods reduced to simple setose plates in both sexes, with or without flagellate spines in males; endopod of uropods armed with spines in most species; telson with well-developed cleft at least basally armed with spine-like laminae; lateral margins of telson armed with spines at least in distal portions, terminal margin of disto-lateral lobes with at least one large spine and mostly with additional spines.

**Figure 44.**
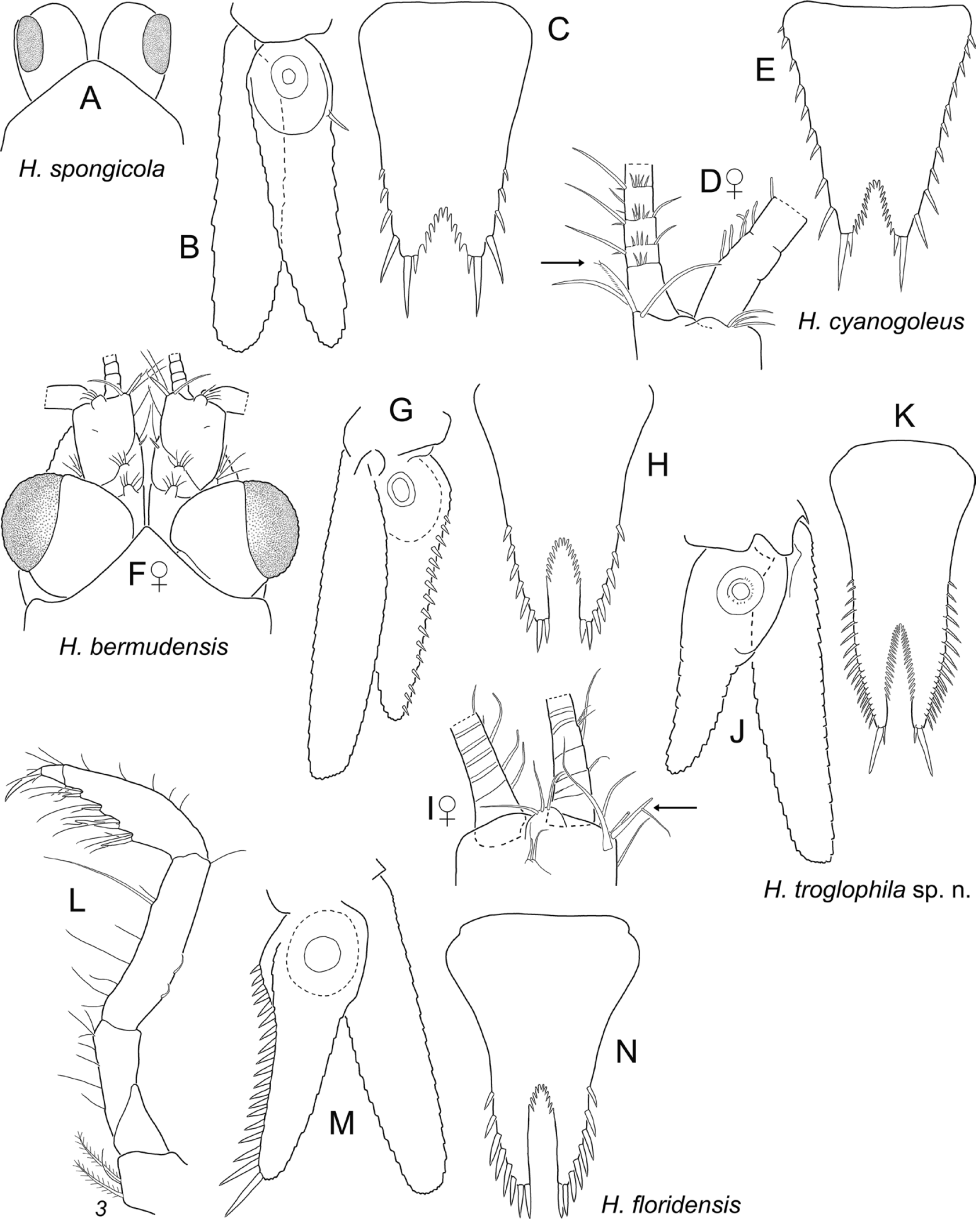
Differentiation between the species of *Heteromysis* S.I. Smith, from Caribbean caves. **A–C**. Eyes and anterior margin of carapace (**A**), left uropods, dorsal (**B**), and telson (**C**) in *H. spongicola* (Băcescu); **D, E**. Distal portion of antennular trunk (**D**, arrow points to apically bi-flagellate seta), and telson (**E**) in *H. cyanogoleus* Bamber; **F–H**. Cephalic region of female, dorsal (**F**), right uropods, ventral (**G**), and telson (**H**) in *H. bermudensis* G.O. Sars; **I–K**. Distal portion of left antennular trunk (arrow points to subapically flagellate spine), dorsal (**I**), right uropods, dorsal (**J**), and telson (**K**) in paratype ♀ of *H. troglophila* sp. nov.; **L–N**. Thoracic endopod 3 (**L**), right uropods, dorsal (**M**), and telson (**N**) in *H. floridensis* Brattegard. Modified after Băcescu 1968 (**A–C**), Bamber 2000 (**D, E**), Brattegard 1973 (**F–H**), Brattegard 1969 (**L–N**); present contribution (**I–K**).

##### Species inventory.

Five species from Caribbean caves are here treated and given in below key to species: H. (Heteromysis) cyanogoleus Bamber, 2000, H. (Olivemysis) bermudensis G.O. Sars, 1885, H. (O.) floridensis Brattegard, 1969, *H. spongicola* (Băcescu, 1968), and *H. troglophila* sp. nov. Additional 103 species are acknowledged at world-wide scale. Type species is *Heteromysis formosa* S.I. Smith, 1873.

##### Remarks.

Băcescu (1968) did not explicitly designate a type species upon installing the genus-group taxon *Heteromysoides*. He added two species to this genus by using the wordings “*Heteromysoides spongicola* n.g. n.sp.” (in abstract and in legend of fig. 5) and “*Heteromysoides cotti* (Calman, 1932)” (on p. 231). From this, Daneliya (2021) argued that it would be impossible to determine which of the two species could be the type species and so the name ‘*Heteromysoides*’ would be unavailable by not matching Art. 13.3. of the Nomenclatorial Code (ICZN 1999). However, another article, namely Art. 13.4. is very well matched by inclusion of a single new species marked by “n.g. n.sp.”, thus deeming to confer availability. In conclusion, the type species was fixed from the very beginning as *Heteromysoides spongicola* Băcescu, 1968.

#### Heteromysis spongicola

Taxon classificationAnimaliaMysidaMysidae

(Băcescu, 1968)

F9825443-A175-5D02-81B0-3E4FEC8C56C0

Figs 44A–C, 45

Heteromysoides spongicola Băcescu, 1968: 231–233, fig. 5 (original description from Cuba); Pesce 1982: 113, 115 (in list of cave-dwelling taxa); Escobar-Briones and Soto 1991: Table 1 (biogeography); Hanamura and Kase 2004: 2146, 2147, fig. 1 (first record from Caribbean cave, redescription); Petrescu and Wittmann 2009: 58 (in type collection); Daneliya 2021: 3, 4 (type species, synonymy).
Heteromysis spongicola : Daneliya 2021: 4 (revised combination); Wittmann et al. 2021: 516 (taxonomy).

##### Type material examined.

**Figure 45.**
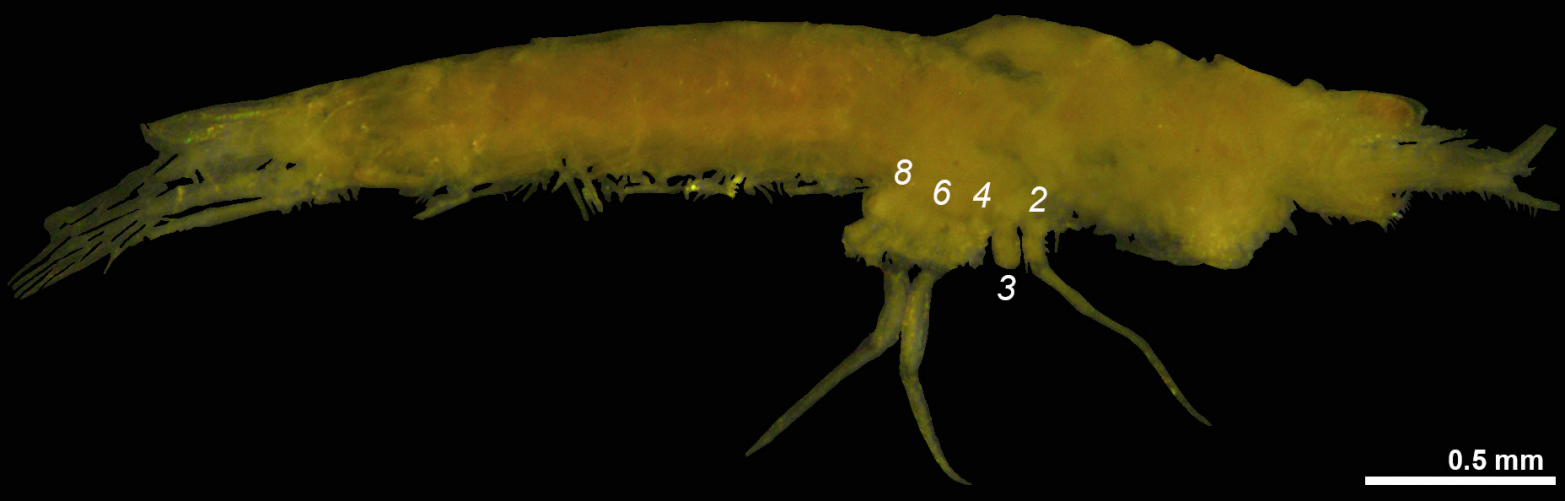
Habitus of *Heteromysoides spongicola* Băcescu (now *Heteromysis spongicola*) from sponge at the Gulf of Mexico coast of Cuba, paratype damaged ♀, lateral, numerals denote series of thoracic sympods 2–8.

Cuba • ***holotype***, ♂ ad. (BL ≈3.5 mm, TL ≈4.2 mm; MINGA 49009) broken in two damaged pieces; from sponges in stands of corals (Station 25: type locality) • ***paratype***, damaged ♀ ad. (BL = 2.9 mm, TL = 3.5 mm; MINGA MYS 454; Fig. 45); sampling data as for holotype. The holotype listed as female in Petrescu and Wittmann (2009).

##### Diagnosis.

*Heteromysis* with ovoid, dorsally flattened eyestalks and with small, laterally implanted cornea; distal 1/5 of the stalks obliquely truncate in the sagittal plane, rendering the eye anteriorly chisel-shaped in lateral view, not so in dorsal view; disto-mesial corner of the antennular trunk with normal setae only, no strongly modified seta; pleopods reduced to simple setose plates in both sexes, no teeth, no spines; endopod of uropods with only one spine mesially implanted below statocyst; telson trapezoid with incised terminal margin; distal half of lateral margins with four spines increasing in length distally, proximal half bare; disto-lateral lobes narrowly truncate, each armed with large spine mesially accompanied by a very small spine; triangular cleft armed all along its margin with nine or ten acute laminae.

##### Descriptive notes.

***Female paratype*** (Fig. 45). No ventral hump on distal segment of antennular trunk. Eyestalk subrectangular with rounded distal margin, 1.5× longer than broad in dorsal view. Eyestalk dorsoventrally compressed by a factor of 1.8, dorsally more strongly flattened than ventrally. Thoracic endopods 1–8 and most pleopods broken. Thoracic sympod 3 thicker than sympods 2, 4–8 (Fig. 45), most likely supporting a thick, potentially prehensile endopod in analogy to other species of *Heteromysis*. Terminal cleft penetrates 1/5 telson length. Each lateral margin of telson with four spines increasing in length distally.

***Male holotype***. Antennular trunk extends 0.3× its length beyond antennal scale. Distal segment of trunk ventrally with short papillary hump, possibly homologous with the otherwise missing appendix masculina. Antennal scale 3× longer than broad. Eye dorsoventrally compressed by a factor of 2.0 in lateral view, 1.4× longer than broad in dorsal view. Penes tube-like with 11% BL, no apical stylet (seta). Pleopod 3 without setae measures 1.5% BL, so reaching to half-length of pleomere 4; pleopod 4 is 1.8% BL, reaching to proximal 1/4 of the longer pleomere 5.

##### Distribution

(Fig. 10D). So far recorded from sponges in stands of corals at the northern coast of Cuba (type locality) and from a submarine cave off the western coast of Grand Cayman Island (Caribbean), total range 19–23°N, 81–82°W, 9–26 m depth (Băcescu 1968; Hanamura and Kase 2004). Both records are from cryptic habitats.

##### Remarks.

Upon synonymization of the genus *Heteromysoides* Băcescu, 1968, Daneliya (2021) proposed as a provisory to recombine the poorly known type species *Heteromysoides spongicola* Băcescu, 1968, as *Heteromysis spongicola* (Băcescu, 1968). Our external inspection of the strongly damaged female paratype of *Heteromysoides spongicola* at the MINGA yielded a previously unknown thickening of thoracic sympod 3 (Fig. 45) pointing to a strong (broken) endopod 3. This would exclude attribution to the genus *Mysidetes* Holt & Tattersall, 1906, thus indirectly (though weakly) supporting the recombination of this species taxon with the genus *Heteromysis* S.I. Smith, 1873, by Daneliya (2021).

#### Heteromysis (Heteromysis) cyanogoleus

Taxon classificationAnimaliaMysidaMysidae

Bamber, 2000

0A3B6FAA-9DA7-5092-A563-A97F10BB8FDF

Fig. 44D, E

Heteromysis cyanogoleus Bamber, 2000: 129–133, figs 1–3 (original description, Bahamas); Hanamura and Kase 2001a: 17 (discussion on subgeneric assignment); Fukuoka 2005: 1363, table III (habitat, distribution).

##### Diagnosis.

Modified after Bamber (2000). *Heteromysis* with pointed triangular rostrum extending beyond basal segment of antennular trunk; disto-mesial edge of antennular trunk in addition to normal setae with one blunt seta, mesially finely barbed and with distal pair of minute flagella (arrow in Fig. 44D); eyes normal, cornea large, calotte-shaped, distally attached on the eyestalk; merus and carpus of the strong, subchelate thoracic endopod 3 mesially with flagellate spines; pleopods reduced to simple setose plates in both sexes, no teeth, no spines; endopod of uropods with only one spine mesially located beneath statocyst; telson trapezoid with incised terminal margin, lateral margins all along with nine or ten spines, disto-lateral lobes narrowly truncate, each armed with large spine mesially accompanied by a very small spine; triangular cleft armed all along with 18 acute laminae.

##### Affiliation.

Pleopods without spines together with modified seta at the disto-mesial corner of the antennular trunk bearing small apical flagella rather than a spine with subapical flagellum point to the subgenus *Heteromysis* S.I. Smith, 1873.

##### Distribution

(Fig. 10E). Sandy’s Cave, east end of Grand Bahama Island, ca 27°N, 78°W (Bamber 2000). The species provisionally considered troglophile.

#### Heteromysis (Olivemysis) bermudensis

Taxon classificationAnimaliaMysidaMysidae

G.O. Sars, 1885

ECA752DC-8A5C-544A-B455-714EEE02884C

Fig. 44F–H

Heteromysis bermudensis G.O. Sars, 1885: 216, figs 1–7 in pl. XXXVIII (original description, Bermuda); Clarke 1955: 5, fig. 7 (description, Bermuda); Brattegard 1973: 51–53, fig. 20 (description, Caribbean); Modlin 1987a: 116 (records, Caribbean); Fukuoka 2005: table III (habitat, distribution); Price and Heard 2008: Table 1 (morphology); Wittmann 2023: table SS1 (records, trogloxene status).
Heteromysis bermudensis bermudensis : Bowman 1981b: 458–461, fig. 1 (first description of male).
Heteromysis (Olivaemysis) bermudensis : Băcescu and Iliffe 1986a: 99, 100, fig. 2M–R (description of male, misspelling of subgeneric name).
Heteromysis (Olivemysis) bermudensis : Price et al. 2002: 47, figs 3, 4 (records, in key); Ortiz et al. 2017c: 49 (record, Yucatán); Wittmann and Abed-Navandi 2021: 137, 141 (morphology, in key).

##### Material examined.

Leeward Antilles — **Curaçao** • 1 ♂ subad. (BL = 3.1 mm) inside marine cave at Playa Lagun (Station 19).

##### Diagnosis.

*Heteromysis* with triangular rostrum covering only basal part of eyestalks; eyes normal, cornea large, calotte-shaped, terminally attached to the eyestalk; disto-mesial edge of antennular trunk with modified spine characterized by a flattened handle with thin, subapically inserting flagellum; antennal scale with small distal segment, not reaching beyond distal margin of antennular trunk; thoracic endopod 3 subchelate, strong, with six or seven strong flagellate spines on mesial margin of fused carpopropodus; pleopods reduced to simple setose plates in both sexes, only male pleopod 4 distally with 27–55 densely set, small flagellate spines (modified setae); endopod of uropods not curved, its mesial margin with 10–16 subequal spines between statocyst and terminus; telson trapezoid with incised terminal margin; proximal 3/5 with bare lateral margins, distal 2/5 of lateral margins with 6–8 spines distally weakly increasing in length; disto-lateral lobes narrowly truncate, each armed with large spine mesially accompanied by a 0.6–0.8× as long spine; U-shaped telson cleft with margin furnished with 11–20 laminae along basal half.

##### Distribution

(Fig. 10F). Known from Bermuda, Gulf of Mexico, Belize, Caribbean coast of Mexico (Yucatán), Cuba, Caribbean coasts of Colombia, Cayman Islands, Turks and Caicos Islands and Antilles, total range 16–32°N, 63–88°W, depth 0.1–18 m. Found in marine caves, among algae, in sponges and empty shells, in the spaces between stones, over detritus bottoms in shallow water, also in association with sea anemones (Brattegard 1973; Băcescu and Iliffe 1986a; Modlin 1987a; Price et al. 2002; Fukuoka 2005; Ortiz et al. 2017c; Wittmann 2023). Wittmann (2023) considers this species as trogloxene due to the great dominance of non-cave records.

#### Heteromysis (Olivemysis) floridensis

Taxon classificationAnimaliaMysidaMysidae

Brattegard, 1969

F13D1BE0-04F2-5440-8F56-4B4BF5B3B385

Fig. 44L–N

Heteromysis floridensis Brattegard 1969: 99–103, Table 1, figs 33, 34 (original description, Florida); Modlin 1987b: 299–301, fig. 2 (first record of male, Bahamas); Fukuoka 2005: table III (habitat, distribution).
Heteromysis (Olivemysis) floridensis : Ortiz et al. 2017c: 49 (record, Yucatán); Ortiz and Lalana 2018: 67, 75, fig. 18B (in key, Cuban records).

##### Diagnosis.

Modified and pooled after Brattegard (1969), Modlin (1987b) and Ortiz and Lalana (2018). *Heteromysis* with triangular, distally right-angled, narrowly blunt rostrum covering basal part of eyestalks; eyes normal, cornea of moderate size, calotte-shaped, disto-laterally attached to eyestalk, no tooth-like process on anterior margin of stalk; disto-mesial edge of antennular trunk with flagellate spine characterized by a flattened handle with subapically inserting flagellum; antennal scale unsegmented, short, reaching to half-length of terminal segment of antennular trunk; thoracic endopod 3 forming a stout subchela; the thick fused carpopropodus mesially armed with five or six flagellate spines along its distal 2/3 (not every spine well visualized in Fig. 44L due to arrangement of the distal four spines in pairs); pleopods reduced to simple setose plates in both sexes, only male pleopod 4 distally with 26 densely set, small flagellate spines (modified setae); uropods setose all around, endopod with 13–19 spines along mesial margin between statocyst and terminus, endopod curved mesially; telson linguiform, terminally incised, with bare proximal half of lateral margins, distal half of each margin with 6–8 spines increasing in length distally; disto-lateral lobes narrowly truncate, each armed with large spine mesially accompanied by a slightly longer spine; U-shaped cleft penetrates 0.4× telson length, its margin furnished with 9–11 laminae on proximal quarter.

##### Distribution and habitat

(Fig. 10G). Type locality is Key Biscayne, Florida, 25°42'11"N, 80°11'W, depth 2 m (Brattegard 1969). Known from the Caribbean coast of Mexico (Quintana Roo), Cuba, Florida and Grand Bahama Island, total range 21–27°N, 78–87°W, depth 2–4 m, on sandy bottoms, in sponges and near entrance of Cemetery Cave (Brattegard 1969; Modlin 1987c; Fukuoka 2005; Ortiz and Lalana 2018).

#### Heteromysis troglophila sp. nov.

Taxon classificationAnimaliaMysidaMysidae

24B7E7BA-206E-50E5-9BA6-B41D3757363D

https://zoobank.org/E8F45B34-DC5B-4257-B36D-C477CFF6DD64

Figs 44I–K, 46–51

Heteromysis dispar : Wittmann 2023: table SS1 (eyestalk organs).

##### Type material.

Lesser Antilles — **Martinique** • ***holotype***, ♀ ad. (BL = 4.7 mm; NHMW-ZOO-CR-31424) from Anse Noire Cave (Station 1) • ***paratype***, 1 imm. (BL = 2.4 mm), sampling data as for holotype — **Guadeloupe** • ***allotype***, ♂ ad. (BL = 2.9 mm; NHMW-ZOO-CR-31788), ***paratypes***, 1 imm. (BL = 1.9 mm; NHMW-ZOO-CR-31789), 1 ♀ ad. (BL = 5.1 mm; ZMHK-K-066767) from Amedien Cave (Station 12).

##### Diagnosis.

Based on adults of both sexes. *Heteromysis* with wide-angled broadly rounded rostrum 0.2–1.0× as long as terminal segment of antennular trunk; eyes normal, cornea calotte-shaped with diameter 0.5–0.7× the length of antennular trunk; disto-mesial corner of trunk with flagellate spine characterized by a thick handle with subapically inserting flagellum, no additional modified spines (setae); appendix masculina well-developed; antennal scale with small distal segment, scale reaching to terminus of antennular trunk and 0.2–0.4× its length beyond antennal peduncle; thoracic endopod 2 without nail; carpopropodus 3, 4 with two segments and carpopropodus 5–8 with three segments; thoracic endopod 3 non-dimorphic, subchelate, with carpopropodus 3–4× longer than wide, carpus not fused with propodus, carpus furnished with three flagellate spines along distal 1/7–1/5 of its mesial margin; penes tubular, 1/2 as long as ischium 8, distally with four lobes around ejaculatory opening; pleopods reduced to simple setose plates, no spines in both sexes; uropods setose all around, no spines, endopod not curved; telson linguiform with incised terminal margin; 13–18 spines on convex distal half of each lateral margin, proximal half bare; disto-lateral lobes narrowly truncate with transverse terminal margin armed with large spine mesially accompanied by a much smaller spine and laterally by none or one medium-sized spine; V-shaped cleft penetrates 1/3 telson length, basal 1/2–3/4 of its margin furnished with 26–40 laminae; nauplioid larvae normal, with smooth cuticle all around.

##### Etymology.

The species name is a Latinized adjective with feminine ending referring to the troglophilic habit.

##### Description.

Body length of adult females 4.7–5.1 mm (*n* = 2), the only adult male 2.9 mm; female holotype and male allotype dissected. Cephalothorax comprises 29–34% BL, pleon (without telson) 50–56%, telson 13–14%, carapace (without rostrum) 24–28%, and rostrum 1–4%. Male thoracic sternites 2, 8 with small, acute triangular, ventral processes (Figs 47C, 49G). Potential processes on male sternites 3–7 not clearly identified. No such processes in females. Pleomeres 1–5 show 0.6–0.9, 0.6–0.9, 0.7–0.9, 0.7–0.9, and 0.8–0.9× the length of pleomere 6 in both sexes. Posterior margin of carapace leaves the ultimate thoracic somite mid-dorsally exposed. Cervical sulcus indistinct. Penes (Fig. 50I) treated in Diagnosis.

**Figure 46.**
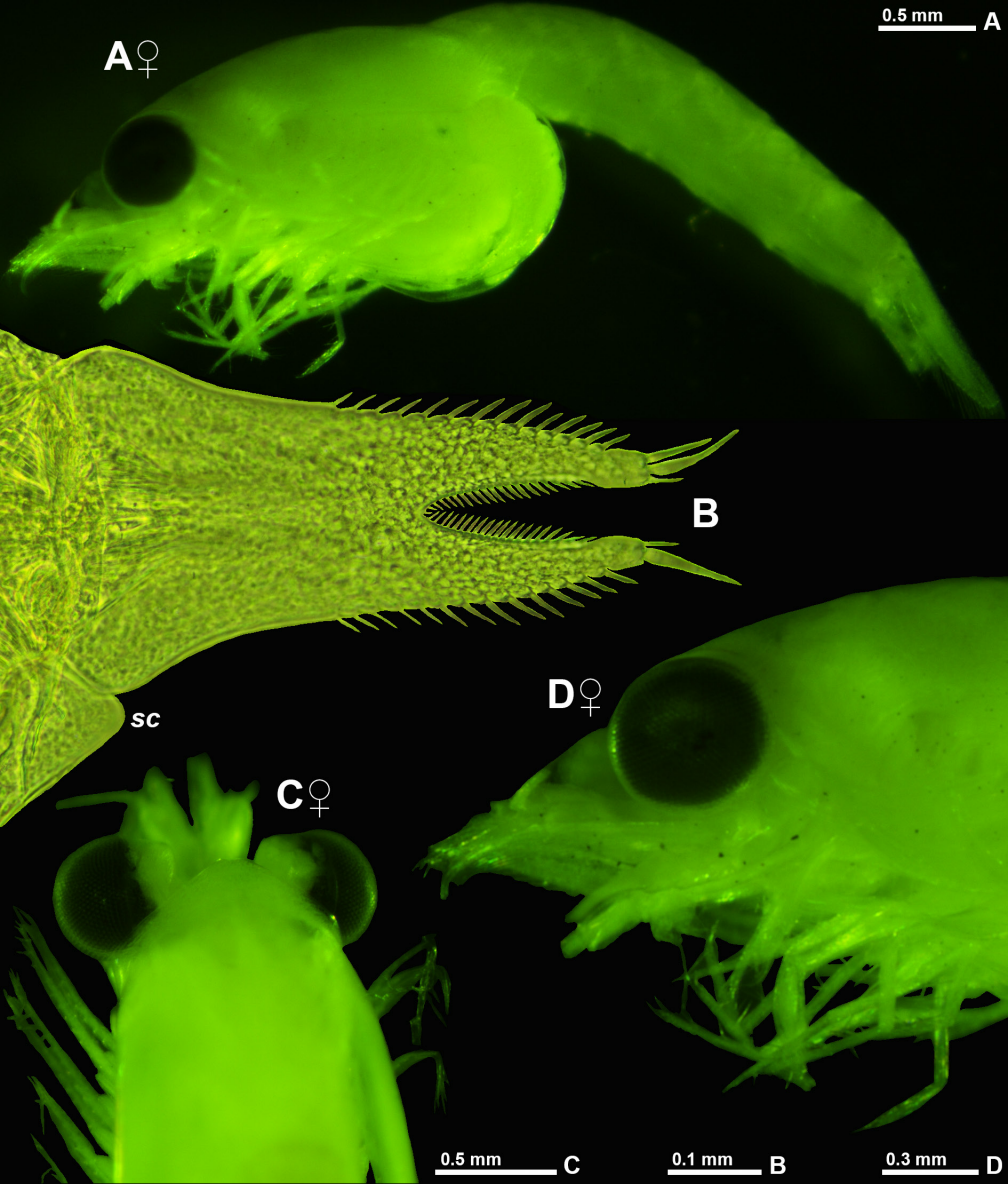
Female habitus and telson in *Heteromysis troglophila* sp. nov. holotype ♀ with nauplioid larvae from Anse Noire Cave in Martinique. **A**. Habitus, lateral; **B**. Telson with left scutellum paracaudale (*sc*), dorsal; **C**. Cephalic region, dorsal; **D**. Cephalic region, lateral. **A–D**. Objects artificially separated from background.

***Eyes*** (Fig. 46A, C, D). Eyes very large, length 0.38–0.71 mm (*n* = 6), this is 11–15% BL. Cornea calotte-shaped in dorsal view, diameter of calotte 0.36–0.52 mm, length 0.27–0.38 mm, this is 0.8–1.3× length of eyestalk (cornea not included); spherical in lateral view. Cornea strongly black pigmented. Eyestalk without lobes, no spiniform processes.

**Figure 47.**
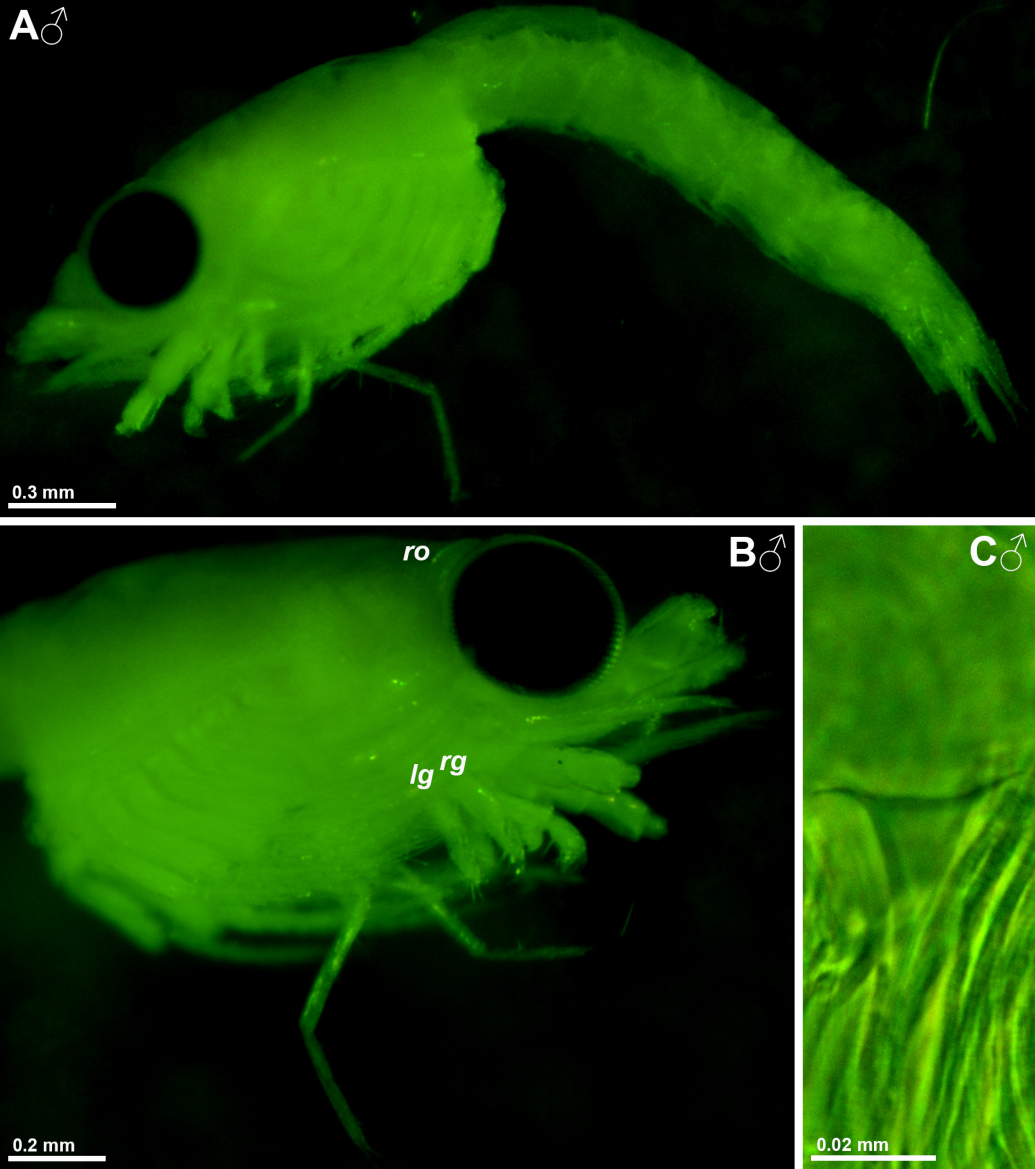
Male habitus and sternal process in *Heteromysis troglophila* sp. nov. allotype ♂ from Amedien Cave in Guadeloupe. **A**. Habitus, left lateral aspect; **B**. Cephalothorax, right lateral aspect, lower-case labels indicate rostrum (*ro*), tarsus of right gnathopod (*rg*, exposing lateral face) and tarsus of left gnathopod (*lg*; exposing dorsal face); **C**. Triangular process from thoracic sternite 8.

**Figure 48.**
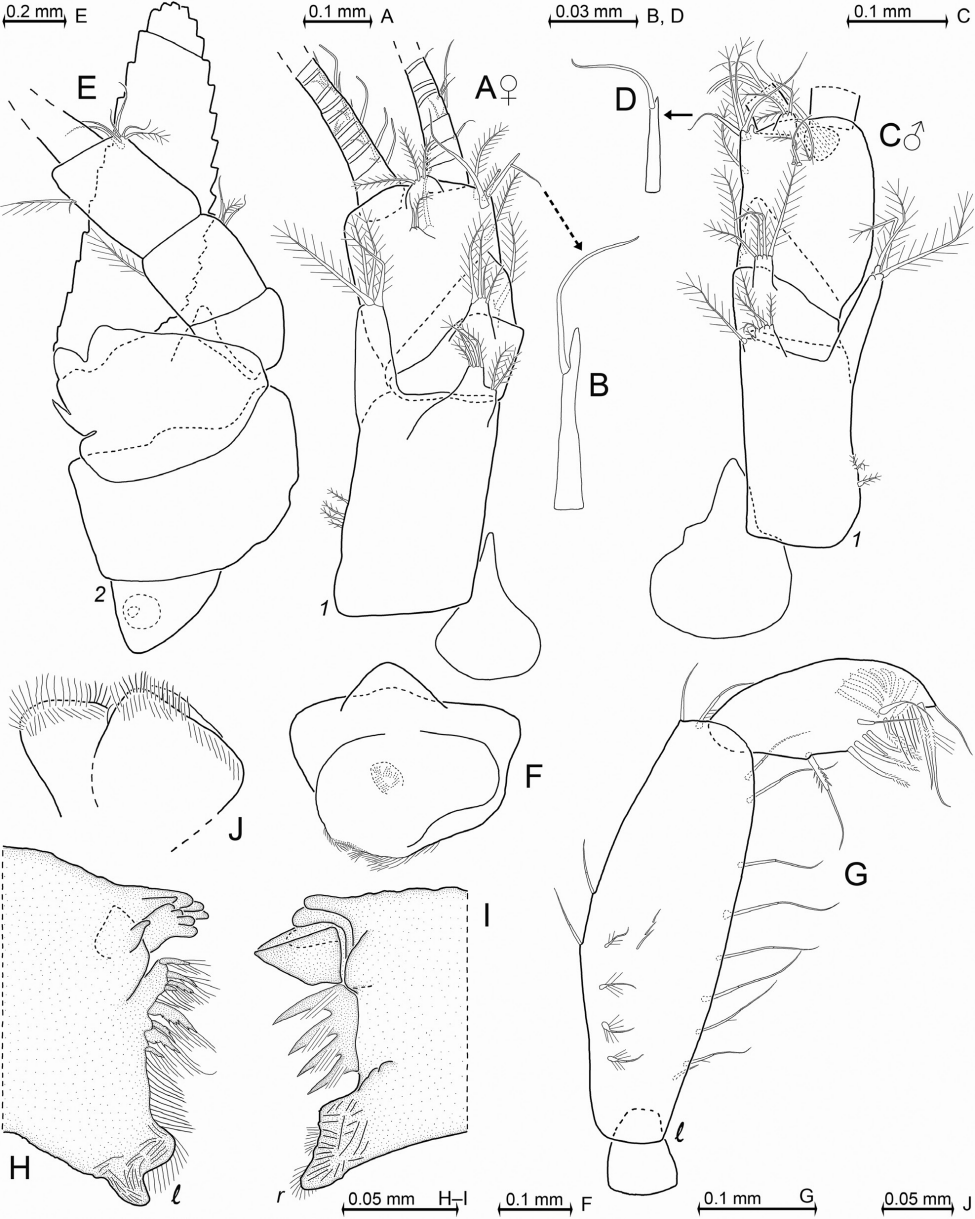
Antennae and primary mouthparts in *Heteromysis troglophila* sp. nov. **A, C**. Paratype ♀ (**A**) and allotype ♂ (**C**) from Amedien Cave in Guadeloupe; **B, E–J**. Holotype ♀ from Anse Noire Cave in Martinique. **A**. Left female antennula and clypeus, dorsal; **B**. Flagellate spine from disto-mesial corner of antennular trunk in another ♀ (holotype); **C**. Right male antennula and clypeus, dorsal, flagella broken at basis; **D**. Detail of (**C**) showing flagellate spine; **E**. Right antenna with end sac of antennal gland, ventral, setae omitted from antennal scale; **F**. Labrum, somewhat malformed, aboral; **G**. Mandibular palp, caudal aspect; **H, I**. Masticatory parts of left (**H**) and right (**I**) mandibles, rostral; **J**. Labium, obliquely caudal.

***Antennulae*** (Fig. 48A–D). Antennular trunk with setose apophyses and flagella, no teeth. Trunk extends 0.3–0.5× its length beyond eyes. Measured along dorsal midline, the basal segment is 41–48% trunk length, median segment 13–20%, and terminal segment 35–41%. Basal segment on basal half of its outer face with three or four small, barbed setae. Its dorsal apophysis with six barbed setae. The subrectangular lateral apophysis (lobe) reaching to the distal extremity of the median segment in dorsal view but not so in ventral view; its transverse terminal margin with 3–5 barbed setae. Median segment with dorsal apophysis bearing four barbed setae. Terminal segment 1.1–1.3× as long as wide, its mesial half with three or four large plumose setae. Shaft of the flagellate spine at disto-mesial corner with shorter and more acute distal portion in adult male (Fig. 48D) compared with that in both adult females available (Fig. 48A, B). Mid-dorsal apophysis with three or four barbed setae. Appendix masculina (dashed line in Fig. 48C) is 1/5 length of terminal segment. Appendix with brush of smooth setae. Outer antennular flagellum thicker than inner one.

***Antennae*** (Fig. 48E). Antennal sympod with shorter than basally wide, anteriorly projecting lobe (dashed line in Fig. 48E) above peduncle; lateral margin with small spiniform projection at some distance behind the scale. Scale length is 3–4× maximum width; apical segment is 1/10 total length. Basal segment contributes 23–26% to total length of peduncle, median segment 34–35%, and terminal segment 41–43% (*n* = 3).

***Primary mouthparts*** (Fig. 48F–J). Labrum (Fig. 48F) rostrally produced into a short distally rounded triangular process; caudally with small, stiff bristles; fields of setae on caudal and oral faces. Proximal segment of mandibular palp (Fig. 48G) contributing 8–10% palp length, no setae. Length of median segment 3× maximum width and 60–64% palp length. Lateral margin of median segment with two or three smooth whip setae in median position; rostral face of proximal half with 2–4 smaller whip setae with barbed shaft; mesial margin almost all along with 6–9 smooth whip setae; terminal margin with one or two setae. Terminal segment distally well setose, 27–30% palp length. Pars molaris with moderately strong grinding surface in both mandibles. Other parts strongly differing between left and right mandibles (Fig. 48H, I). Left mandible (Fig. 48H) with pars incisiva and digitus mobilis each with four blunt teeth, pars centralis with seven or eight comparatively small spines bearing stiff bristles. Pars incisiva of right mandible (Fig. 48I) with three blunt teeth, digitus mobilis representing a large triangular tooth, and pars centralis with four large acute spines bearing stiff bristles. Labium (Fig. 48J) normal, composed by two lobes with stiff bristles and setae on distal fourth, no spines.

**Figure 49.**
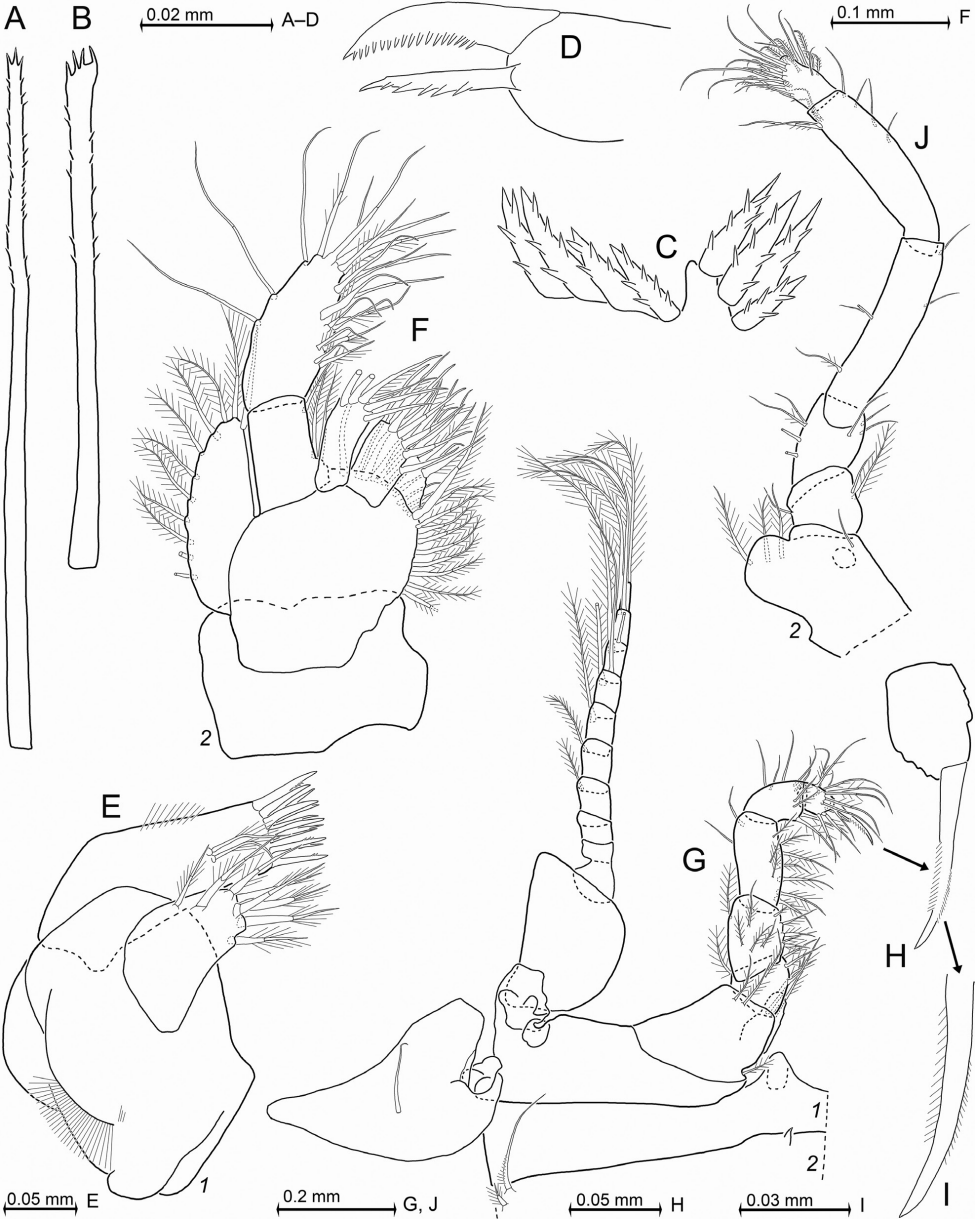
Spines of the foregut and secondary mouthparts in *Heteromysis troglophila* sp. nov. **A–F, J**. Holotype ♀ from Anse Noire Cave in Martinique; **G–I**. Allotype from Amedien Cave in Guadeloupe. **A, B**. Spines of anterior lateralia; **C**. Spine group of posterior lateralia; **D**. Spine pair of dorsolateral infoldings; **E**. Maxillula, caudal; **F**. Maxilla, caudal; **G**. Sternites 1 and 2 (ventral) with thoracopod 1 (caudal) and (somewhat malformed) epipod; **H**. Detail of (**G**) showing dactylus with nail, setae omitted; **I**. Detail of (**H**) showing distal half of nail; **J**. Thoracic endopod 2.

***Foregut*** (Fig. 49A–D). Gross structure as described by Wittmann and Abed-Navandi (2019) for *Heteromysis domusmaris*. Lateralia anteriorly with apically coronate spines (Fig. 49B) and apically pronged spines (Fig. 49A), each with loose series of small denticles along distal 1/3–1/2. Lateralia more caudally with separate cluster of 6–8 bilaterally serrated spines (Fig. 49C). Dorsolateral infoldings with spine group formed by a large spine serrated by dense series of small teeth and additional one or two smaller spines with fewer, larger, loosely set teeth (Fig. 49D). Both dissected foreguts were empty.

***Maxillula*** (Fig. 49E) normal; distal segment terminally with 9–12 strong spines, at least three of which armed with two or three minute subapical teeth; this segment subterminally with two setae furnished with long barbs on their proximal half; no pores detected. Endite terminally with five strong setae bearing stiff bristles; inner margin with one and outer margin with two whip setae, both margins also armed with stiff bristles.

***Maxilla*** (Fig. 49F) normal; terminal segment of endopod 1.8–2.1× as long as maximum width. Lateral margin of exopod all along with eight or nine plumose setae, not counting the apical seta and its mesially adjoining seta which are 1/3 longer than average lateral setae.

**Figure 50.**
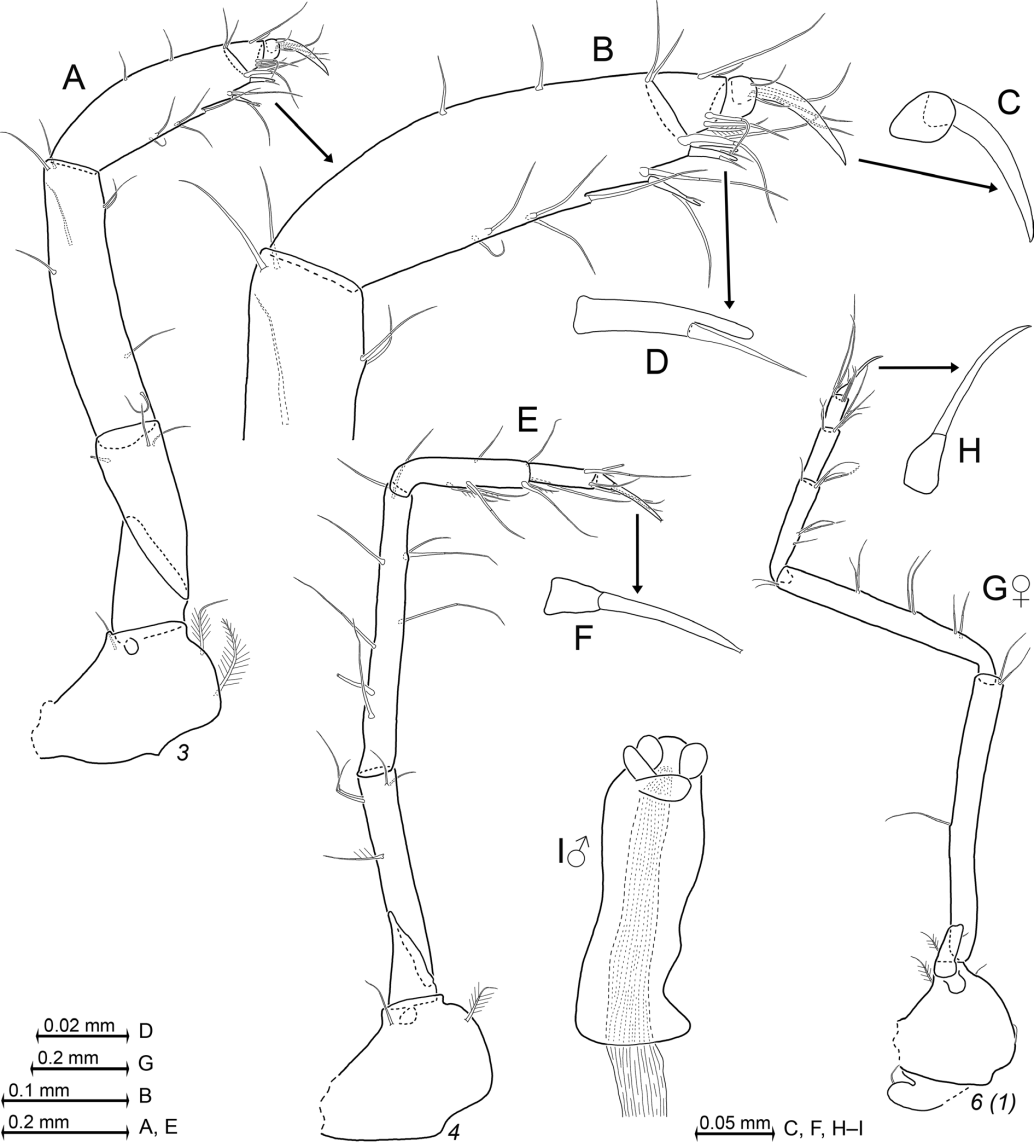
Thoracic endopods 3, 4, 6 and penis in *Heteromysis troglophila* sp. nov. **A–H**. Holotype ♀ from Anse Noire Cave in Martinique; **I**. Allotype ♂ from Amedien Cave in Guadeloupe. **A**. Thoracic endopod 3; **B**. Detail of (**A**) showing tarsus and distal portion of merus; **C, D**. Details of (**B**) showing dactylus with nail (**C**) and flagellate spine (**D**); **E**. Thoracic endopod 4; **F**. Detail of (**E**) showing dactylus with nail; **G**. Thoracic endopod 6 with rudimentary oostegite; **H**. Detail of (**G**) showing dactylus with nail; **I**. Penis. **C, F, H**. Setae omitted from dactylus.

**Figure 51.**
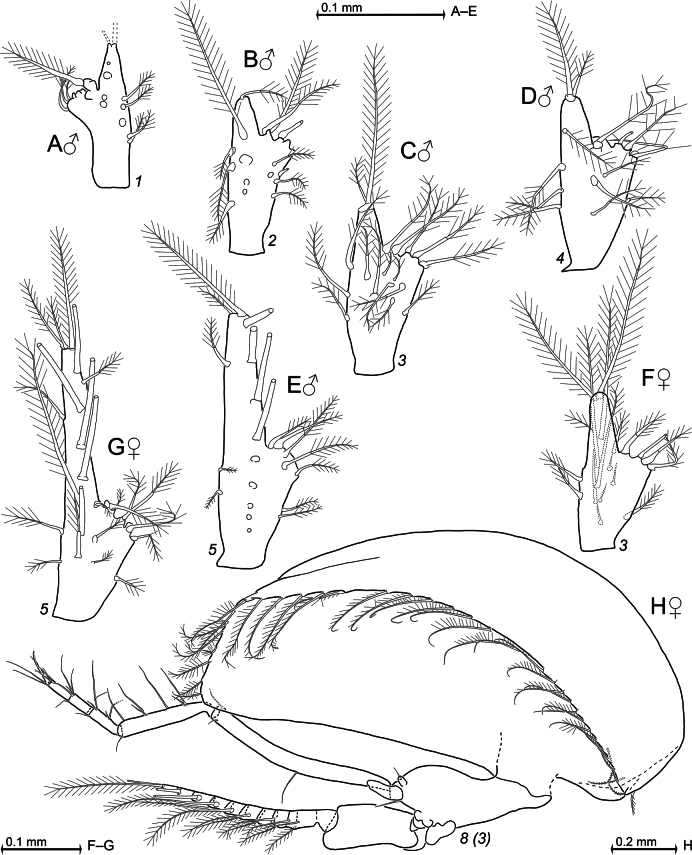
Pleopods and thoracopod 8 with oostegite in *Heteromysis troglophila* sp. nov. **A–E**. Allotype ♂ from Amedien Cave in Guadeloupe; **F–H**. Holotype ♀ from Anse Noire Cave in Martinique. **A–E**. Male pleopods 1–5, ventral = lateral aspect; **F, G**. Female pleopods 3 and 5, mesial aspect; **H**. Thoracopod 8 (rostral) with oostegite 3 (inner = mesial face).

***Thoracopods*** (general; Figs 49G–J, 50A–H, 51H). Flagellum of thoracic exopod 1 with eight segments (Fig. 49G), exopods 2–8 with nine segments (Fig. 51H). Basal plates expanded, 1.5–2.3× as long as wide, distolateral corner well rounded (Figs 49G, 51H). Thoracopod 1 with leaf-like epipod bearing none or one seta. Total length of endopods, including length of ischium, increases in series of endopods 1–6 and then decreases weakly to endopod 8. Endopod 3 (Fig. 50A) measures 1/4 BL and shows somewhat thicker and shorter ischium compared with endopods 4–8. Endopods 4–8 only with smooth paradactylar setae, none on endopod 3. Endopod 1 with strong, weakly bent nail bilaterally lined with stiff bristles (Fig. 49H, I) along part of distal half; endopod 2 without nail, endopods 4–8 with smooth, more slender, weakly bent nail (Fig. 50F, H). Nail 3 approx. as long as nail 1 but 1.4–1.6× as thick at basis, nails 4–8 are 4–15% shorter and only 1/2 as thick as nail 3. When stretched anteriorly, endopod 8 reaches to labrum or clypeus, when stretched posteriorly to pleomeres 3 or 4.

***Maxillipeds*** (Fig. 49G–J). Coxa of first maxilliped (thoracic endopod 1, Fig. 49G–I) with small endite bearing one barbed seta at tip. Basis with comparatively small endite (dashed line in Fig. 49G) that is densely setose on mesial and terminal margins. Ischium to dactylus without endite. Intersegmental joint between thoracic sternite 2 and sympod 2 with large barbed seta as long as 1.2× combined length of praeischium plus ischium of endopod 2. This seta accompanied by a small whip seta with many long barbs (Fig. 49G). Basis of second maxilliped (thoracic endopod 2, Fig. 49J) with mesially projecting endite bearing 1–3 large plumose setae. Combined praeischium plus ischium 0.7× length of merus, carpopropodus plus dactylus 1.2–1.3× merus. Dactylus large, with dense brush formed by large numbers of normal setae and 6–9 modified setae, the latter apically bent, bearing two symmetrical series of stiff cils on either side between 1/5–2/3 length from basis.

***Gnathopods*** (Fig. 50A–D). Thoracic endopod 3 non-dimorphic, forming a strong subchela. Basis with weakly distinct endite bearing one or two barbed setae. Ischium and merus strong, as normal in gnathopods. Ischium 2.7–3.3× as long as wide; merus 4.4–4.6× as long as wide and 1.6–1.7× length of ischium. Carpopropodus 0.8–0.9× the length of merus and 1.2–1.5× ischium. Length of carpus 2.7–2.9× maximum width. Carpus with two flagellate spines at disto-mesial corner and a single flagellate spine at 1/7–1/5 carpus length proximally from this corner on the mesial margin. Propodus 1/6–1/5 carpus length, mesially with two smooth setae and one bilaterally barbed seta. Dactylus 1/10 carpus length, distally with three long and one or two short smooth setae flanking the nail.

***Marsupium*** (Figs 46A, 50G, 51H). The vestigial oostegite 1 (Fig. 50G) without seta. Oostegite 2 with four setae near conjunction with sympod 7, these setae all along hispid by minute stiff barbs; caudal fourth of lower margin with four smooth setae, rostral 3/4 with series of numerous well-barbed setae, most of which unilaterally barbed, only few setae bilaterally barbed. Lower margin of oostegite 3 (Fig. 51H) most caudally with three hispid setae, rostrally followed by dense series of unilaterally barbed setae until rostral fourth of the margin with transition from unilaterally to bilaterally barbed. No setae on outer face of marsupium.

***Pleopods*** (Fig. 51A–G) non-dimorphic, representing setose plates in both sexes, no spines, no teeth. Length increases continuously by an increment of ≈1/15 upon each step from pleopod 1 to 4, whereas 9/10 from 4 to 5. Pleopod 5 (without setae) measures 7% BL in both sexes. Absolute size differences of pleopods related to different BL are compensated in Fig. 51 by using inversely proportional scale length for male with BL = 2.9 mm (panels A–E) vs female with 4.7 mm (panels F, G).

***Tail fan*** (Figs 44J, 44K, 46B). Scutellum paracaudale right-angled triangular with blunt tip (Fig. 46B). Exopod of uropods 1.2–1.4× length of endopod (Fig. 44J). Exopod extends by 20–27% its length beyond endopod and 31–38% beyond telson; endopod 9–17% its length beyond telson. Diameter of fluorite statoliths 77–126 μm (*n* = 6). Statolith formula (2–3) + (1–2) + (0–1) + (5–6) + (4–7) = 13–18 (n = 4). Telson length 1.8–2.0× its maximum width and 1.2–1.4× length of sixth pleomere. Spines at lateral telson margins increase continuously in length distally up to 1/2 length of the spine series and then decrease weakly again in continuous series; the pattern is analogous for the laminae in the cleft (Fig. 46B). The large terminal spines of the distal lobes of the telson (Figs 44K, 46B) measure 15–17% telson length; the disto-mesial spines 0.3–0.4× the length of the large apical spines.

##### Larvae.

The female with BL 4.7 mm carried five nauplioid larvae at substage N2, the female with BL 5.1 mm three larvae at substage N1. Nauplioids with normal gross morphology, body and antennae with smooth cuticle all around, no caudal furca, no spines, no setae.

##### DNA data.

A 623 bp mitochondrial COI sequence was obtained from a single specimen from Amedien Cave (GenBank PV990328). The 801 bp sequence of nuclear 18S (GenBank PV990201) of *H. troglophila* displays highest similarities with other 18S sequences available for *Heteromysis* species in GenBank, closest to an unidentified Japanese species (GenBank AM422515) from which it differs by 2%.

##### Distribution and habitat

(Fig. 10H). Type locality is Martinique, semi-dark area of the Anse Noire Cave, 14°31.70'N, 61°05.30'W, 4 m depth (Station 1), where the mysids occurred in small groups. In Guadeloupe they dwelled solitarily in the dark zone of the Amedien Cave (Station 12), displaying a somewhat reddish color *in situ*. Total distribution range 15–17°N, 61°W, depth 4–6 m.

##### Remarks.

Arguments for affiliation with the genus *Heteromysis* arise from a single flagellate spine at the disto-mesial corner of the antennular trunk, subchelate thoracic endopod 3, normal endopod 8 and reduced setose pleopods in both sexes. Affiliation at subgenus level is unclear.

The new species appears closely related to *H. dispar* Brattegard, 1970, from the Florida Keys based on telson structure and on penis with four lobes. The penis structure of *H. dispar* was first described by Wittmann (2013: fig. 4e). That species differs from the new species by triangular vs broadly rounded rostrum; antennal peduncle reaching halfway vs 2/3–3/4 along antennal scale; thoracic endopod 2 with short nail vs no nail developed; each lateral margin of telson along distal half with 7–11 vs 13–18 spines.

A very similar telson is also found in *H. gymnura* W.M. Tattersall, 1922, from the northern Indian Ocean (W.M. Tattersall 1922; Wittmann and Ariani 2019). The distribution is discussed in Wittmann (2013) and the pleopod structure in Wittmann and Abed-Navandi (2021). *Heteromysis gymnura* differs from the new species by larger eyes and triangular rostrum; unsegmented antennal scale vs presence of a small distal segment; carpopropodus of thoracic endopod 4 with three vs two segments; telson cleft with ≈50 vs 26–40 laminae.

A similar telson structure is also found in *H. komaii* Fukuoka, 2005, from coastal waters of Japan (Fukuoka 2005). It differs from the new species by antennal peduncle reaching to distal 3/7 of antennal scale; carpopropodus of thoracic endopod 3 with two vs three flagellate spines; nearly straight vs distinctly convex distal half of lateral margins of telson; each lateral margin with 10–12 vs 13–18 spines; distally more widely open telson cleft; penes distally with two vs four lobes.

### Keys to genera and species

The inclusion of certain non-Caribbean species is necessary for contrasting information. Distribution of the non-Caribbean species *Palaumysis philippinensis*, *P. pilifera*, *Platyops kumejimensis*, and *P. simplex* is indicated in the respective keys to species below, while for all other species it is discussed in the respective sections above.

### Key to the genera of cave-dwelling Mysidae in the Caribbean

Keys to species given below in secondary keys for the respective genera.

**Table:** 

1	Lateral margin of antennal scale with bare portion distally ending in one tooth (Fig. 2B, E); eyes normal (Fig. 2A)	**2**
–	Lateral margin of antennal scale setose or bare, no spine, no tooth (Figs 6C, 29B, 29F)	**3**
2	Telson subtriangular, terminal margin narrowly truncate with two densely set large spines, each lateral margin with only one small subterminal spine (Fig. 2F); very large eyes, larger than the small, stout antennal scale	***Amathimysis sarbui* Băcescu, 1991**
–	Telson trapezoid with short apical cleft, distal 1/2 of lateral margins with dense series of spines, distolateral edge with pair of moderately large spines, cleft all along armed with laminae (Fig. 2D); eyes normal-sized (Fig. 2A)	***Anchialina typica typica* (Krøyer, 1861)**
3	Cornea completely reduced, eye without pigment (Fig. 4A, D)	**genus *Antromysis* Creaser, 1936**
–	Eyes with well-developed cornea (Figs 29E, 44F) or with at least a few ommatidia (Fig. 29H)	**4**
4	Telson terminally truncate with convex (Fig. 9B, C, E, G) straight (Figs 6D, 29D, 29I, 43D) or weakly indented terminal margin (Figs 9H, 11E)	**5**
–	Telson terminally well indented, incised (Figs 9I, 9K, 9M, 34C, 39I, 44C, 44E, 44H, 44K, 44N)	**10**
5	Telson with convex terminal margin, distal 1/3–1/2 of lateral margins with continuous series of densely-set spines or laminae; spines (laminae) at (mostly rounded) distolateral edge not or only slightly extending beyond neighboring spines (laminae) (Fig. 9C, G)	**genus *Mysidium* Dana, 1852 (in part)**
–	Telson all around bare (Fig. 4F) or with pair of spines on distolateral edges (Figs 11E, 29D, 29I, 29K), these spines longer than all other, if any, spines and laminae (Figs 4B, 6B, 6D)	**6**
6	Each lateral margin of telson with >1 spine, distolateral edges with two longer spines flanking median spines (Fig. 29D, I, K) or laminae (= small tooth-like projections); antennal scale (Fig. 29B) and mesial antennular flagellum well-developed	**8**
–	Telson with bare lateral margins (Fig. 11E); mesial antennular flagellum (Figs 11B, 12B, 12E, 12H, 12K, 12N) and antennal scale (Figs 11C, 12C, 12F, 12I, 12L, 12O) vestigial	**7**
7	Carapace normal-sized, large, leaving ultimate <3 thoracomeres mid-dorsally exposed (Fig. 11A)	***Gironomysis lalanai* Ortiz, García-Debrás & Pérez, 1997**
–	Carapace very short, leaving ultimate 4 or 5 thoracomeres mid-dorsally exposed (Fig. 12A)	**genus *Palaumysis* Băcescu & Iliffe, 1986**
8	Eyes normal, eyestalks subcylindrical with distally positioned cornea dorsoventrally not or only slightly compressed, calotte-shaped in dorsal view (Fig. 6C), (sub)spherical to kidney-shaped in lateral view; distolateral edges of telson with spines flanking median series of 9–16 laminae along straight (Fig. 6D) or slightly concave terminal margin (Fig. 6B)	**genus *Parvimysis* Brattegard, 1969**
–	Eyestalks subquadrate with cornea laterally (Fig. 43A) or distolaterally implanted (Fig. 29A, E, H, J); distolateral edges of telson with long spines flanking clearly shorter (Figs 29D, 29I, 29K, 43D) or (sub)equal (Fig. 29G) spines or laminae	**9**
9	Thoracic endopod 3 pediform (Fig. 29C), non-prehensile, with normal-shaped 2- to 3-segmented carpopropodus, no spine; large flattened eyestalks with (Fig. 29H, J) or without (Fig. 29A, E) spiniform projection	**genus *Platyops* Băcescu & Iliffe, 1986**
–	Thoracic endopod 3 prehensile, subchelate, with swollen unsegmented carpopropodus armed with spines on distal 1/3 of mesial margin (Fig. 43B); distolateral corner of eyestalks with spiniform projection (Fig. 43A)	***Chelitrapezura dennisi* (Bowman, 1985)**
10	Distolateral lobes of telson broadly rounded, lobes armed with continuous series of densely-set spines and laminae (Fig. 9I, K, M)	**genus *Mysidium* Dana, 1852 (in part)**
–	Each distolateral lobe of the telson converging to a narrowly truncate terminus bearing a large spine accompanied by a small (Fig. 44K) or a subequal spine (Fig. 44H, N); lateral margins of telson with mostly intermediate-sized spines at least in distal portions (Fig. 44N); telson cleft at least basally lined with laminae (Figs 34C, 39I), no spines (Fig. 39I) or at most one small subterminal spine (Fig. 34C) inside cleft	**11**
11	Thoracic endopod 3 non-prehensile, not stout, with normal-shaped 2- to 4-segmented carpopropodus (Figs 35A, 41D), no spine; eyes normal	**genus *Bermudamysis* Băcescu & Iliffe, 1986**
–	Thoracic endopod 3 prehensile, mostly stout, with swollen 1- to 2-segmented carpopropodus armed with spines on mesial (= inner) margin (Fig. 44L); cornea normal (Fig. 44F) or reduced to various degrees (Fig. 44A)	**genus *Heteromysis* S.I. Smith, 1873**

### Key to the species of *Antromysis* in Caribbean caves

Fig. 4

**Table:** 

1	Eyestalks closely set together, medially (almost) in contact (Fig. 4A)	**2**
–	Eyestalks widely separated (Fig. 4D)	**3**
2	Telson slightly shorter than maximum width, terminal width 0.2× maximum width near basis; distolateral corners with pair of long spines flanking 0–2 (mostly 0) (para)median spines (Fig. 4C)	***A. peckorum* Bowman, 1977**
–	Telson slightly longer than maximum width, terminal width 0.3–0.5× maximum width; distolateral corners with pair of strong spines flanking 1–3 (mostly 2) small (para)median spines (Fig. 4B)	***A. cenotensis* Creaser, 1936**
3	Telson roughly semicircular with bare margins all around (Fig. 4F)	***A. juberthiei* Băcescu & Orghidan, 1977**
–	Telson roughly trapezoid with strongly convex lateral margins, a pair of spines flanking the truncate terminal margin, no additional spines (Fig. 4E)	***A. cubanica* Băcescu & Orghidan, 1971**

### Key to the species of *Parvimysis* in Caribbean caves

Fig. 6

**Table:** 

1	Rostrum widely rounded; antennal scale with basal segment extending beyond antennular trunk; eyes very large, maximum cornea diameter 1.8–2.4× length of terminal segment of antennular trunk (Fig. 6C); telson terminally truncate (Fig. 6D) or slightly concave	***Parvimysis laminata* Wittmann, 2020**
–	Rostrum subtriangular, apically narrowly rounded; antennal scale with basal segment not reaching to end of antennular trunk; eyes moderately sized, maximum cornea diameter 1.5–1.7× length of terminal segment of antennular trunk (Fig. 6A); telson with weakly concave terminal margin (Fig. 6B)	***Parvimysis brattegardi* Wittmann, 2020**

### Key to the species of *Mysidium* in Caribbean caves

Modified after Wittmann and Wirtz 2019; Fig. 9

**Table:** 

1	Telson linguiform to subrectangular, terminally not emarginated or at most with a rounded, shallow indentation (Fig. 9B, C, E, G, H); male pleopod 4 with 4-segmented exopod (Fig. 9F)	**4**
–	Telson with distinct apical cleft separating two broadly rounded, apical lobes (Fig. 9I, K, M)	**2**
2	Telson with approx. straight lateral margins (Fig. 9I); antennal scale not extending beyond antennular trunk	***M. iliffei* Băcescu, 1991**
–	Telson with distinctly concave lateral margins (Fig. 9K, M); antennal scale extending well beyond antennular trunk (Fig. 9A); male pleopod 4 with 3-segmented exopod (Fig. 9J, L)	**3**
3	Sympod of male pleopod 4 with strong endite at 2/5 sympod length from basis (Fig. 9L); both sexes with blunt laminae on terminal margins of telson and its cleft (Fig. 9M)	**M. (Orientomysidium) columbiae (Zimmer, 1915)**
–	Endite of sympod of male pleopod 4 reduced to indistinct projection (Fig. 9J); both sexes with acute laminae on terminal margin of telson and its cleft (Fig. 9K)	**M. (O.) antillarum Wittmann in Wittmann & Wirtz, 2019**
4	Telson terminally with a shallow, rounded indentation (Fig. 9H)	**M. (Mysidium) gracile (Dana, 1852)**
–	Telson terminally not emarginated (Fig. 9B, C, E, G)	**5**
5	Telson spatulate, its terminal portion triangular with rounded tip (Fig. 9G)	**M. (M.) triangulare Wittmann in Wittmann & Wirtz, 2019**
–	Telson with transverse or with convex, continuously rounded terminal margin (Fig. 9B, C, E)	**6**
6	Cornea diameter <2.5× as long as terminal segment of antennular trunk (Fig. 9A)	**M. (M.) integrum W.M. Tattersall, 1951**
–	Cornea very large, diameter >2.5× as long as terminal segment of antennular trunk (Fig. 9D)	**M. (M.) cubanense Băcescu & Ortiz, 1984**

### Global key to the genera and species of the Palaumysinae

Figs 11, 12

**Table:** 

1	Carapace normal-sized, leaving ultimate <3 thoracomeres mid-dorsally exposed (Fig. 11A)	***Gironomysis lalanai* Ortiz, García-Debrás & Pérez, 1997**
–	Carapace short, leaving ultimate 4–5 thoracomeres mid-dorsally exposed (Fig. 12A): genus *Palaumysis* Băcescu & Iliffe, 1986	**2**
2	The vestigial antennal scale with only 1 or 2 minute setae at apex (Fig. 12O): submarine caves in the Palau Archipelago, tropical W-Pacific	***P. simonae* Băcescu & Iliffe, 1986**
–	The vestigial antennal scale with large seta accompanied by 0–2 small setae at terminus (Fig. 12C, F, I, L)	**3**
3	Antennal scale terminally truncate with two setae at terminus (Fig. 12I, L)	**5**
–	Antennal scale terminally rounded, tip with only one seta (Fig. 12C, F)	**4**
4	Lateral margin of antennal scale with ≥1 short seta (Fig. 12F); apex of male pleopod 4 with large, all along bare seta (Fig. 12G): submarine cave in the Philippines, tropical NW-Pacific	***P. philippinensis* Hanamura & Kase, 2002**
–	Antennal scale with bare lateral margin (Fig. 12C); apex of male pleopod 4 with large setae bearing rows of fine cils near half-length (Fig. 12D): submarine caves in Okinawa, Japan, subtropical NW-Pacific	***P. pilifera* Hanamura & Kase, 2003**
5	Antennula with 2-segmented mesial flagellum (Fig. 12K); apex of antennal scale with long seta measuring <1/2 scale length (Fig. 12L); male pleopod 4 with bare lateral margin (Fig. 12M)	***P. bahamensis* Pesce & Iliffe, 2002**
–	Antennula with 3- to 5-segmented mesial flagellum (Fig. 12H); apex of antennal scale with long seta measuring 0.6–1.0× scale length (Fig. 12I); lateral margin of male pleopod 4 with 0–3 short setae, apex with large seta barbed from median to subapical portions (Fig. 12J)	***P. antillensis* sp. nov**.

### Global key to the species of *Platyops*

Fig. 29

**Table:** 

1	Eyestalk with bare anterior margin (Fig. 29A, E), no distal process; antennal scale with (Fig. 29B) or without (Fig. 29F) small distal segment	**2**
–	Eyestalk with small sharp process (Fig. 29H, J) at anterior margin; antennal scale unsegmented (Fig. 29F)	**3**
2	Antennal scale with small distal segment (Fig. 29B); telson trapezoid, length without spines 1.3–1.5× maximum width near basis, transversely truncate terminal margin with pair of large distolateral smooth spines flanking a paramedian pair of somewhat shorter spines (Fig. 29D)	***P. sterreri* Băcescu & Iliffe, 1986**
–	Antennal scale unsegmented (Fig. 29F); telson linguiform, length 1.6–1.8× maximum width near basis, convex terminal margin laterally with 3–4 pairs of long, weakly serrate spines flanking a paramedian pair of (sub)equal spines (Fig. 29G): submarine cave of Kumejima Island, Japan, NW-Pacific	***P. kumejimensis* (Shimomura & Fujita, 2020), comb. nov**.
3	Cornea compact, composed of numerous, densely set ommatidia (Fig. 29J); telson trapezoid, length 1.8× maximum width near basis (Fig. 29K)	***P. stenoura* (Hanamura & Kase, 2004)**
–	Eyes with <20 ommatidia, together not forming a compact cornea (Fig. 29H); telson trapezoid, almost triangular, length <1.5× maximum width near basis (Fig. 29I): submarine caves of Okinawa, Japan, NW-Pacific	***P. simplex* (Hanamura & Kase, 2001)**

### Global key to the species of *Bermudamysis*

**Table:** 

1	Thoracic endopod 3 with 2- to 3-segmented carpopropodus (Fig. 35A), endopods 6–8 with 3-segmented carpopropodus (Fig. 35D, E); penes 0.4–0.6× length of ischium 8 (Fig. 35F vs Fig. 35E)	***B. speluncola* Băcescu & Iliffe, 1986**
–	Thoracic endopod 3 with 3- to 4-segmented carpopropodus (Fig. 41D), endopods 6–8 with 4- to 6-segmented carpopropodus (Fig. 41I, J); penes 1.2–1.5× length of ischium 8 (Fig. 41K vs 41J)	***B. caribbaea* sp. nov**.

### Key to the species of *Heteromysis* in Caribbean caves

Modified after Wittmann and Wirtz 2019; Fig. 44

**Table:** 

1	Eyestalks subrectangular to oval in dorsal view, dorsoventrally compressed, distal 1/5 obliquely truncate in lateral view, small oval cornea laterally in subterminal position (Fig. 44A)	***H. spongicola* (Băcescu, 1968)**
–	Eyes normal, cornea large, calotte-shaped, terminally or distolaterally positioned on eyestalk (Fig. 44F)	**2**
2	Lateral margins of telson all along with spines (Fig. 44E); disto-mesial corner of antennular trunk with one blunt seta, mesially finely barbed and with distal pair of minute flagella (arrow in Fig. 44D)	**H. (Heteromysis) cyanogoleus Bamber, 2000**
–	Proximal third of telson with bare lateral margins (Fig. 44H, K, N); disto-mesial edge of antennular trunk with a flagellate spine in various modifications characterized by a thick or flattened handle and a thin subapically inserting flagellum (arrow in Fig. 44I, Fig. 48B, D)	**3**
3	Endopod of uropods without spines (Fig. 44J); proximal 2/3–3/4 of telson cleft bearing 36–40 laminae; each distolateral lobe of telson ending in a large spine mesially accompanied by a small spine (Fig. 44K), not counting a potential lateral spine (Fig. 46B)	***H. troglophila* sp. nov**.
–	Endopod of uropods armed with >10 spines along mesial margin between statocyst and tip (Fig. 44G, M); proximal ≤1/2 of telson cleft with total of ≤20 laminae; each distolateral lobe of telson ending in two moderately large, subequal spines (Fig. 44H, N)	**4**
4	Endopod of uropods not curved (Fig. 44G); telson cleft with 14–20 laminae along basal half (Fig. 44H)	**H. (Olivemysis) bermudensis G.O. Sars, 1885**
–	Endopod of uropods curved mesially (Fig. 44M); telson cleft with 9–12 laminae on basal third (Fig. 44N)	**H. (O.) floridensis Brattegard, 1969**

## Supplementary Material

XML Treatment for Anchialina typica typica

XML Treatment for Amathimysis sarbui

XML Treatment for Antromysis

XML Treatment for Antromysis cenotensis

XML Treatment for Antromysis cubanica

XML Treatment for Antromysis juberthiei

XML Treatment for Antromysis peckorum

XML Treatment for Parvimysis

XML Treatment for Parvimysis brattegardi

XML Treatment for Parvimysis laminata

XML Treatment for Mysidium

XML Treatment for Mysidium (Mysidium) cubanense

XML Treatment for Mysidium (Mysidium) gracile

XML Treatment for Mysidium (Mysidium) integrum

XML Treatment for Mysidium (Mysidium) triangulare

XML Treatment for Mysidium (Orientomysidium) antillarum

XML Treatment for Mysidium (Orientomysidium) columbiae

XML Treatment for Mysidium iliffei

XML Treatment for Palaumysinae

XML Treatment for Gironomysis

XML Treatment for Gironomysis lalanai

XML Treatment for Palaumysis

XML Treatment for Palaumysis simonae

XML Treatment for Palaumysis bahamensis

XML Treatment for Palaumysis antillensis

XML Treatment for Mysidetini

XML Treatment for Platyops

XML Treatment for Platyops sterreri

XML Treatment for Platyops stenoura

XML Treatment for Bermudamysis

XML Treatment for Bermudamysis speluncola

XML Treatment for Bermudamysis caribbaea

XML Treatment for Chelitrapezura

XML Treatment for Chelitrapezura dennisi

XML Treatment for Heteromysis

XML Treatment for Heteromysis spongicola

XML Treatment for Heteromysis (Heteromysis) cyanogoleus

XML Treatment for Heteromysis (Olivemysis) bermudensis

XML Treatment for Heteromysis (Olivemysis) floridensis

XML Treatment for Heteromysis troglophila

## Additional information

### Artificial Intelligence (AI) use

The authors accept full responsibility for the content of the manuscript, including the disclosure of any use of AI.

No AI tools were used in the preparation of this manuscript.

### Author ORCIDs

K.J. Wittmann https://orcid.org/0000-0003-1381-2588

P. Chevaldonné https://orcid.org/0000-0003-0507-7747

### Data availability

All of the data that support the findings of this study are available in the main text.
